# Catalytic asymmetric hydrometallation of cyclobutenes with salicylaldehydes†

**DOI:** 10.1039/d1sc06035j

**Published:** 2021-12-10

**Authors:** F. Wieland Goetzke, Mireia Sidera, Stephen P. Fletcher

**Affiliations:** Department of Chemistry, University of Oxford 12 Mansfield Road Oxford OX1 3TA UK stephen.fletcher@chem.ox.ac.uk; Vertex Pharmaceuticals (Europe) Ltd 86–88 Jubilee Avenue Milton Park, Abingdon OX14 4RW UK

## Abstract

Chiral, substituted cyclobutanes are common motifs in bioactive compounds and intermediates in organic synthesis but few asymmetric routes for their synthesis are known. Herein we report the Rh-catalyzed asymmetric hydrometallation of a range of *meso*-cyclobutenes with salicylaldehydes. The *ortho*-phenolic group promotes hydroacylation and can be used as a handle for subsequent transformations. The reaction proceeds *via* asymmetric hydrometallation of the weakly activated cyclobutene, followed by a C–C bond forming reductive elimination. A prochiral, spirocyclic cyclobutene undergoes a highly regioselective hydroacylation. This report will likely inspire the development of other asymmetric addition reactions to cyclobutenes *via* hydrometallation pathways.

## Abstract 2

Chiral, substituted cyclobutanes are common motifs in bioactive compounds and intermediates in organic synthesis but few asymmetric routes for their synthesis are known.

## Graphical abstract

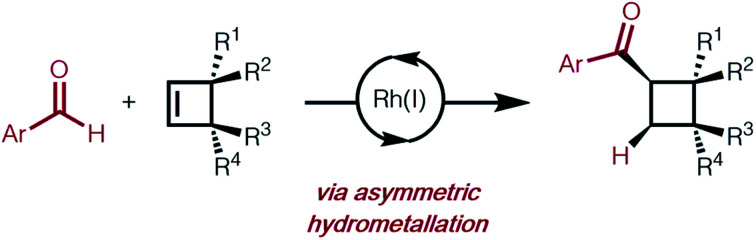

## Introduction

**Fig. 1:**
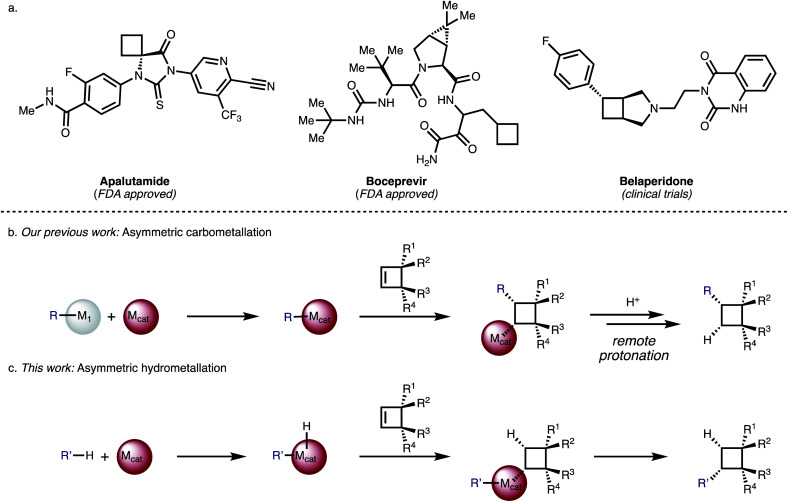
(a) Examples of bioactive cyclobutanes. (b) Asymmetric carbometallation of cyclobutenes. (c) Asymmetric hydrometallation of cyclobutenes.

Substituted cyclobutanes can be found in many natural products,^1^ and are useful building blocks in synthetic chemistry but their asymmetric synthesis is often challenging.^2^ The cyclobutane motif has also received increased attention as a rigid scaffold and as an isostere in medicinal chemistry.^3^ So far 8 compounds that bear a cyclobutane ring have been approved by the FDA but their cyclobutyl moieties are generally simply substituted like in Boceprevir or Apalutamide (Fig. 1a) and none of them bear any stereogenic centres – which may be due to a scarcity of generally useful methods for the synthesis of complex, chiral cyclobutanes.^4,5^

Most existing catalytic asymmetric approaches for cyclobutane synthesis rely either on ring-closure,^6–13^ or the functionalization of pre-formed four-membered rings using activating or directing groups.^14–16^ Direct addition reactions to unactivated cyclobutenes are rare,^17–19^ but attractive as they offer a modular entry to functionalized cyclobutanes and are generally not limited to specific substitution patterns.

We have reported Rh-catalysed asymmetric addition reactions of arylboronic acids to various cyclobutenes (Fig. 1b).^20^ These reactions proceed *via* an asymmetric carbometallation step, followed by remote protonation or elimination to give a diverse range of arylated cyclobutanes. For cyclobutenes, the carbometallation step is associated with a very small release of olefinic strain (1.9 kcal mol^−1^) compared to the olefinic strain of other small, cyclic molecules like cyclopropene (27.7 kcal mol^−1^).^21^ We became interested in the question if related but mechanistically distinct cyclobutene functionalization reactions with carbon-nucleophiles would be possible and identified an asymmetric hydrometallation-reductive elimination sequence as a viable strategy (Fig. 1c). A key advantage of this strategy is its high-atom economy and the avoidance of sensitive organometallic coupling partners.

Metal-catalysed hydroacylation,^22^ and especially Rh-catalysed hydroacylation reactions between alkenes and aldehydes are powerful tools for the synthesis of ketones and operate *via* a hydrometallation mechanism.^23^ While intramolecular hydroacylation reactions are well established, the intermolecular Rh-catalysed hydroacylation is often associated with an undesired reductive decarbonylation, and many specific solutions for this problem involving chelating groups have been developed.^23^ The use of *ortho*-hydroxybenzaldehydes (salicylaldehydes) represents one of the strategies and several useful asymmetric hydroacylations with terminal alkenes have been reported.^24–26^

Internal alkenes represent significantly more challenging substrates in Rh-catalysed intermolecular hydroacylations, but a few reactions between norbornadienes or cyclopropenes and salicylaldehydes have been reported by the groups of Bolm and Dong, which are likely driven by the release of olefinic-strain.^27,28^ Catalytic asymmetric carbofunctionalization reactions of strained alkenes that proceed *via* hydrometallation have also been reported with Nickel.^29^ We wondered if a related process would be possible with cyclobutenes – despite the very small release of ring strain in the hydrometallation step.

## Results and discussion

We chose salicylaldehyde 1a and cyclobutene 2 as our model substrates as they provide an entry to the bicyclic core of Belaperidone (see Fig. 1a). Our optimisation studies revealed that a catalytic system generated from [Rh(cod)OH]_2_ and MeDuphos (L1) gives 3a in excellent yield, enantio- and diastereoselectivity (Table 1, entry 1). Prolonged reaction times led to a slight decrease in diastereomeric ratio. Under these conditions, salicylaldehyde 1a was fully consumed, and decarbonylation of the aldehydes served as the major side-reaction (indicated by the disappearance of CHO signals in the ^1^H NMR spectra).^30^ The reaction does not proceed in absence of either Rh or phosphine ligand (Table 1, entries 2 and 3), while other ligand scaffolds provided only poor levels of enantioinduction in this reaction despite inducing high diastereomeric ratios (Table 1, entries 4–6). Slightly lower diastereoselectivity and yield was obtained with THF instead of toluene (Table 1, entry 7). Using benzaldehyde instead of salicylaldehyde did not provide the desired hydroacylation product (Table 1, entry 8) – highlighting the importance of a chelating group.

**Table 1:** Deviation from standard conditions^a^

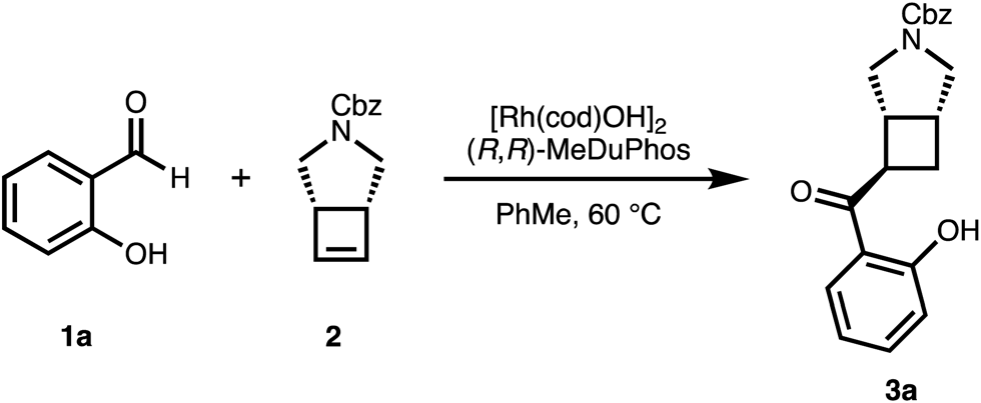					
Entry	Variation from standard conditions	Time (h)	Yield^b^ (%)	ee^c^ (%)	dr^d^
1	None^e^	1	81	98	9 : 1
2	No Rh	20	0	—	—
3	No ligand	20	≤1	—	—
4	L2 instead of L1	20	86	32	>20 : 1
5	L3 instead of L1	2	86	−74	>20 : 1
6	L4 instead of L1	20	31	−12	>20 : 1
7	THF instead of PhMe	1	72	98	7 : 1
8	PhCHO instead of 1a	20	0%	—	—
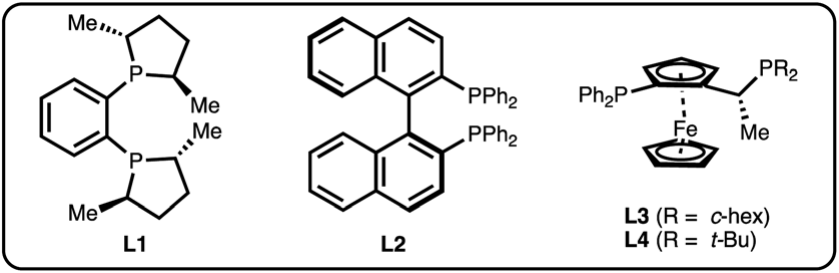					

a [Rh(cod)OH]_2_ (2.5 mol%), ligand (6 mol%), cyclobutene 2 (0.3 mmol), salicylaldehyde 1a (0.2 mmol), PhMe (0.2 M), 1–20 h.

b Isolated yield of the major diastereoisomer.

c The ee values were determined by SFC analysis on a chiral non-racemic stationary phase.

d The dr values were estimated by non-calibrated SFC analysis of the unpurified reaction mixture.

e Performed on 0.4 mmol scale.

**Scheme 1:**
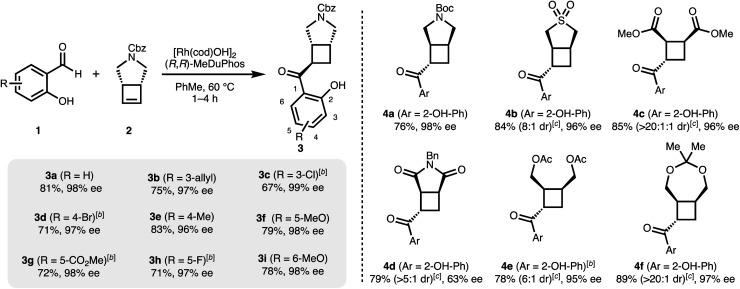
Asymmetric hydroacylation of cyclobutenes with different salicylaldehydes.^*a a*^[Rh(cod)OH]_2_ (2.5 mol%), MeDuphos (6 mol%), cyclobutene 2 (0.6 mmol), salicylaldehyde 1 (0.4 mmol), PhMe (0.2 M), 1–4 h. ^*b*^Increased catalyst loading of [Rh(cod)OH]_2_ (5 mol%) and MeDuphos (12 mol%). ^*c*^Diastereomeric ratios of the unpurified reaction mixtures determined by ^1^H NMR spectroscopy. All yields refer to isolated yields of the major *trans*–*cis* diastereomer. Enantiomeric excesses determined by SFC analysis on a chiral non-racemic stationary phase.

Having optimized conditions for 1a and 2 in hand, we subsequently explored the scope of the transformation on both the salicylaldehyde and the cyclobutene component. Several substitution patterns, electron-withdrawing and electron-donating functional groups, and halides are well tolerated with consistently excellent levels of enantioinduction (Scheme 1, 3a–3i). In all cases, we isolated the pure *cis–trans* isomer.‡‡A precise determination of the diastereomeric ratios of the unpurified reaction mixture by ^1^H NMR was not possible due to the broad (rotameric) peak shapes. However, these crude NMR spectra suggest similar diastereomeric ratios for compounds 3a–3i and 4a (approximately 7 : 1 to 10 : 1). Remarkably, our catalytic system shows high chemoselectivity for the hydroacylation of the cyclobutene over a terminal alkene (3b).^24^ For more electron-deficient salicylaldehydes 1c, 1d, 1g and 1h, we observed unreacted aldehyde accompanied with decarbonylation under our standard reaction conditions. In these cases, better results and full conversion of the aldehyde was achieved with an increased catalyst loading of 5% of dimeric [Rh(cod)OH]_2_.

A range of different mono- and bicyclic *meso*-cyclobutenes are suitable substrates and give acylated cyclobutanes in good yields and in most cases with high enantiomeric excesses (Scheme 1, 4a–4f). For all compounds, the major *trans*–*cis* diastereomer was isolated in pure form. The absolute configuration of 4b was determined *via* X-ray crystallographic analysis. In our previous carbometallation study, (*cis*-cyclobut-3-ene-1,2-diyl)bis (methylene) diacetate (2e) underwent homo-allylic substitution reactions instead of hydroarylations with arylboronic acids.^20^ Using the same substrate under our hydroacylation conditions we obtain the hydroacylation product 4e – highlighting the difference between the carbometallation and hydrometallation pathways (*cf.*Fig. 1b and c).

**Scheme 2:**
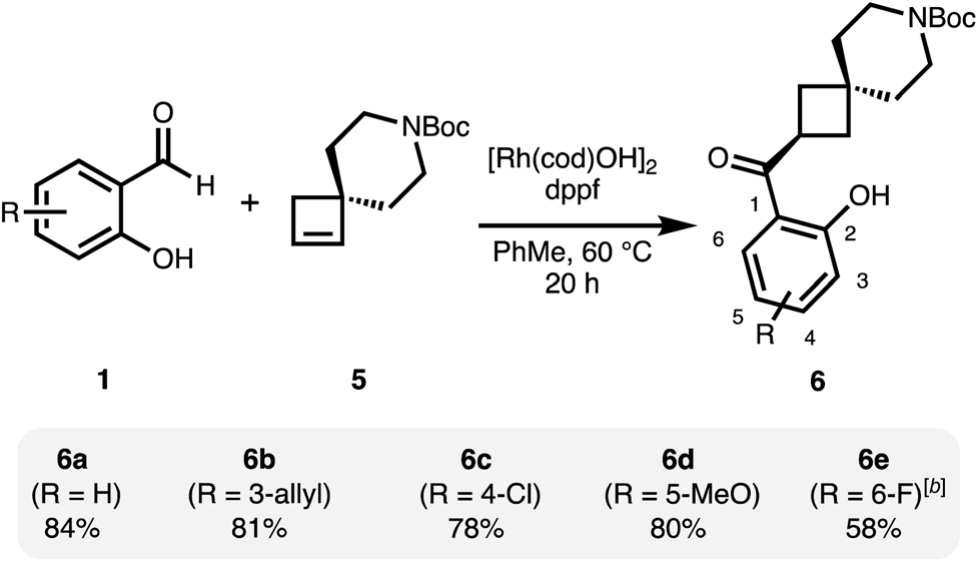
Regioselective hydroacylation of a spirocyclic, prochiral cyclobutene.^*a a*^[Rh(cod)OH]_2_ (2.5 mol%), dppf (6 mol%), cyclobutene 5 (0.6 mmol), salicylaldehyde 1 (0.4 mmol), PhMe (0.2 M), 20 h. ^*b*^Increased catalyst loading of [Rh(cod)OH]_2_ (5 mol%) and dppf (12 mol%).

Under related conditions using achiral ligand 1,1′-bis(diphenylphosphino)ferrocene (dppf), the achiral, spirocyclic cyclobutane 6 is obtained from 5 as a single regioisomer (Scheme 2).^31^ Good yields were obtained with a small set of functionalized salicylaldehydes.

Also here, more electron-deficient salicylaldehydes were prone to an undesired decarbonylation pathway and therefore gave diminished yields (6e). The regioselectivity in this reaction is likely under steric control and is set in the initial hydrometallation step.

**Scheme 3:**
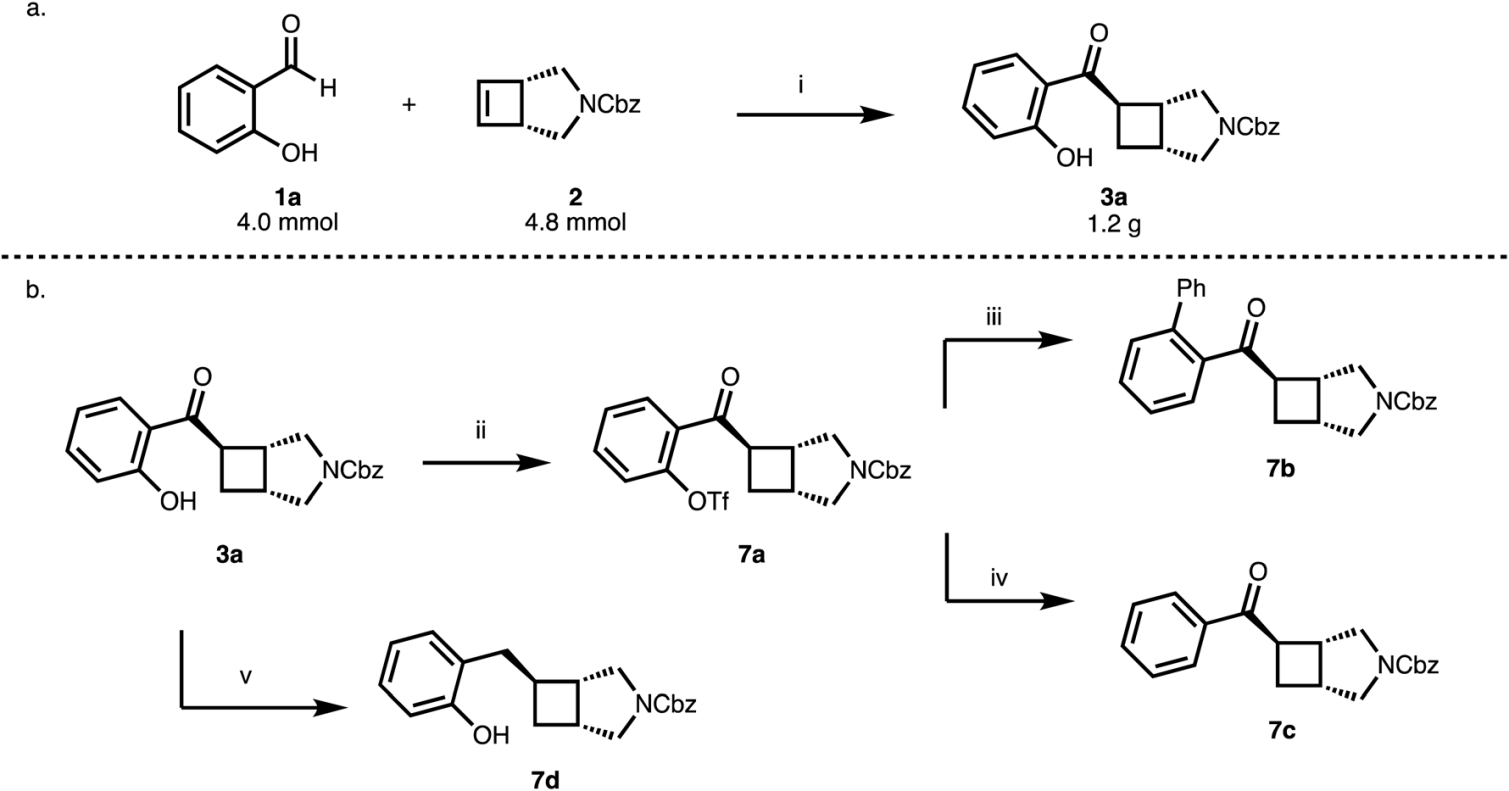
(a) Up-scale to gram-scale. (b) Functionalization of the phenol and benzoyl moiety.^*a a*^(i) [Rh(cod)OH]_2_ (2.5 mol%), MeDuphos (6 mol%), 2 (4.8 mmol), 1a (4.0 mmol), PhMe, 1 h, 60 °C, 84%, 98% ee; (ii) 2-PyrNTf_2_, DMAP (10 mol%), NEt_3_, CH_2_Cl_2_, 23 h, 23 °C, 92%; (iii) PhB(OH)_2_, K_2_CO_3_, [Pd(PPh_3_)_4_] (5 mol%), PhMe, 16 h, 110 °C, 67%; (iv) (CH_3_)_2_NH·BH_3_, K_2_CO_3_, [Pd(PPh_3_)_4_] (5 mol%), CH_3_CN, 6 h, 40 °C, 67%; (v) Et_3_SiH, TFA, 5 h, 0–23 °C, 65%.

The reaction of 1 with 2a proceeds nicely at a 4 mmol scale gram-scale providing 1.2 g (84%, 98% ee) of 3a (Scheme 3a) while lowering the excess of cyclobutene from 1.5 to 1.2 equivalents. The 2-hydroxybenzoyl moiety could serve as a handle for subsequent functionalization reactions (7a, 7b) and the phenolic OH group can be removed in a two-step protocol (7c) (Scheme 3b). Furthermore, reduction of the benzoyl group provides an entry to benzylated cyclobutanes (7d) (Scheme 3b).

## Conclusions

In summary, we have shown that the addition of Rh–H to weakly activated cyclobutenes is possible – exemplified with an asymmetric hydroacylation reaction of *meso*-cyclobutenes with salicylaldehydes. Furthermore, the hydroacylation of a spirocyclic cyclobutene proceeds with excellent regioselectivity. These reactions provide a modular entry to stereochemically complex, acylated cyclobutanes. Likely, other asymmetric Rh-catalysed addition reactions, that proceed *via* hydrometallation pathways, are feasible with cyclobutenes and those will be investigated in the future.

## Data availability

Crystallographic data for 4b has been deposited at the CCDC under 2116804 and can be obtained from http://www.ccdc.cam.ac.uk. All other data supporting this article have been uploaded as part of the supplementary material.

## Author contributions

F. W. G. performed all experiments and conceived the study. S. P. F. and M. S. guided the research. F. W. G. and S. P. F. wrote the manuscript with contributions from M. S.

## Conflicts of interest

There are no conflicts to declare.

## Supplementary Material

SC-013-D1SC06035J-s001

SC-013-D1SC06035J-s002
