# Correction: Catalytic asymmetric hydrometallation of cyclobutenes with salicylaldehydes

**DOI:** 10.1039/d1sc90265b

**Published:** 2022-01-12

**Authors:** F. Wieland Goetzke, Mireia Sidera, Stephen P. Fletcher

**Affiliations:** Department of Chemistry, University of Oxford 12 Mansfield Road Oxford OX1 3TA UK stephen.fletcher@chem.ox.ac.uk; Vertex Pharmaceuticals (Europe) Ltd 86–88 Jubilee Avenue, Milton Park Abingdon OX14 4RW UK

## Abstract

Correction for ‘Catalytic asymmetric hydrometallation of cyclobutenes with salicylaldehydes’ by F. Wieland Goetzke *et al.*, *Chem. Sci.*, 2022, DOI: 10.1039/d1sc06035j.

The authors regret that the absolute stereochemistry in Scheme 1 presented in the original manuscript was incorrect. The correct version of Scheme 1 is shown below.

**Scheme 1:**
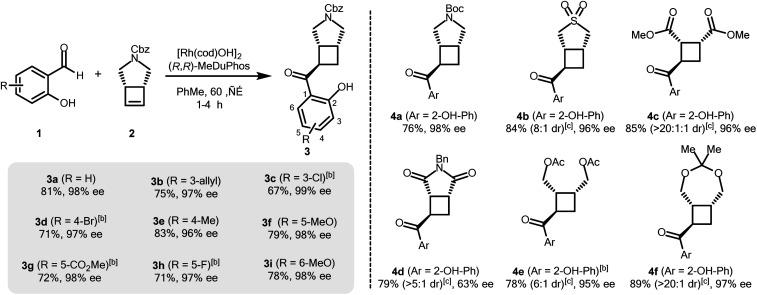
Asymmetric hydroacylation of cyclobutenes with different salicylaldehydes.^*a a*^[Rh(cod)OH]_2_ (2.5 mol%), MeDuphos (6 mol%), cyclobutene **2** (0.6 mmol), salicylaldehyde **1** (0.4 mmol), PhMe (0.2 M), 1–4 h. ^*b*^Increased catalyst loading of [Rh(cod)OH]_2_ (5 mol%) and MeDuphos (12 mol%). ^*c*^Diastereomeric ratios of the unpurified reaction mixtures determined by ^1^H NMR spectroscopy. All yields refer to isolated yields of the major *trans*–*cis* diastereomer. Enantiomeric excesses determined by SFC analysis on a chiral non-racemic stationary phase.

The Royal Society of Chemistry apologises for these errors and any consequent inconvenience to authors and readers.

## Supplementary Material

