# Supplementary material for: Catalytic asymmetric hydrometallation of cyclobutenes with salicylaldehydes
Source: Chem Sci. 2021 Dec 10;13(1):236–40. doi: 10.1039/d1sc06035j (PMC8694367; doi:10.1039/d1sc06035j)
Supplement: SC-013-D1SC06035J-s001 [file SC-013-D1SC06035J-s001.pdf]

Electronic Supplementary Material (ESI) for Chemical Science.
This journal is © The Royal Society of Chemistry 2021

Catalytic Asymmetric Hydrometallation of Cyclobutenes with Salicylaldehydes

F. Wieland Goetzke[a], Mireia Sidera Portela[b], Stephen P. Fletcher[a]

[a]*Department of Chemistry, Chemistry Research Laboratory University of Oxford, 12 Mansfield Road, Oxford, OX1 3TA (UK).*

[b] *Vertex Pharmaceuticals (Europe) Ltd 86–88 Jubilee Avenue, Milton Park, Abingdon, OX14 4RW (UK).*

Supplementary Information

Table of Content

1. Experimental procedures 2
   1.1. General Methods 2
   1.2. Synthesis of the starting materials 4
   1.3. Hydroacylation reactions 6
   1.4. Upscale and derivatization of products 28
2. References 34
3. NMR spectra 35
4. SFC traces 70
5. X-ray crystallographic analysis 87

1. Experimental procedures

1.1. General Methods

All reactions were carried out in anhydrous solvents with continuous magnetic stirring under an inert argon atmosphere. Heating was performed using DrySyn heating blocks.

Nuclear magnetic resonance (NMR) spectroscopy measurements were carried out at room temperature. ^{1}H NMR, ^{13}C NMR, ^{19}F NMR, COSY, HSQC, and NOESY experiments were carried out using Bruker AVIII HD 400 (400/100 MHz) or Bruker NEO 600 (600/150 MHz) spectrometers. Chemical shifts (δ) are reported in ppm relative to the residual solvent peak with corresponding coupling constants (*J*) in Hertz (Hz) and multiplicities (s: singlet, d: doublet, t: triplet, q: quartet, m: multiplet and combinations of these and app.: apparent multiplicities). Assignment follows HSQC, COSY, or/and NOESY spectra, chemical shift and coupling constant analysis.

Optical rotations ($[\alpha]_{25}^{D}$) were recorded using a Perkin Elmer-241 Polarimeter. Concentrations (*c*) are reported in g/100 mL.

Infrared (IR, neat or thin film): spectroscopy was carried out on a Bruker Tensor 27 FT–IR spectrometer with an internal calibration range of 4000–600 cm^{-1}. The samples are reported as absorption maxima in cm^{-1} with corresponding relative intensities described as br (broad), s (strong), m (medium), and w (weak).

Chiral SFC (supercritical fluid chromatography) separations were conducted on a Waters Acquity UPC2 system using Waters Empower software. Chiralpak® columns (150x3 mm, particle size 3 µm) were used as specified in the text. Solvents used were of HPLC grade (Fisher Scientific, Sigma Aldrich or Rathburn).

High Resolution Mass spectra were carried out by internal service at the University of Oxford. (1) Electron spray ionisation (ESI^{+}) was recorded on a Fisons Platform II. (2) Atmospheric pressure chemical ionisation (APCI^{+}): Analyses were performed using a Thermo Exactive mass spectrometer equipped with Waters Acquity liquid chromatography system.

Commercially available reagents and ligands were purchased from Sigma Aldrich, Alfa Aesar, Acros Organics, Fluorochem and Strem Chemicals and unless otherwise stated were used without further

purification. $[Rh(cod)OH]_2$ was bought from Sigma Aldrich. Benzaldehyde was distilled prior to use and ^{1}H NMR indicated a purity of approximately 99%. Dry solvents were collected fresh from an mBraun SPS–800 solvent purification system after having passed through anhydrous alumina columns. Deuterated solvents were purchased from Sigma Aldrich.

Medium-pressure chromatography was performed on a CombiFlash Next Gen 100 system.

The absolute and relative stereochemistry of **4b** was assigned via XRD. The relative stereochemistry of **3a'**, **4b**, **4c**, **4d**, **4e** and **4f** was further assigned by NOESY spectroscopy. The relative and absolute stereochemistry of all other compounds was assigned in analogy to **4b**, and all compounds show the same sign of optical rotation.

1.2. Synthesis of the starting materials

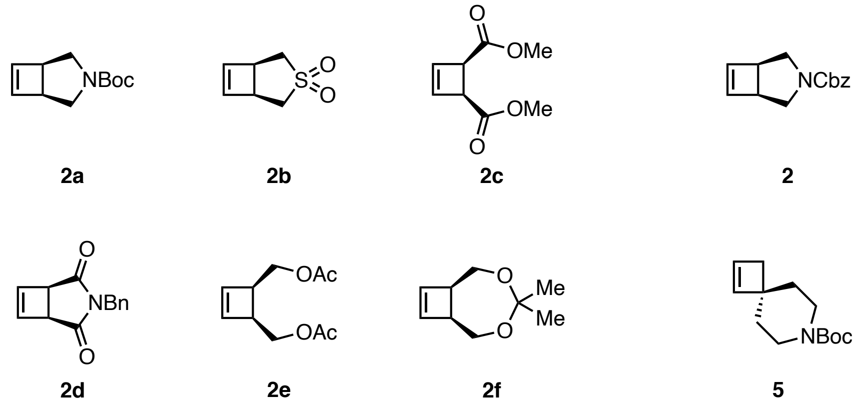

Figure S1: Cyclobutenes used in this study.

Cyclobutenes **2**, **2b**, **2c**, **2d**, **2e** and **5** were synthesized according to our reported procedures.[1] All salicylaldehydes were purchased from Sigma Aldrich, Alfa Aesar, Acros Organics, and Fluorochem were used without further purification. *Cis*-cyclobut-3-ene-1,2-diyl)dimethanol was prepared according to reported procedures.[1,2]

Synthesis of S1

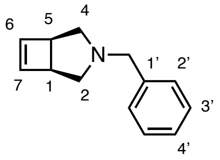

A solution of $LiAlH_4$ in Et_2O (1.0 M, 8.0 mL, 8.0 mmol) was added to a solution of **2d** (426 mg, 2.0 mol) in CH_2Cl_2 (18 mL) at 0 °C under an argon atmosphere. The reaction mixture was allowed to reach room temperature (23 °C) over night and was stirred for 16 h. Then, H_2O (0.4 mL), an aq. solution of NaOH (15 wt%, 0.4 mL), additional H_2O (1.0 mL), followed by $MgSO_4$ were added slowly at 0 °C. The mixture was filtered and solvents were removed *in vacuo*. Purification by automated medium-pressure chromatography (EtOAc) afforded the product **S1** as a yellow oil (328 mg, 88% yield).

^{1}H NMR ($CDCl_3$, 400 MHz): δ (ppm) 7.38 – 7.27 (m, 4H; 2x C(2')–H, 2x C(3')–H), 7.26 – 7.20 (m, 1H; C(4')–H), 6.05 (d, *J* = 0.7 Hz, 1H; C(6)–H, C(7)–H), 3.70 (s, 2H; CH_2Ph), 3.28 – 3.22 (m, 2H; C(1)–H, C(5)–H), 2.89 (d, *J* = 9.9 Hz, 2H; 1x C(2)–H_2, 1x C(4)–H_2), 1.88 (ddd, *J* = 9.9, 5.0, 1.1 Hz, 2H; 1x C(2)–H_2, 1x C(4)–H_2).

^{13}**C NMR** ($CDCl_3$, 101 MHz): δ (ppm) 138.9 (C(1')), 138.0 (C(6), C(7)), 129.0, 128.1, 126.7 (C(4')), 59.6 (CH_2Ph), 53.8 (C(1), C(5)), 47.0 (C(2), C(4)).

IR (neat): 3033 (w), 2941 (w), 2777 (w), 1453 (w), 1345 (w), 1265 (w), 1172 (w), 1157 (w), 864 (w), 758 (s), 735 (m), 697 (s), 631 (m) cm^{-1}.

HRMS (ESI): m/z calcd for $C_{13}H_{16}N^+$ $[M + H]^+$ 186.1277 found 186.1279.

Synthesis of 2a

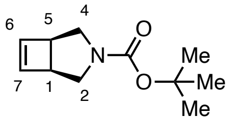

1-Chloroethyl chloroformate (220 µL, 2.0 mmol) was added to a solution of **S1** (278 mg, 1.50 mmol) in CH_2Cl_2 (15 mL) under an argon atmosphere. The mixture was stirred under reflux for 2 h. Then, the solvent was removed under reduced pressure. MeOH (15 mL) was added, and the mixture was stirred under reflux for 1.5 h. The solvent was removed under reduced pressure. Dioxane (7 mL) was added, and the mixture was cooled to at 0 °C. An aq. solution of KOH (1 M, 7 mL), followed by Boc_2O were added, and the reaction mixture was allowed to reach room temperature (23 °C) over night and was stirred for 18 h. The mixture was diluted with Et_2O (20 mL) and H_2O (20 mL). The organic layer was separated, and aqueous layer was extracted with Et_2O (2x 20 mL). The combined organic layers were washed with brine (10 mL) and dried over $MgSO_4$. Purification by automated medium-pressure chromatography (hexane/EtOAc = 100/0 to 80/10) afforded the product **2a** as a colorless oil (230 mg, 78% yield). The analytical data are in agreement with the literature.[1]

^{1}**H NMR** ($CDCl_3$, 400 MHz): δ (ppm) 6.01 (s, 2H, C(6)–H and C(7)–H), 3.67 (rot. s, 2H; 1x C(2)–H_2, 1x C(4)–H_2), 3.30 (d, J = 6.0 Hz, 2H; C(1)–H, C(5)–H), 2.89 (dd, J = 12.0, 6.5 Hz, 2H, 1x C(2)–H_2, 1x C(4)–H_2), 1.45 (s, 9H; $C(CH_3)_3$).

^{13}**C NMR** ($CDCl_3$, 101 MHz): δ (ppm) 156.6 (C=O), 139.2, 138.7 (rot.; C(6), C(7)), 79.2 (C($CH_3)_3$), 44.9, 46.3 (rot.; C(1), C(2), C(4), C(5)), 28.7 (C($CH_3)_3$).

Synthesis of 2f

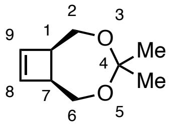

A mixture of *cis*-cyclobut-3-ene-1,2-diyl)dimethanol (456 mg, 4.0 mmol) and *p*-toluenesulfonic acid (15.2 mg, 2.0 mol%) was dissolved in CH_2Cl_2 (15 mL) under an argon atmosphere. 2-Methoxypropene (0.77 mL, 8.0 mmol) The mixture was stirred at room temperature (23 °C) for 24 h. Purification by automated medium-pressure chromatography (pentane/Et_2O = 100/0 to 70/30) afforded the product **2f** as a yellow oil (348 mg, 56% yield).

^{1}H NMR ($CDCl_3$, 400 MHz): δ (ppm) 6.12 (s, 2H; C(8)–H, C(9)–H), 3.99 – 3.82 (m, 1H; 1x C(2)–H_2, 1x C(6)–H_2), 3.81 – 3.70 (m, 2H; 1x C(2)–H_2, 1x C(6)–H_2), 3.20 – 3.10 (m, 2H; C(1)–H, C(7)–H), 1.40 (s, 3H; CH_3), 1.39 (s, 3H; CH_3).

^{13}C NMR ($CDCl_3$, 101 MHz): δ (ppm) 138.1 (C(8), C(9)), 102.1 (C(4)), 63.3 (C(2), C(6)), 47.7 (C(1), C(7)), 25.0 (rot.; CH_3), 24.5 (rot.; CH_3).

IR (neat): 2992 (w), 2935 (w), 1381 (w), 1368 (w), 1215 (m), 1151 (m), 1075 (s), 1036 (m), 993 (m), 829 (w), 797 (m), 737 (m), 710 (s) cm^{-1}.

HRMS (ESI/APCI): m/z not found.

Note: We were not able to detect the molecular ion or a characteristic fragment for this molecule.

1.3. Hydroacylation reactions

All reactions were carried out under an inert argon atmosphere using standard Schlenk techniques with all reagents weighed open to air.

General procedure A: Asymmetric Rh-catalyzed acylation

Flask I: A solution of cyclobutene (0.6 mmol) and salicylaldehyde (0.4 mol) in dry toluene (0.7 mL) was prepared under an argon atmosphere.

Flask II: A resulting (slightly turbid) solution of $[Rh(cod)OH]_2$ (4.6 mg, 2.5 mol%) and (*R*,*R*)-MeDuphos (7.3 mg, 6.0 mol%) was stirred in dry toluene (1.0 mL) under an argon atmosphere at room temperature (23 °C) for 15 min. Then, the solution of *Flask I* was added to *Flask II*, and *Flask I* was rinsed with dry toluene (0.3 mL). The reaction mixture was stirred 60 °C for 20 h. The reaction mixture was then concentrated *in vacuo* and purified by automated liquid chromatography.

General procedure B: Regioselective Rh-catalyzed acylation

Flask I: A solution of cyclobutene (0.6 mmol) and salicylaldehyde (0.4 mol) in dry toluene (0.7 mL) was prepared under an argon atmosphere.

Flask II: A resulting (clear) solution of $[Rh(cod)OH]_2$ (5.5 mg, 3.0 mol%) and dppf (13.3 mg, 6.0 mol%) was stirred in dry toluene (1.0 mL) under an argon atmosphere at room temperature (23 °C) for 15 min. Then, the solution of *Flask I* was added to *Flask II*, and *Flask I* was rinsed with dry toluene (0.3 mL). The reaction mixture was stirred 60 °C for 20 h. The reaction mixture was then concentrated *in vacuo* and purified by automated liquid chromatography.

Racemates

Racemic samples were synthesized with (±)-BINAP or dppf.

Epimerization of (±)-3a

A mixture of (±)-**3a** (0.5 mg) and a tip of a spatula of K_2CO_3 in MeOH (0.5 mL) was stirred for 16 h at room temperature (23 °C). The mixture was filtered and analyzed by super critical fluid chromatography.

SFC Chiralpak® IF 1500 psi, 30 °C; flow: 1.5 mL/min; from 1% to 30% MeOH in 5 min, then from 30% to 50% MeOH in 0.5 min, then hold 50% MeOH for 1.5 min; (minor diastereomer t_R = 5.97 min; major diastereomer t_R = 6.07 min, minor diastereomer t_R = 6.21 min; major diastereomer t_R = 6.49 min).

Synthesis of 3a

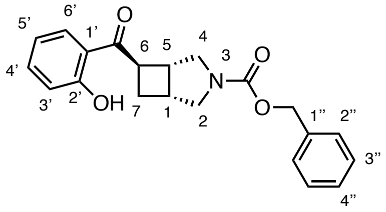

The corresponding compound was prepared following general procedure A using salicylaldehyde and cyclobutene **2**. The mixture was stirred at 60 °C for 1 h. Purification by automated medium-pressure chromatography (hexane/EtOAc = 100/0 to 70/30) afforded the product **3a** as a colorless solid (113 mg, 81% yield). SFC analysis showed an enantiomeric excess of 98%.

^{1}H NMR ($CDCl_3$, 400 MHz): δ (ppm) 12.26 (s, 1H; OH), 7.48 – 7.28 (m, 7H; 2x C(2'')–H, 2x C(3'')–H, C(4'')–H, C(6')–H, C(4')–H), 6.99 (dd, *J* = 8.6, 1.2 Hz, 1H; C(3')–H), 6.87 (td, *J* = 7.6, 1.2 Hz, 1H; C(5')–H), 5.21 (s, 2H; CH_2Ph), 4.07 – 3.67 (m, 3H; 2x CH_2N, C(6)–H), 3.45 – 3.31 (m, 3H; 2x CH_2N, C(5)–H), 2.96 – 2.84 (m, 1H; C(1)–H), 2.58 (br. s, 1H; C(7)–H), 2.11 (br. s, 1H; C(7)–H).

^{13}C NMR ($CDCl_3$, 101 MHz): δ (ppm) 206.0 (C=O), 163.0 (C(2')), 155.7 (CO_2N), 136.9 (C(1'')), 136.5 (C(4')), 129.8 (C(6')), 128.7 (2x C(3'')), 128.2 (C(4'')), 128.1 (2x C(2'')), 119.1 (C(5')), 118.8 (C(3')), 117.8 (C(1')), 67.2 (CH_2Ph), 52.7, 52.3 (rot.; 2x CH_2N), 44.3 (C(6)), 39.6 (rot.; C(5)), 34.8, 33.9 (rot.; 2x C(1)), 29.2, 28.4 (rot.; 2x C(7)).

IR ($CHCl_3$ film): 2980 (w), 2872 (w), 1702 (s), 1634 (m), 1486 (w), 1447 (m), 1416 (m), 1358 (m), 1295 (w), 1229 (m), 1209 (m), 1158 (m), 1098 (m), 757 (m), 698 (w) cm^{-1}.

HRMS (ESI): m/z calcd for $C_{21}H_{21}O_4NNa^+$ $[M + Na]^+$ 374.1363 found 374.1362.

SFC Chiralpak® IF 1500 psi, 30 °C; flow: 1.5 mL/min; from 1% to 30% MeOH in 5 min, then from 30% to 50% MeOH in 0.5 min, then hold 50% MeOH for 1.5 min; 99:1 e.r. (minor enantiomer t_R = 6.10 min; major enantiomer t_R = 6.46 min).

$[\alpha]^{25}_D$ = –109.6 (*c* = 1.0, $CHCl_3$).

m.p.: 60 – 62 °C.

Synthesis of 3b

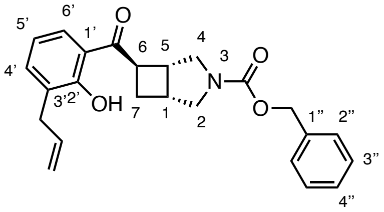

The corresponding compound was prepared following general procedure A using 3-allylsalicylaldehyde and cyclobutene **2**. The mixture was stirred at 60 °C for 3 h. Purification by automated medium-pressure chromatography (hexane/EtOAc = 100/0 to 70/30) afforded the product **3b** as a pale-yellow oil (117 mg, 75% yield). SFC analysis showed an enantiomeric excess of 97%.

^{1}H NMR ($CDCl_3$, 400 MHz): δ (ppm) 12.61 (s, 1H; OH), 7.45 – 7.30 (m, 7H; 2x C(2'')–H, 2x C(3'')–H, C(4'')–H, C(6')–H, C(4')–H), 6.82 (app. t, *J* = 7.7 Hz, 1H; C(5')–H), 6.07 – 5.94 (m, 1H; C*H*=CH_2), 5.21 (s, 2H; CH_2Ph), 5.10 (dq, *J* = 7.2, 1.5 Hz, 1H; CH=CH_2), 5.07 (app. t, *J* = 1.5 Hz, 1H; CH=CH_2), 4.05 – 3.64 (m, 3H; 2x CH_2N, C(6)–H), 3.50 – 3.29 (m, 5H; 2x CH_2N, C(5)–H, CH_2CH=CH_2), 2.97 – 2.87 (m, 1H; C(1)–H), 2.59 (br. s, 1H; C(7)–H), 2.10 (br. s, 1H; C(7)–H).

^{13}C NMR ($CDCl_3$, 101 MHz): δ (ppm) 206.2 (C=O), 161.0 (C(2')), 155.7 (CO_2N), 137.0 (C(1'')), 136.5 (C(4')), 136.2 (CH_2*C*H=CH_2), 129.8 (C(3')), 128.6 (2x C(3'')), 128.2 (C(4'')), 128.1 (2x C(2'')), 127.9 (C(6')), 118.6 (C(5')), 117.4 (C(1')), 116.2 (CH_2CH=*C*H_2), 67.1 (CH_2Ph), 52.8, 52.2 (rot.; 2x CH_2N), 44.4 (C(6)), 39.7 (rot.; C(5)), 34.7, 33.9 (rot.; 2x C(1)), 33.6 (*C*H_2CH=CH_2), 29.2, 28.4 (rot.; 2x C(7)).

IR ($CHCl_3$ film): 2973 (w), 2871 (w), 1703 (s), 1628 (m), 1498 (m), 1426 (m), 1358 (m), 1295 (w), 1230 (m), 1158 (w), 1098 (m), 1028 (w), 915 (w), 769 (w), 753 (m), 698 (w) cm^{-1}.

HRMS (ESI): m/z calcd for $C_{24}H_{26}O_4N^+$ $[M + H]^+$ 392.1856 found 392.1857.

SFC Chiralpak® IB 1500 psi, 30 °C; flow: 1.5 mL/min; from 1% to 30% MeOH in 5 min; 98.5:1.5 e.r. (major enantiomer t_R = 3.92 min; minor enantiomer t_R = 4.07 min).

$[\alpha]^{25}_D$ = –90.2 (*c* = 1.0, $CHCl_3$).

Synthesis of 3c

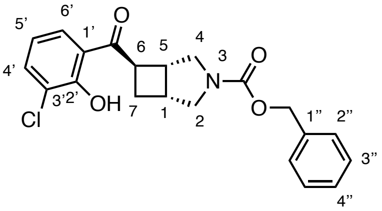

The corresponding compound was prepared following general procedure A using 3-chlorosalicylaldehyde and cyclobutene **2** with an increased catalyst loading of [Rh(cod)OH]$_2$ (5.0 mol%) and (*R*,*R*)-MeDuphos (12 mol%). The mixture was stirred at 60 °C for 4 h. Purification by automated medium-pressure chromatography (hexane/EtOAc = 100/0 to 70/30) afforded the product **3c** as a colorless solid (103 mg, 67 % yield). SFC analysis showed an enantiomeric excess of 99%.

^{1}H NMR ($CDCl_3$, 400 MHz): δ (ppm) 12.85 (s, 1H; OH), 7.56 (dd, *J* = 7.9, 1.5 Hz, 1H; C(6')–H), 7.47 – 7.29 (m, 6H; 2x C(2'')–H, 2x C(3'')–H, C(4'')–H, C(4')–H), 6.83 (app. t, *J* = 7.9 Hz, 1H; C(5')–H), 5.21 (s, 2H; CH_2Ph), 4.05 – 3.62 (m, 3H; 2x CH_2N, C(6)–H), 3.48 – 3.26 (m, 3H; 2x CH_2N, C(5)–H), 2.97 – 2.85 (m, 1H; C(1)–H), 2.58 (br. s, 1H; C(7)–H), 2.11 (br. s, 1H; C(7)–H).

^{13}C NMR ($CDCl_3$, 101 MHz): δ (ppm) 205.9 (C=O), 158.7 (C(2')), 155.7 (CO_2N), 136.9 (C(1'')), 136.4 (C(6')), 128.6 (2x C(3'')), 128.22, 128.19 (C(4''), C(4')), 128.1 (2x C(2'')), 123.3 (C(3')), 119.2 (C(5')), 118.8 (C(1')), 67.2 (CH_2Ph), 52.8, 52.2 (rot.; 2x CH_2N), 44.4 (C(6)), 39.6 (rot.; C(5)), 34.9, 33.9 (rot.; 2x C(1)), 29.1, 28.4 (rot.; 2x C(7)).

IR ($CHCl_3$ film): 2980 (w), 2872 (w), 1702 (s), 1637 (m), 1433 (s), 1359 (m), 1229 (m), 1150 (m), 1099 (w), 743 (w) cm^{-1}.

HRMS (ESI): m/z calcd for $C_{21}H_{19}O_4N^{35}Cl^{+}$ [M – H]$^{-}$ 384.1008 found 384.1001.

SFC Chiralpak® IB 1500 psi, 30 °C; flow: 1.5 mL/min; from 1% to 30% MeOH in 5 min, then from 30% to 50% MeOH in 0.5 min; 99.5:0.5 e.r. (major enantiomer t_R = 4.73 min; minor enantiomer t_R = 5.13 min).

$[\alpha]^{25}_{D}$ = –115.5 (*c* = 1.0, $CHCl_3$).

m.p.: 112 – 113 °C.

Synthesis of 3d

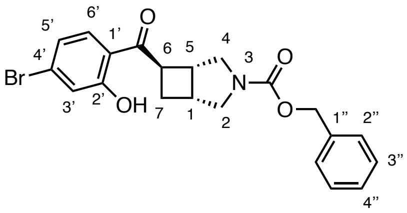

The corresponding compound was prepared following general procedure A using 4-bromosalicylaldehyde and cyclobutene **2** with an increased catalyst loading of $[Rh(cod)OH]_2$ (5.0 mol%) and (*R*,*R*)-MeDuphos (12 mol%). The mixture was stirred at 60 °C for 4 h. Purification by automated medium-pressure chromatography (hexane/EtOAc = 100/0 to 70/30) afforded the product **3d** as a yellow oil (124 mg, 71 % yield). SFC analysis showed an enantiomeric excess of 97%.

^{1}H NMR ($CDCl_3$, 400 MHz): δ (ppm) 12.35 (s, 1H; OH), 7.47 – 7.29 (m, 6H; 2x C(2'')–H, 2x C(3'')–H, C(4'')–H, C(6')–H), 7.19 (d, *J* = 1.9 Hz, 1H; C(3')–H), 7.00 (dd, *J* = 8.5, 2.0 Hz, 1H; C(6')–H), 5.21 (s, 2H; CH_2Ph), 4.00 – 3.58 (br. m, 3H; 2x CH_2N, C(6)–H), 3.50 – 3.27 (m, 3H; 2x CH_2N, C(5)–H), 2.92 (dd, *J* = 8.6, 4.8 Hz, 1H; C(1)–H), 2.55 (br. s, 1H; C(7)–H), 2.10 (br. s, 1H; C(7)–H).

^{13}C NMR ($CDCl_3$, 101 MHz): δ (ppm) 205.5 (C=O), 163.4 (C(2')), 155.7 (CO_2N), 136.9 (C(1'')), 130.9 (C(4')), 130.7 (C(6')), 128.7 (2x C(3'')), 128.2 (C(4')), 128.1 (2x C(2'')), 122.7 (C(3')), 122.0 (C(5')), 116.7 (C(1')), 67.2 (CH_2Ph), 52.7, 52.1 (rot.; 2x CH_2N), 44.3 (C(6)), 39.3 (rot.; C(5)), 34.8, 33.4 (rot.; 2x C(1)), 28.8, 28.3 (rot.; 2x C(7)).

IR ($CHCl_3$ film): 2981 (w), 1699 (m), 1634 (m), 1416 (w), 1359 (w), 1229 (w), 1099 (w), 901 (w), 754 (s), 698 (w) cm^{-1}.

HRMS (ESI): m/z calcd for $C_{21}H_{19}O_4N^{79}Br^+$ $[M – H]^–$ 428.0503 found 428.0499.

SFC Chiralpak® IB 1500 psi, 30 °C; flow: 1.5 mL/min; hold 1% MeOH for 2 min, then from 1% to 2.5% MeOH in 1 min, then hold 2.5% MeOH for 5 min, then from 2.5% to 35% MeOH in 7 min; 98.5:1.5 e.r. (major enantiomer t_R = 12.34 min; minor enantiomer t_R = 12.56 min).

$[\alpha]^{25}_D$ = –96.3 (*c* = 1.0, $CHCl_3$).

Synthesis of 3e

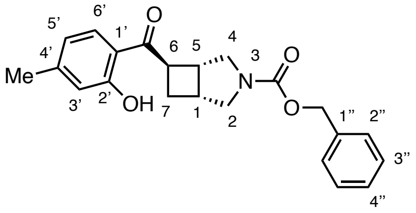

The corresponding compound was prepared following general procedure A using 4-methylsalicylaldehyde and cyclobutene **2**. The mixture was stirred at 60 °C for 3 h. Purification by automated medium-pressure chromatography (hexane/EtOAc = 100/0 to 70/30) afforded the product **3e** as a yellow oil (121 mg, 83% yield). SFC analysis showed an enantiomeric excess of 96%.

^{1}H NMR ($CDCl_3$, 400 MHz): δ (ppm) 12.29 (s, 1H; OH), 7.48 – 7.28 (m, 6H; 2x C(2'')–H, 2x C(3'')–H, C(4'')–H, C(6')–H), 6.80 (dd, *J* = 1.8, 0.9 Hz, 1H; C(3')–H), 6.67 (dd, *J* = 8.2, 1.7 Hz, 1H; C(5')–H), 5.21 (s, 2H; CH_2Ph), 4.03 – 3.64 (br. m, 3H; 2x CH_2N, C(6)–H), 3.45 – 3.25 (m, 3H; 2x CH_2N, C(5)–H), 2.91 (dq, *J* = 11.5, 6.8 Hz, 1H; C(1)–H), 2.57 (br. s, 1H; C(7)–H), 2.34 (s, 3H; CH_3), 2.08 (br. s, 1H; C(7)–H).

^{13}C NMR ($CDCl_3$, 101 MHz): δ (ppm) 205.3 (C=O), 163.1 (C(2')), 155.7 (CO_2N), 148.1 (C(4')), 136.9 (C(1'')), 129.7 (C(6')), 128.6 (2x C(3'')), 128.2 (C(4'')), 128.1 (2x C(2'')), 120.4 (C(5')), 118.8 (C(3')), 115.6 (C(1')), 67.1 (CH_2Ph), 52.7, 52.2 (rot.; 2x CH_2N), 44.1 (C(6)), 39.7 (rot.; C(5)), 33.5 (rot.; C(1)), 29.2, 28.3 (rot.; 2x C(7)), 22.1 (CH_3).

IR ($CHCl_3$ film): 2980 (w), 1702 (s), 1635 (m), 1415 (m), 1229 (m), 1211 (m), 1098 (w), 770 (w), 698 (w) cm^{-1}.

HRMS (ESI): m/z calcd for $C_{22}H_{24}O_4N^+$ $[M + H]^+$ 366.1700 found 366.1700.

SFC Chiralpak® IB 1500 psi, 30 °C; flow: 1.5 mL/min; hold 1% MeOH for 2 min, then from 1% to 2.5% MeOH in 1 min, then hold 2.5% MeOH for 5 min, then from 2.5% to 35% MeOH in 7 min; 98:2 e.r. (major enantiomer t_R = 10.88 min; minor enantiomer t_R = 11.04 min).

$[\alpha]^{25}_D$ = –109.4 (*c* = 1.0, $CHCl_3$).

Synthesis of 3f

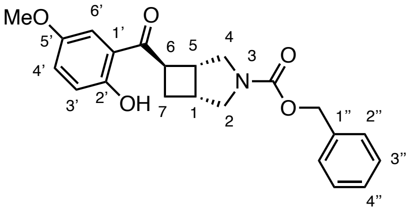

The corresponding compound was prepared following general procedure A using 5-methoxysalicylaldehyde and cyclobutene **2**. The mixture was stirred at 60 °C for 2 h. Purification by automated medium-pressure chromatography (hexane/EtOAc = 100/0 to 50/50) afforded the product **3f** as a yellow oil (121 mg, 79% yield). SFC analysis showed an enantiomeric excess of 98%.

^{1}H NMR ($CDCl_3$, 400 MHz): δ (ppm) 11.86 (s, 1H; OH), 7.44 – 7.29 (m, 5H; 2x C(2'')–H, 2x C(3'')–H, C(4'')–H), 7.10 (dd, *J* = 9.0, 3.1 Hz, 1H; C(4')–H), 6.94 (d, *J* = 9.1 Hz, 1H; C(3')–H), 6.90 (br. s, 1H; C(6')–H), 5.21 (s, 2H; CH_2Ph), 4.07 – 3.55 (br. m, 6H; 2x CH_2N, C(6)–H, OCH_3), 3.46 – 3.25 (m, 3H; 2x CH_2N, C(5)–H), 2.91 (p, *J* = 6.6 Hz, 1H; C(1)–H), 2.59 (dt, *J* = 12.4, 8.2 Hz, 1H; C(7)–H), 2.12 (br. s, 1H; C(7)–H).

^{13}C NMR ($CDCl_3$, 101 MHz): δ (ppm) 205.4 (C=O), 157.3 (C(2')), 155.7 (CO_2N), 151.9 (C(5')), 136.9 (C(1'')), 128.6 (2x C(3'')), 128.2 (C(4'')), 128.1 (2x C(2'')), 124.4 (C(4')), 119.7 (C(3')), 117.2 (C(1')), 112.3 (C(6')), 67.2 (CH_2Ph), 56.1 (OCH_3), 52.8, 52.3 (rot.; 2x CH_2N), 44.4 (C(6)), 39.8, 39.4 (rot.; 2x C(5)), 34.8, 33.9 (rot.; 2x C(1)), 28.9, 28.3 (rot.; 2x C(7)).

IR ($CHCl_3$ film): 2980 (w), 1702 (s), 1640 (w), 1614 (w), 1486 (s), 1417 (m), 1359 (m), 1285 (m), 1229 (m), 1176 (m), 1098 (w), 770 (w), 698 (w) cm^{-1}.

HRMS (ESI): m/z calcd for $C_{22}H_{23}O_5NNa^+$ $[M + Na]^+$ 404.1468 found 404.1473.

SFC Chiralpak® IB 1500 psi, 30 °C; flow: 1.5 mL/min; from 1% to 30% MeOH in 8 min; 99:1 e.r. (minor enantiomer t_R = 7.70 min; major enantiomer t_R = 7.82 min).

$[\alpha]^{25}_D$ = –93.8 (*c* = 1.0, $CHCl_3$).

Synthesis of 3g

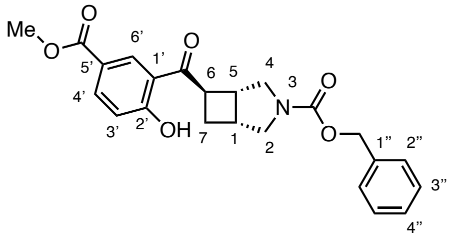

The corresponding compound was prepared following general procedure A using 5-methoxycarbonylsalicylaldehyde and cyclobutene **2** with an increased catalyst loading of $[Rh(cod)OH]_2$ (5.0 mol%) and (*R*,*R*)-MeDuphos (12 mol%). The mixture was stirred at 60 °C for 4 h. Purification by automated medium-pressure chromatography (hexane/EtOAc = 100/0 to 70/30) afforded the product **3g** as a colorless oil (118 mg, 72% yield). SFC analysis showed an enantiomeric excess of 98%.

^{1}H NMR ($CDCl_3$, 400 MHz): δ (ppm) 12.66 (s, 1H; OH), 8.22 (d, *J* = 2.1 Hz, 1H; C(6')–H), 8.12 (dd, *J* = 8.8, 2.1 Hz, 1H; C(4')–H), 7.46 – 7.29 (m, 5H; 2x C(2'')–H, 2x C(3'')–H, C(4'')–H), 7.03 (d, *J* = 8.8 Hz, 1H; C(3')–H), 5.22 (s, 2H; CH_2Ph), 4.02 – 3.72 (m, 6H; 2x CH_2N, C(6)–H, OCH_3), 3.48 – 3.30 (m, 3H; 2x CH_2N, C(5)–H), 2.94 (tt, *J* = 11.4, 5.6 Hz, 1H; C(1)–H), 2.55 (dt, *J* = 12.4, 8.2 Hz, 1H; C(7)–H), 2.21 (ddd, *J* = 12.9, 10.0, 4.2 Hz, 1H; C(7)–H).

^{13}C NMR ($CDCl_3$, 101 MHz): δ (ppm) 206.11 (C=O), 166.5 (C(2')), 165.9 (CO_2CH_3), 155.7 (CO_2N), 137.2 (C(4')), 136.9 (C(1'')), 132.2 (C(6')), 128.6 (2x C(3'')), 128.2 (C(4'')), 128.1 (2x C(2'')), 121.3 (C(5')), 119.0 (C(3')), 117.2 (C(1')), 67.2 (CH_2Ph), 52.3 (2x CH_2N, OCH_3), 44.4 (C(6)), 39.7 (rot.; C(5)), 34.7 (rot.; C(1)), 29.1 (C(7)).

IR ($CHCl_3$ film): 2953 (w), 1706 (s), 1638 (m), 1419 (m), 1358 (m), 1278 (s), 1227 (s), 1108 (m), 769 (w), 698 (w) cm^{-1}.

HRMS (ESI): m/z calcd for $C_{23}H_{24}O_6N^+$ $[M + H]^+$ 410.1598 found 410.1579.

SFC Chiralpak® IE 1500 psi, 30 °C; flow: 1.5 mL/min; from 1% to 30% MeOH in 5 min, then from 30% to 50% MeOH in 0.5 min, then hold 50% MeOH for 1.5 min; 99:1 e.r. (major enantiomer t_R = 6.60 min; minor enantiomer t_R = 7.03 min).

$[\alpha]^{25}_D$ = –81.3 (*c* = 1.0, $CHCl_3$).

Synthesis of 3h

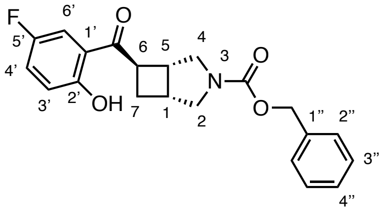

The corresponding compound was prepared following general procedure A using 5-fluorosalicylaldehyde and cyclobutene **2** with an increased catalyst loading of $[Rh(cod)OH]_2$ (5.0 mol%) and (*R*,*R*)-MeDuphos (12 mol%). The mixture was stirred at 60 °C for 2 h. Purification by automated medium-pressure chromatography (hexane/EtOAc = 100/0 to 70/30) afforded the product **3h** as a colorless oil (105 mg, 71% yield). SFC analysis showed an enantiomeric excess of 97%.

^{1}H NMR ($CDCl_3$, 400 MHz): δ (ppm) 11.98 (s, 1H; OH), 7.47 – 7.29 (m, 5H; 2x C(2'')–H, 2x C(3'')–H, C(4'')–H), 7.20 (ddd, *J* = 9.1, 7.7, 3.1 Hz, 1H; C(4')–H), 7.12 (dd, *J* = 8.8, 3.1 Hz, 1H; C(6')–H), 6.96 (dd, *J* = 9.1, 4.5 Hz, 1H; C(3')–H), 5.21 (s, 2H; CH_2Ph), 3.97 – 3.72 (m, 2H; 2x CH_2N), 3.64 (t, *J* = 7.9 Hz, 1H; C(6)–H), 3.54 – 3.27 (m, 3H; 2x CH_2N, C(5)–H), 2.98 – 2.87 (m, 1H; C(1)–H), 2.59 – 2.48 (m, 1H; C(7)–H), 2.14 (br. s, 1H; C(7)–H).

^{13}C NMR ($CDCl_3$, 101 MHz): δ (ppm) 205.1 (d, *J* = 2.6 Hz; C=O), 159.16 (d, *J* = 1.5 Hz; C(2')), 155.7 (CO_2N), 154.93 (d, *J* = 239.1 Hz; C(5')), 128.6 (2x C(3'')), 128.2 (C(4'')), 128.1 (2x C(2'')), 124.1 (d, *J* = 23.6 Hz; C(4')), 120.1 (d, *J* = 7.2 Hz; C(3')), 117.3 (d, *J* = 6.0 Hz; C(1')), 114.6 (d, *J* = 23.0 Hz; C(6')), 67.2 (CH_2Ph), 52.7, 52.1 (rot.; 2x CH_2N), 44.4, 39.4 (C(6)), 38.9 (rot.; 2x C(5)), 34.9, 34.0 (rot.; 2x C(1)), 29.0 (rot.; C(7)).

^{19}F NMR ($CDCl_3$, 376 MHz): δ (ppm) -123.59 (app. t, *J* = 9.9 Hz).

IR ($CHCl_3$ film): 2960 (w), 2872 (w), 1702 (s), 1643 (w), 1482 (m), 1418 (m), 1359 (m), 1283 (w), 1246 (m), 1229 (w), 1172 (m), 1099 (w), 830 (w), 788 (w), 769 (w), 698 (w) cm^{-1}.

HRMS (ESI): m/z calcd for $C_{21}H_{20}O_4FNNa^+$ $[M + Na]^+$ 392.1269 found 392.1268.

SFC Chiralpak® IF 1500 psi, 30 °C; flow: 1.5 mL/min; from 1% to 30% MeOH in 5 min, then from 30% to 50% MeOH in 0.5 min, then hold 50% MeOH for 1.5 min; 98.5:1.5 e.r. (minor enantiomer t_R = 5.56 min; major enantiomer t_R = 6.23 min).

$[\alpha]^{25}_{D}$ = –109.3 (*c* = 1.0, $CHCl_3$).

Synthesis of 3j

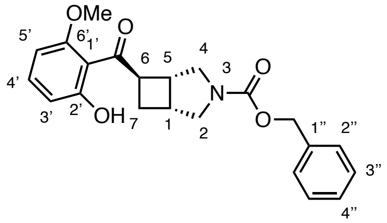

The corresponding compound was prepared following general procedure A using 6-methoxysalicylaldehyde and cyclobutene **2**. The mixture was stirred at 60 °C for 2 h. Purification by automated medium-pressure chromatography (hexane/EtOAc = 100/0 to 60/40) afforded the product **3j** as a pale-yellow oil (119 mg, 78% yield). SFC analysis showed an enantiomeric excess of 98%.

^{1}H NMR ($CDCl_3$, 400 MHz): δ (ppm) 13.07 (s, 1H; OH), 7.45 – 7.28 (m, 6H; 2x C(2'')–H, 2x C(3'')–H, C(4'')–H, C(4')–H), 6.58 (dd, *J* = 8.4, 1.0 Hz, 1H; C(3')–H), 6.35 (d, *J* = 8.3 Hz, 1H; C(5')–H), 5.22 (d, *J* = 12.7 Hz, 1H; 1x CH_2Ph), 5.19 (d, *J* = 12.7 Hz, 1H; 1x CH_2Ph), 4.11 – 3.62 (br. m, 6H; 2x CH_2N, C(6)–H, OCH_3), 3.52 – 3.10 (m, 3H; 2x CH_2N, C(5)–H), 2.86 (s, 1H; C(1)–H), 2.57 (br. s, 1H; C(7)–H), 2.02 (br. s, 1H; C(7)–H).

^{13}C NMR ($CDCl_3$, 101 MHz): δ (ppm) 206.7 (C=O), 165.0 (C(2')), 161.0 (C(6')), 155.6 (CO_2N), 137.0 (C(1'')), 136.2 (C(4')), 128.6 (2x C(3'')), 128.1 (C(4'')), 128.0 (2x C(2'')), 111.0 (C(3')), 110.2 (C(1')), 101.3 (C(5')), 67.0 (CH_2Ph), 55.8 (OCH_3), 53.1, 52.6, 52.2 (rot.; 3x CH_2N), 49.6 (C(6)), 40.3 (C(5)), 34.6, 33.4 (rot.; 2x C(1)), 28.7, 27.9 (rot.; 2x C(7)).

IR ($CHCl_3$ film): 2942 (w), 2869 (w), 1700 (s), 1623 (m), 1594 (m), 1456 (m), 1437 (m), 1419 (m), 1359 (m), 1236 (s), 1184 (w), 1093 (s), 746 (w), 699 (w) cm^{-1}.

HRMS (ESI): m/z calcd for $C_{22}H_{23}O_5NNa^+$ $[M + Na]^+$ 404.1468 found 404.1473.

SFC Chiralpak® IA 1500 psi, 30 °C; flow: 1.5 mL/min; from 1% to 30% MeOH in 5 min; 99:1 e.r. (major enantiomer t_R = 4.71 min; minor enantiomer t_R = 4.98 min).

$[\alpha]^{25}_D$ = –87.8 (*c* = 1.0, $CHCl_3$).

Synthesis of 4a

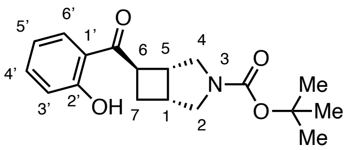

The corresponding compound was prepared following general procedure A using salicylaldehyde and cyclobutene **2**. The mixture was stirred at 60 °C for 1 h. Purification by automated medium-pressure chromatography (hexane/EtOAc = 100/0 to 80/20) afforded the product **4a** as a colorless oil (98 mg, 76% yield). SFC analysis showed an enantiomeric excess of 98%.

^{1}H NMR ($CDCl_3$, 400 MHz): δ (ppm) 12.28 (s, 1H; OH), 7.51 (dd, *J* = 8.0, 1.7 Hz, 1H; C(6')–H), 7.46 (ddd, *J* = 8.7, 7.2, 1.7 Hz, 1H; C(4')–H), 6.99 (dd, *J* = 8.4, 1.2 Hz, 1H; C(3')–H), 6.87 (ddd, *J* = 8.2, 7.2, 1.2 Hz, 1H; C(5')–H), 3.83 – 3.72 (m, 2H; 1x CH_2N, C(6)–H), 3.70 – 3.55 (m, 1H; 1x CH_2N), 3.39 – 3.24 (m, 3H; 2x CH_2N, C(5)–H), 2.89 (dddd, *J* = 11.7, 8.9, 6.6, 3.7 Hz, 1H; C(1)–H), 2.58 (dt, *J* = 12.5, 8.2 Hz, 1H; C(7)–H), 2.12 (ddd, *J* = 13.3, 10.0, 4.4 Hz, 1H; C(7)–H), 1.51 (s, 9H; $C(CH_3)_3$)

^{13}C NMR ($CDCl_3$, 101 MHz): δ (ppm) 206.2 (C=O), 163.0 (C(2')), 155.5 (CO_2N), 136.4 (C(4')), 129.8 (C(6')), 119.1 (C(5')), 118.8 (C(3')), 117.9 (C(1')), 79.8 (*C*(CH_3)$_3$) , 52.7 (CH_2N), 52.1 (CH_2N), 44.4 (C(6)), 39.7 (C(5)), 34.4 (C(1)), 28.7 (C(*C*H_3)$_3$, C(7)).

IR ($CHCl_3$ film): 2980 (m), 2882 (w), 1695 (s), 1635 (m), 1486 (w), 1448 (w), 1365 (s), 1349 (m), 1295 (w), 1238 (m), 1158 (s), 1100 (w), 758 (w) cm^{-1}.

HRMS (ESI): m/z calcd for $C_{18}H_{23}O_4NNa^+$ $[M + Na]^+$ 340.1519 found 340.1519.

SFC Chiralpak® IB 1500 psi, 30 °C; flow: 1.5 mL/min; from 1% to 30% MeOH in 5 min, then from 30% to 50% MeOH in 0.5 min; 99:1 e.r. (major enantiomer t_R = 2.23 min; minor enantiomer t_R = 2.38 min).

$[\alpha]^{25}_D$ = –119.1 (*c* = 1.0, $CHCl_3$).

Synthesis of 4b

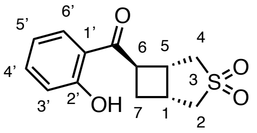

The corresponding compound was prepared following general procedure A using salicylaldehyde and cyclobutene **2b**. The mixture was stirred at 60 °C for 2 h. ^{1}H NMR analysis of the unpurified reaction mixture showed a diastereomeric ratio of 8:1. Purification by automated medium-pressure chromatography (hexane/EtOAc = 100/0 to 40/60) afforded the product **4b** as a colorless solid (89 mg, 84% yield). SFC analysis showed an enantiomeric excess of 96%.

^{1}H NMR ($CDCl_3$, 400 MHz): δ (ppm) 12.08 (s, 1H; OH), 7.53 – 7.42 (m, 2H; C(6')–H, C(4')–H), 7.10 – 6.96 (m, 1H; C(3')–H), 6.96 – 6.74 (m, 1H; C(5')–H), 4.37 – 4.24 (m, 1H; C(6)–H), 3.87 (tdd, *J* = 8.4, 6.2, 2.1 Hz, 1H; C(5)–H), 3.38 – 3.18 (m, 3H; 2x CH_2SO_2, C(1)–H), 3.17 – 3.01 (m, 2H; 2x CH_2SO_2), 2.72 – 2.53 (m, 2H; C(7)–H_2).

^{13}C NMR ($CDCl_3$, 101 MHz): δ (ppm) 204.7 (C=O), 163.0 (C(2')), 136.9 (C(6')), 129.8 (C(5')), 119.3 (C(5')), 118.9 (C(3')), 117.5 (C(1')), 55.4 (CH_2SO_2), 55.0 (CH_2SO_2), 43.6 (C(6)), 33.3 (C(5)), 30.2 (C(1)), 29.6 (C(7)).

IR ($CHCl_3$ film): 2982 (w), 1634 (s), 1447 (m), 1415 (w), 1304 (s), 1273 (s), 1211 (m), 1140 (s), 1082 (w), 874 (w), 755 (s), 672 (m) cm^{-1}.

HRMS (ESI): m/z calcd for $C_{13}H_{13}O_4S^+$ $[M – H]^-$ 265.0540 found 265.0537.

SFC Chiralpak® IB 1500 psi, 30 °C; flow: 1.5 mL/min; from 1% to 30% MeOH in 5 min; 98:2 e.r. (major enantiomer t_R = 3.67 min; minor enantiomer t_R = 4.12 min).

$[\alpha]^{25}_D$ = –138.2 (*c* = 1.0, $CHCl_3$).

m.p.: 172 – 174°C.

Synthesis of 4c

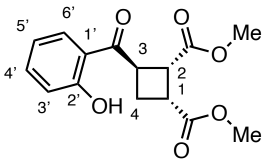

The corresponding compound was prepared following general procedure A using salicylaldehyde and cyclobutene **2c**. The mixture was stirred at 60 °C for 2 h. ^{1}H NMR analysis of the unpurified reaction mixture showed a diastereomeric ratio of >20:1:1. Purification by automated medium-pressure chromatography (hexane/EtOAc = 100/0 to 80/20) afforded a mixture of **2c** and **4c**. **2c** was removed under reduced pressure (approx. 0.1 mbar) at 50 °C and a colorless solid (101 mg, 84% yield) contaminated with 3% unreacted **2c** was obtained. SFC analysis showed an enantiomeric excess of 96%.

^{1}H NMR ($CDCl_3$, 400 MHz): δ (ppm) 12.08 (s, 1H; OH), 7.69 (dd, *J* = 8.0, 1.6 Hz, 1H; C(6')–H), 7.48 (ddd, *J* = 8.7, 7.2, 1.7 Hz, 1H; C(4')–H), 6.99 (dd, *J* = 8.4, 1.2 Hz, 1H; C(3')–H), 6.91 (ddd, *J* = 8.2, 7.2, 1.2 Hz, 1H; C(5')–H), 4.63 – 4.55 (m, 1H; C(3)–H), 3.98 (ddd, *J* = 9.5, 8.3, 1.0 Hz, 1H; C(2)–H), 3.75 (s, 3H; OCH_3), 3.70 (s, 3H; OCH_3), 3.41 (dddd, *J* = 10.1, 9.2, 4.1, 1.2 Hz, 1H; C(1)–H), 2.70 – 2.60 (m, 1H; C(4)–H), 2.43 (dt, *J* = 11.7, 8.9 Hz, 1H; C(4)–H).

^{13}C NMR ($CDCl_3$, 101 MHz): δ (ppm) 204.2 (C=O), 173.8 (CO_2CH_3), 171.8 (CO_2CH_3), 163.0 (C(2')), 136.9 (C(4')), 130.2 (C(6')), 119.3 (C(5')), 118.7 (C(3')), 117.8 (C(1')), 52.33 (OCH_3), 52.27 (OCH_3), 41.9 (C(3)), 40.2 (C(2)), 38.0 (C(1)), 26.8 (C(4)).

IR ($CHCl_3$ film): 2953 (w), 1736 (s), 1634 (s), 1487 (w), 1437 (m), 1308 (m), 1272 (m), 1205 (s), 1160 (m), 1034 (w), 955 (w), 758 (m) cm^{-1}.

HRMS (ESI): m/z calcd for $C_{15}H_{16}O_6Na^+$ $[M + Na]^+$ 315.0839 found 315.0840.

SFC Chiralpak® IG 1500 psi, 30 °C; flow: 1.5 mL/min; from 1% to 30% MeOH in 5 min, then from 30 to 50% in 0.5 min; 98:2 e.r. (minor enantiomer t_R = 4.03 min; major enantiomer t_R = 5.35 min).

$[\alpha]^{25}_D$ = –98.9 (*c* = 1.0, $CHCl_3$).

m.p.: 83 – 85 °C.

Synthesis of 4d

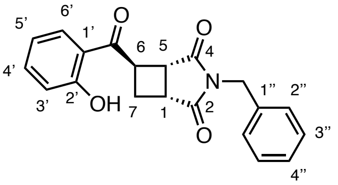

The corresponding compound was prepared following general procedure A using salicylaldehyde and cyclobutene **2d**. The mixture was stirred at 60 °C for 2 h. ^{1}H NMR analysis of the unpurified reaction mixture showed a diastereomeric ratio of >5:1. Purification by automated medium-pressure chromatography (hexane/EtOAc = 100/0 to 70/30) afforded the product **4d** as a colorless solid (106 mg, 79% yield). SFC analysis showed an enantiomeric excess of 63%.

^{1}H NMR ($CDCl_3$, 400 MHz): δ (ppm) 11.93 (s, 1H; OH), 7.66 (dd, *J* = 8.0, 1.6 Hz, 1H; C(6')–H), 7.50 (ddd, *J* = 8.6, 7.2, 1.6 Hz, 1H; C(3')–H), 7.47 – 7.41 (m, 2H; 2x C(2'')–H), 7.39 – 7.28 (m, 3H; 2x C(3'')–H, C(4'')–H), 7.02 (dd, *J* = 8.5, 1.1 Hz, 1H; C(3')–H), 6.93 (ddd, *J* = 8.2, 7.2, 1.2 Hz, 1H; C(5')–H), 4.76 (s, 2H; CH_2Ph), 4.04 (dddd, *J* = 9.6, 6.5, 4.5, 1.1 Hz, 1H; C(6)–H), 3.73 (ddt, *J* = 6.7, 4.6, 1.0 Hz, 1H; C(5)–H), 3.30 (dddd, *J* = 10.7, 6.9, 4.6, 1.0 Hz, 1H; C(1)–H), 3.05 (dddd, *J* = 13.2, 10.8, 6.5, 1.0 Hz, 1H; C(7)–H), 2.48 (dddd, *J* = 13.2, 9.6, 4.6, 0.9 Hz, 1H; C(7)–H).

^{13}C NMR ($CDCl_3$, 101 MHz): δ (ppm) 202.2 (C=O), 178.3 (CON), 177.7 (CON), 163.2 (C(2')), 137.2 (C(4')), 135.8 (C(1'')), 130.0 (C(6')), 129.0 (2x C(2''), 2x C(3'')), 128.3 (C(4'')), 119.6 (C(5')), 119.0 (C(3')), 117.1 (C(1')), 43.0 (CH_2Ph), 42.5 (C(6)), 40.8 (C(5)), 36.1 (C(1)), 25.9 (C(7)).

IR ($CHCl_3$ film): 2981 (w), 1771 (w), 1698 (s), 1637 (m), 1487 (w), 1148 (w), 1391 (m), 1342 (m), 1277 (m), 1159 (m), 756 (m), 670 (w) cm^{-1}.

HRMS (ESI): m/z calcd for $C_{20}H_{17}O_4NNa^+$ $[M + Na]^+$ 358.1050 found 358.1053.

SFC Chiralpak® IA 1500 psi, 30 °C; flow: 1.5 mL/min; from 1% to 30% MeOH in 5 min, then from 30% to 50% MeOH in 0.5 min, then hold 50% MeOH for 1.5 min; 81.5:18.5 e.r. (minor enantiomer t_R = 4.56 min; major enantiomer t_R = 6.33 min).

$[\alpha]^{25}_{D}$ = –81.2 (*c* = 1.0, $CHCl_3$).

m.p.: 144 – 154 °C.

Synthesis of 4e

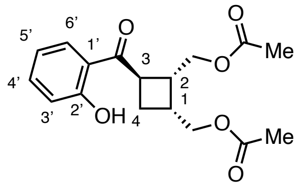

The corresponding compound was prepared following general procedure A using salicylaldehyde and cyclobutene **4e** with an increased catalyst loading of $[Rh(cod)OH]_2$ (5.0 mol%) and (*R*,*R*)-MeDuphos (12 mol%). The mixture was stirred at 60 °C for 2 h. ^{1}H NMR analysis of the unpurified reaction mixture showed a diastereomeric ratio of approximately 6:1. Purification by automated medium-pressure chromatography (hexane/EtOAc = 100/0 to 70/30) afforded the product **4e** as a colorless oil (99 mg, 78% yield). SFC analysis showed an enantiomeric excess of 95%.

^{1}H NMR ($CDCl_3$, 400 MHz): δ (ppm) 12.25 (s, 1H; OH), 7.60 (dd, *J* = 8.1, 1.7 Hz, 1H; C(6')–H), 7.46 (ddd, *J* = 8.7, 7.2, 1.7 Hz, 1H; C(4')–H), 6.99 (dd, *J* = 8.4, 1.2 Hz, 1H; C(3')–H), 6.88 (ddd, *J* = 8.2, 7.2, 1.2 Hz, 1H; C(5')–H), 4.31 – 4.20 (m, 4H; 2x CH_2O), 3.95 (dtd, *J* = 9.0, 7.8, 1.1 Hz, 1H; C(3)–H), 3.46 – 3.02 (m, 1H; C(2)–H), 2.83 – 2.73 (m, 1H; C(1)–H), 2.44 (dddd, *J* = 11.8, 8.8, 7.9, 0.7 Hz, 1H; C(4)–H), 2.17 (dddd, *J* = 11.9, 9.8, 4.8, 0.9 Hz, 1H; C(4)–H), 2.07 (s, 3H; CH_3), 2.00 (s, 3H; CH_3).

^{13}C NMR ($CDCl_3$, 101 MHz): δ (ppm) 205.5 (C=O), 171.1 (CH_3CO_2), 170.9 (CH_3CO_2), 163.0 (C(2')), 136.6 (C(4')), 129.9 (C(6')), 119.0 (C(5')), 118.8 (C(3')), 118.2 (C(1')), 64.5 (CH_2O), 63.6 (CH_2O), 42.2 (C(3)), 36.6 (C(2)), 32.2 (C(1)), 26.4 (C(4)), 21.0 (CH_3), 20.9 (CH_3).

IR ($CHCl_3$ film): 2982 (s), 2971 (s), 2889 (w), 1740 (m), 1635 (m), 1382 (m), 1239 (m), 1035 (w), 957 (w), 758 (w) cm^{-1}.

HRMS (ESI): m/z calcd for $C_{17}H_{20}O_6Na^+$ $[M + Na]^+$ 343.1152 found 343.1153.

SFC Chiralpak® IF 1500 psi, 30 °C; flow: 1.5 mL/min; from 1% to 30% MeOH in 5 min; 97.5:2.5 e.r. (minor enantiomer t_R = 2.19 min; major enantiomer t_R = 2.42 min).

$[\alpha]^{25}_D$ = –56.6 (*c* = 1.0, $CHCl_3$).

Synthesis of 4f

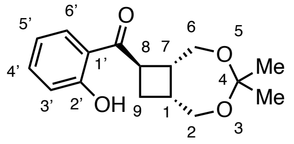

The corresponding compound was prepared following general procedure A using salicylaldehyde and cyclobutene **2f**. The mixture was stirred at 60 °C for 2 h. ^{1}H NMR analysis of the unpurified reaction mixture showed a diastereomeric ratio of >20:1. Purification by automated medium-pressure chromatography (hexane/EtOAc = 100/0 to 80/20) afforded the product **4f** as a colorless oil which solidified at –20 °C (98 mg, 89% yield). SFC analysis showed an enantiomeric excess of 97%.

^{1}H NMR ($CDCl_3$, 400 MHz): δ (ppm) 12.38 (s, 1H; OH), 7.70 (dd, *J* = 8.0, 1.7 Hz, 1H; C(6')–H), 7.46 (ddd, *J* = 8.6, 7.2, 1.6 Hz, 1H; C(4')–H), 6.98 (dd, *J* = 8.4, 1.1 Hz, 1H; C(3')–H), 6.91 (ddd, *J* = 8.2, 7.2, 1.2 Hz, 1H; C(5')–H), 4.30 – 4.22 (m, 1H; C(8)–H), 4.00 – 3.90 (m, 2H; CH_2O), 3.81 (dd, *J* = 12.5, 6.1 Hz, 1H; 1x CH_2O), 3.55 (dd, *J* = 13.5, 3.0 Hz, 1H; 1x CH_2O), 2.81 (tt, *J* = 8.6, 2.9 Hz, 1H; C(7)–H), 2.62 – 2.50 (m, 1H; C(1)–H), 2.41 (ddd, *J* = 11.2, 8.9, 8.1 Hz, 1H; C(9)–H), 1.89 (ddd, *J* = 11.3, 8.6, 2.7 Hz, 1H; C(9)–H), 1.46 (s, 3H; CH_3), 1.39 (s, 3H; CH_3).

^{13}C NMR ($CDCl_3$, 101 MHz): δ (ppm) 206.6 (C=O), 162.9 (C(2')), 136.4 (C(4')), 130.4 (C(6')), 119.1 (C(5')), 118.8 (C(3')), 118.6 (C(1')), 102.6 (C(4)), 63.4 (CH_2O), 61.8 (CH_2O), 41.4 (C(8)), 40.8 (C(7)), 34.9 (C(1)), 25.7 (CH_3), 25.2 (C(9)), 24.1 (CH_3).

IR ($CHCl_3$ film): 2982 (s), 1633 (s), 1612 (m), 1487 (m), 1447 (m), 1382 (m), 1286 (m), 1239 (m), 1214 (s), 1151 (s), 1059 (m), 850 (w), 757 (m) cm^{-1}.

HRMS (ESI): m/z calcd for $C_{16}H_{19}O_4^-$ $[M – H]^-$ 275.1289 found 275.1286.

SFC Chiralpak® IF 1500 psi, 30 °C; flow: 1.5 mL/min; from 1% to 30% MeOH in 5 min; 98.5:1.5 e.r. (minor enantiomer t_R = 3.39 min; major enantiomer t_R = 3.95 min).

$[\alpha]^{25}_D$ = –113.0 (*c* = 1.0, $CHCl_3$).

m.p.: 56 – 58 °C

Synthesis of 6a

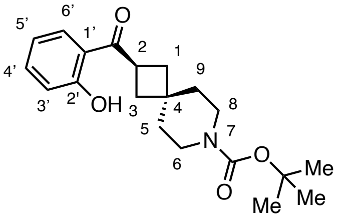

The corresponding compound was prepared following general procedure B using salicylaldehyde and cyclobutene **5**. The mixture was stirred at 60 °C for 20 h. Purification by automated medium-pressure chromatography (hexane/EtOAc = 100/0 to 70/30) afforded the product **6a** as a colorless oil (116 mg, 84% yield).

^{1}H NMR ($CDCl_3$, 400 MHz): δ (ppm) 12.32 (s, 1H; OH), 7.57 (dd, *J* = 8.0, 1.7 Hz, 1H; C(6')–H), 7.45 (ddd, *J* = 8.6, 7.2, 1.7 Hz, 1H; C(4')–H), 6.99 (dd, *J* = 8.4, 1.1 Hz, 1H; C(3')–H), 6.87 (ddd, *J* = 8.3, 7.2, 1.2 Hz, 1H; C(5')–H), 3.97 (tt, *J* = 9.4, 8.1 Hz, 1H; 1x C(2)–H), 3.45 – 3.37 (m, 2H; 1x C(6)–H_2, 1x C(8)–H_2), 3.32 – 3.13 (m, 2H; 1x C(6)–H_2, 1x C(8)–H_2), 2.29 – 2.20 (m, 2H; 1x C(1)–H_2, 1x C(3)–H_2), 2.20 – 2.12 (m, 2H; 1x C(1)–H_2, 1x C(3)–H_2), 1.72 – 1.66 (m, 2H; 1x C(5)–H_2, 1x C(9)–H_2), 1.53 – 1.48 (m, 2H; 1x C(5)–H_2, 1x C(9)–H_2), 1.45 (s, 9H; $C(CH_3)_3$).

^{13}C NMR ($CDCl_3$, 101 MHz): δ (ppm) 207.3 (C=O), 162.8 (C(2')), 155.0 (NCO_2), 136.3 (C(4')), 129.9 (C(6')), 118.9 (C(5')), 118.7 (C(3')), 118.1 (C(1')), 79.5 (*C*(CH_3)$_3$), 40.6 (rot. br.; C(2), C(8)), 38.2 (C(5)/C(9)), 36.9 (C(5)/C(9)), 35.8 (C(2)), 34.7 (C(1), C(3)), 34.4 (C(4)), 28.6 (C(*C*H_3)$_3$).

IR ($CHCl_3$ film): 3008 (w), 2974 (w), 2927 (w), 2848 (w), 1682 (m), 1635 (m), 1485 (w), 1391 (m), 1365 (m), 1273 (m), 1241 (s), 1206 (m), 1174 (m), 1147 (s), 968 (m), 892 (w), 750 (s), 649 (w) cm^{-1}.

HRMS (ESI): m/z calcd for $C_{20}H_{27}O_4NNa^+$ $[M + Na]^+$ 368.1832 found 368.1833.

Synthesis of 6b

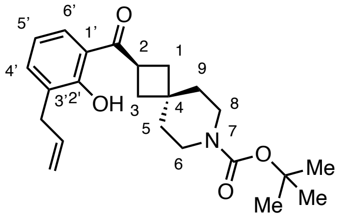

The corresponding compound was prepared following general procedure B using 3-allylsalicylaldehyde and cyclobutene **5**. The mixture was stirred at 60 °C for 20 h. Purification by automated medium-pressure chromatography (hexane/EtOAc = 100/0 to 70/30) afforded the product **6a** as a pale-yellow oil (124 mg, 81% yield).

^{1}H NMR ($CDCl_3$, 400 MHz): δ (ppm) 12.67 (s, 1H; OH), 7.46 (dd, *J* = 8.1, 1.6 Hz, 1H; C(6')–H), 7.33 (dd, *J* = 7.4, 1.6 Hz, 1H; C(4')–H), 6.81 (t, *J* = 7.7 Hz, 1H; C(5')–H), 6.07 – 5.94 (m, 1H; C*H*=CH_2), 5.12 – 5.08 (m, 1H; 1x CH=CH_2), 5.07 (q, *J* = 1.2 Hz, 1H; 1x CH=CH_2), 3.97 (app. p, *J* = 8.8 Hz, 1H; C(2)–H), 3.43 (d, *J* = 6.7 Hz, 2H; 1x C(6)–H_2, 1x C(8)–H_2), 3.45 – 3.36 (m, 2H; 1x C(6)–H_2, 1x C(8)–H_2), 3.33 – 3.24 (m, 2H; 1x C(1)–H_2, 1x C(3)–H_2), 2.30 – 2.20 (m, 2H; 1x C(1)–H_2, 1x C(3)–H_2), 2.20 – 2.11 (m, 2H), 1.68 (t, *J* = 5.7 Hz, 2H; 1x C(5)–H_2, 1x C(9)–H_2), 1.49 (t, *J* = 5.7 Hz, 2H; 1x C(5)–H_2, 1x C(9)–H_2), 1.45 (s, 9H; $C(CH_3)_3$).

^{13}C NMR ($CDCl_3$, 101 MHz): δ (ppm) 207.5 (C=O), 160.8 (C(2')), 155.0 (NCO_2), 136.3, 136.2 (C(4'), CH_2*C*H=CH_2), 129.7 (C(3')), 128.0 (C(6')), 118.5 (C(5')), 117.7 (C(1')), 116.1 ($CH_2CH=$*C*H_2), 79.5 (*C*$(CH_3)_3$), 40.6 (rot. br.; C(2), C(8)), 38.2 (C(5)/C(9)), 36.9 (C(5)/C(9)), 35.9 (C(2)), 34.8 (C(1), C(3)), 34.4 (C(4)), 33.6 (*C*$H_2CH=CH_2$), 28.6 (C(*C*H_3)$_3$).

IR ($CHCl_3$ film): 3007 (w), 2975 (w), 2926 (w), 2847 (w), 1685 (m), 1629 (m), 1426 (m), 1392 (m), 1271 (m), 1242 (m), 1174 (m), 1147 (m), 983 (m), 916 (w), 750 (s), 665 (w) cm^{-1}.

HRMS (ESI): m/z calcd for $C_{23}H_{31}O_4NNa^+$ $[M + Na]^+$ 408.2145 found 408.2143.

Synthesis of 6c

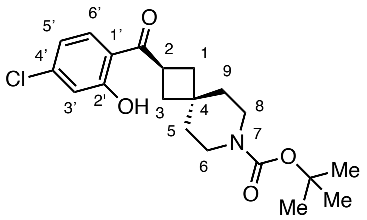

The corresponding compound was prepared following general procedure B using 4-chlorosalicylaldehyde and cyclobutene **5**. The mixture was stirred at 60 °C for 20 h. Purification by automated medium-pressure chromatography (hexane/EtOAc = 100/0 to 70/30) afforded the product **6a** as a colorless solid (118 mg, 78% yield).

^{1}H NMR ($CDCl_3$, 400 MHz): δ (ppm) 12.45 (s, 1H; OH), 7.49 (d, *J* = 8.6 Hz, 1H; C(6')–H), 7.00 (d, *J* = 2.0 Hz, 1H; C(3')–H), 6.84 (dd, *J* = 8.6, 2.1 Hz, 1H; C(5')–H), 3.91 (tt, *J* = 9.4, 8.1 Hz, 1H; C(2)–H), 3.43 – 3.36 (m, 2H; 1x C(6)–H_2, 1x C(8)–H_2), 3.33 – 3.25 (m, 2H; 1x C(6)–H_2, 1x C(8)–H_2), 2.27 – 2.20 (m, 2H; 1x C(1)–H_2, 1x C(3)–H_2), 2.19 – 2.10 (m, 2H; 1x C(1)–H_2, 1x C(3)–H_2), 1.70 – 1.65 (m, 2H; 1x C(5)–H_2, 1x C(9)–H_2), 1.49 (dd, *J* = 6.8, 4.6 Hz, 2H; 1x C(5)–H_2, 1x C(9)–H_2), 1.45 (s, 9H; $C(CH_3)_3$).

^{13}C NMR ($CDCl_3$, 101 MHz): δ (ppm) 206.5 (C=O), 163.5 (C(2')), 155.0 (NCO_2), 142.0 (C(1')), 130.9 (C(6')), 119.6 (C(5')), 118.8 (C(3')), 116.7 (C(1')), 79.6 ($\mathit{C}(CH_3)_3$), 40.7 (rot. br.; C(6), C(8)), 38.2 (C(5)/C(9)), 36.9 (C(5)/C(9)), 35.8 (C(2)), 34.7 (C(1), C(3)), 34.4 (C(4)), 28.6 ($C(\mathit{C}H_3)_3$).

IR ($CHCl_3$ film): 2975 (w), 2973 (w), 1684 (m), 1634 (m), 1422 (m), 1364 (m), 1270(m), 1241 (s), 1175 (s), 1145 (s), 1080 (m), 970 (m), 937 (m), 861 (m), 796 (m), 754 (s) cm^{-1}.

HRMS (ESI): m/z calcd for $C_{20}H_{26}O_4N^{35}ClNa^+$ $[M + Na]^+$ 402.1443 found 402.1444.

m.p.: 103 – 104 °C.

Synthesis of 6d

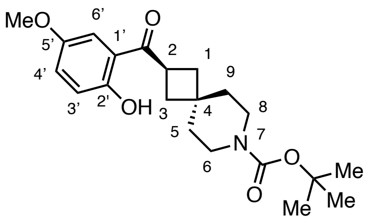

The corresponding compound was prepared following general procedure B using 5-methoxysalicylaldehyde and cyclobutene **5**. The mixture was stirred at 60 °C for 20 h. Purification by automated medium-pressure chromatography (hexane/EtOAc = 100/0 to 70/30) afforded the product **6d** as a yellow oil (122 mg, 80% yield).

^{1}H NMR ($CDCl_3$, 400 MHz): δ (ppm) 11.89 (s, 1H; OH), 7.09 (dd, *J* = 9.0, 3.1 Hz, 1H; C(4')–H), 7.02 (d, *J* = 3.1 Hz, 1H; C(6')–H), 6.93 (d, *J* = 9.0 Hz, 1H; C(3')–H), 3.92 (tt, *J* = 9.4, 8.0 Hz, 1H; C(2)–H), 3.78 (s, 3H; OCH_3), 3.42 – 3.37 (m, 2H; 1x C(6)–H_2, 1x C(8)–H_2), 3.33 – 3.26 (m, 2H; 1x C(6)–H_2, 1x C(8)–H_2), 2.30 – 2.21 (m, 2H; 1x C(1)–H_2, 1x C(3)–H_2), 2.20 – 2.12 (m, 2H; 1x C(1)–H_2, 1x C(3)–H_2), 1.70 – 1.65 (m, 2H; 1x C(5)–H_2, 1x C(9)–H_2), 1.49 (dd, *J* = 6.7, 4.7 Hz, 2H; 1x C(5)–H_2, 1x C(9)–H_2), 1.45 (s, 9H; $C(CH_3)_3$).

^{13}C NMR ($CDCl_3$, 101 MHz): δ (ppm) 206.8 (C=O), 157.2 (C(2')), 155.0 (NCO_2), 151.8 (C(5')), 123.6 (C(4')), 119.5 (C(3')), 117.7 (C(1')), 113.3 (C(6')), 79.5 ($\mathit{C}(CH_3)_3$), 56.2 (OCH_3), 40.5 (rot. br.; C(2), C(8)), 38.2 (C(5)/C(9)), 36.9 (C(5)/C(9)), 35.9 (C(2)), 34.7 (C(1), C(3)), 34.4 (C(4)), 28.6 ($C(\mathit{C}H_3)_3$).

IR ($CHCl_3$ film): 2926 (w), 2846 (w), 1687 (m), 1641 (w), 1614 (w), 1485 (m), 1392 (m), 1364 (m), 1243 (s), 1174 (s), 1146 (s), 1119 (s), 970 (m), 772 (m) cm^{-1}.

HRMS (ESI): m/z calcd for $C_{21}H_{29}O_5NNa^+$ $[M + Na]^+$ 398.1938 found 398.1938.

Synthesis of 6e

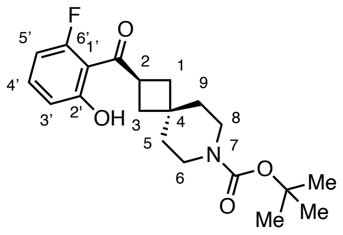

The corresponding compound was prepared following general procedure B using 6-fluorosalicylaldehyde and cyclobutene **5** with an increased catalyst loading of $[Rh(cod)OH]_2$ (5.0 mol%) and dppf (12 mol%). The mixture was stirred at 60 °C for 20 h. Purification by automated medium-pressure chromatography (hexane/EtOAc = 100/0 to 70/30) afforded the product **6e** as a colorless solid (84 mg, 58% yield).

^{1}H NMR ($CDCl_3$, 400 MHz): δ (ppm) 12.81 (s, 1H; OH), 7.37 (td, *J* = 8.3, 6.4 Hz, 1H; C(4')–H), 6.78 (dt, *J* = 8.4, 1.0 Hz, 1H; C(3')–H), 6.57 (ddd, *J* = 11.7, 8.2, 1.1 Hz, 1H; C(5')–H), 3.90 (ttd, *J* = 9.3, 7.9, 3.4 Hz, 1H; C(2)–H), 3.40 – 3.35 (m, 2H; 1x C(6)–H_2, 1x C(8)–H_2), 3.32 – 3.25 (m, 2H; 1x C(6)–H_2, 1x C(8)–H_2), 2.26 – 2.10 (m, 4H; C(1)–H_2, C(3)–H_2), 1.68 – 1.62 (m, 2H; 1x C(5)–H_2, 1x C(9)–H_2), 1.51 – 1.46 (m, 2H; 1x C(5)–H_2, 1x C(9)–H_2), 1.45 (s, 9H; $C(CH_3)_3$).

^{13}C NMR ($CDCl_3$, 101 MHz): δ (ppm) 206.11 (d, *J* = 4.3 Hz; C=O), 164.4 (d, *J* = 5.2 Hz; C(2'))), 163.01 (d, *J* = 256.0 Hz; C(6')), 155.1 (NCO_2), 136.1 (d, *J* = 13.1 Hz; C(4')), 114.5 (d, *J* = 3.2 Hz; C(3')), 108.8 (d, *J* = 14.7 Hz; C(1')), 106.2 (d, *J* = 24.6 Hz; C(5')), 79.5 ($\mathit{C}(CH_3)_3$), 40.4 (d, *J* = 11.1 Hz; C(2)), 38.2 (C(5)/C(9)), 37.0 (C(5)/C(9)), 34.0 (d, *J* = 4.0 Hz; C(1), C(3)), 33.8 (C(4)), 28.6 ($C(\mathit{C}H_3)_3$)

^{19}F NMR ($CDCl_3$, 376 MHz): δ (ppm) -107.13 (br. s).

IR ($CHCl_3$ film): 2978 (w), 2927 (w), 2848 (w), 1689 (s), 1638 (s), 1615 (m), 1451 (s), 1421 (s), 1364 (s), 1242 (s), 1222 (s), 1200 (s), 1174 (s), 1146 (s), 1121 (m), 1027 (s), 965 (m), 796 (s), 744 (m) cm^{-1}.

HRMS (ESI): m/z calcd for $C_{20}H_{26}O_4FNNa^+$ $[M + Na]^+$ 386.1738 found 386.1737.

m.p.: 65 – 67 °C.

1.4. Upscale and derivatization of products

Gram-scale synthesis of 3a

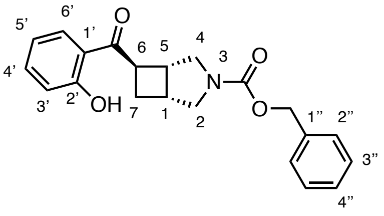

The corresponding compound was prepared in direct analogy to general procedure A using salicylaldehyde (4.0 mmol) and cyclobutene **2** (4.8 mmol). The mixture was stirred at 60 °C for 1 h. Purification by automated medium-pressure chromatography (hexane/EtOAc = 100/0 to 70/30) afforded the product **3a** as a colorless solid (1.18 g, 84% yield). SFC analysis showed an enantiomeric excess of 98%.

Along with **3a**, unreacted cyclobutene **2** (156 mg, 0.68 mmol) was recovered and the minor diastereoisomer **3a'** (158 mg, 11%) was isolated as a colorless oil. SFC analysis of **3a'** showed an enantiomeric excess of 98%. The absolute stereochemistry of **3a'** was not assigned.

*For analytical data of **3a'**:*

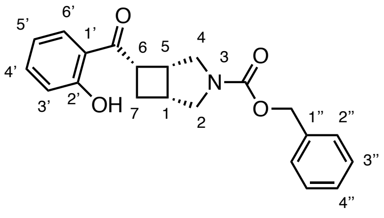

¹H NMR ($CDCl_3$, 400 MHz): δ (ppm) 12.45 – 12.07 (m, 1H; OH), 7.56 (dd, *J* = 8.1, 1.7 Hz, 1H; C(6')–H), 7.45 (ddd, *J* = 8.7, 7.2, 1.7 Hz, 1H; C(4)–H), 7.37 – 7.28 (m, 5H; 2x C(2'')–H, 2x C(3'')–H, C(4'')–H), 7.00 (dd, *J* = 8.4, 1.2 Hz, 1H; C(3')–H), 6.87 (ddd, *J* = 8.2, 7.1, 1.2 Hz, 1H; C(5')–H), 5.12 (rot. s, 2H; CH_2Ph), 4.07 (q, *J* = 8.7 Hz, 1H; C(6)–H), 3.73 – 3.55 (m, 1H; 1x CH_2N), 3.53 – 3.41 (m, 2H; 1x CH_2N, C(5)–H), 3.35 – 3.16 (m, 3H; 2x CH_2N), 3.04 (p, *J* = 7.4 Hz, 1H; C(1)–H), 2.55 (dt, *J* = 12.7, 8.1 Hz, 1H; C(7)–H), 2.41 – 2.19 (m, 1H; C(7)–H).

¹³C NMR ($CDCl_3$, 101 MHz): δ (ppm) 204.7 (C=O), 162.8 (C(2')), 155.0 (CO_2N), 137.0 (C(1'')), 136.4 (C(4')), 129.6 (C(6')), 128.5 (2x C(4'')), 128.0 (C(4'')), 127.9 (2x C(2'')), 119.1 (C(5')), 119.0 (C(3')), 118.6

(C(1’)), 66.9 (CH_2Ph), 51.9, 51.7 (rot.; 2x CH_2N), 47.8, 47.1 (rot.; 2x CH_2N), 44.0, 43.1 (rot.; 2x C(5)), 41.1, 40.8 (rot.; 2x C(6)), 35.4, 34.5 (rot.; 2x C(1)), 24.9, 24.6 (rot.; 2x C(7)).

IR ($CHCl_3$ film): 2972 (w), 2874 (w), 1699 (s), 1633 (m), 1486 (w), 1446 (m), 1418 (m), 1361 (m), 1277 (m), 1240 (m), 1212 (m), 758 (m) cm^{-1}.

HRMS (ESI): m/z calcd for $C_{21}H_{22}O_4N^+$ $[M + H]^+$ 352.1543 found 352.1540.

SFC Chiralpak® IF 1500 psi, 30 °C; flow: 1.5 mL/min; from 1% to 30% MeOH in 5 min, then from 30% to 50% MeOH in 0.5 min, then hold 50% MeOH for 1.5 min; 99:1 e.r. (minor enantiomer t_R = 5.97 min; major enantiomer t_R = 6.19 min).

$[\alpha]^{25}_D$ = –140.2 (*c* = 1.0, $CHCl_3$).

m.p.: 89 – 91 °C.

Synthesis of 7a

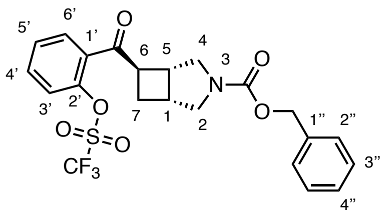

Triethylamine (280 µL, 2.0 mmol), *N*-(2-pyridyl)bis(trifluoromethanesulfonimide) (537 mg, 1.50 mmol) followed by 4-dimethylaminopyridine (12.2 mg, 100 µmol) were added to a solution of **3a** (351 mg, 1.00 mmol) in CH_2Cl_2 (5.0 mL) under an argon atmosphere at room temperature (23 °C). After stirring the mixture for 22 h, additional triethylamine (70 µL, 0.50 mmol), *N*-(2-pyridyl)bis(trifluoromethanesulfonimide) (178 mg, 0.50 mmol) were added, and stirring was continued for 1 h. The solvents were removed under reduced pressure. Purification by automated medium-pressure chromatography (hexane/EtOAc = 100/0 to 60/40) afforded the product **7a** as a pale-yellow oil (445 mg, 92% yield).

^{1}H NMR ($CDCl_3$, 400 MHz): δ (ppm) 7.65 – 7.56 (m, 2H; C(4')–H, C(6')–H), 7.46 (app. td, *J* = 7.6, 1.2 Hz, 1H; C(5')–H), 7.42 – 7.29 (m, 6H; 2x C(2'')–H, 2x C(3'')–H, C(4'')–H, C(3')–H), 5.19 (s, 2H; CH_2Ph), 3.75 (rot. s, 2H; 2x CH_2N), 3.62 (s, 1H; C(6)–H), 3.47 – 3.25 (m, 3H; 2x CH_2N, C(5)–H), 2.91 (s, 1H; C(1)–H), 2.56 (br. s, 1H; C(7)–H), 2.03 (br. s, 1H; C(7)–H).

^{13}C NMR ($CDCl_3$, 101 MHz): δ (ppm) 199.2 (C=O), 155.7 (CO_2N), 147.3 (C(2')), 136.9 (C(1'')), 133.9 (C(4')), 130.6 (C(2')), 130.3 (C(6')), 128.7 (C(5')), 128.6 (2x C(3'')), 128.14 (C(4'')), 128.05 (2x C(2'')), 123.2 (C(3')) 118.8 (q, *J* = 320.3 Hz; CF_3), 67.1 (CH_2Ph), 53.0 , 52.4 (rot.; 2x CH_2N), 46.5 (C(6)), 40.2 (rot.; C(5)), 34.6, 33.6 (rot.; 2x C(1)), 28.5, 28.0 (rot.; 2x C(7)).

^{19}F NMR ($CDCl_3$, 376 MHz): δ (ppm) -73.34 (s).

IR ($CHCl_3$ film): 2944 (w), 1694 (s). 1605 (w), 1423 (s), 1359 (m), 1211 (s), 1139 (m), 1113 (m) 903 (m), 769 (w), 698 (w) cm^{-1}.

HRMS (ESI): m/z calcd for $C_{22}H_{21}O_6NF_3S^+$ $[M + H]^+$ 484.1036 found 484.1036.

[α]$^{25}_D$ = –58.2 (*c* = 1.0, $CHCl_3$).

Synthesis of 7b

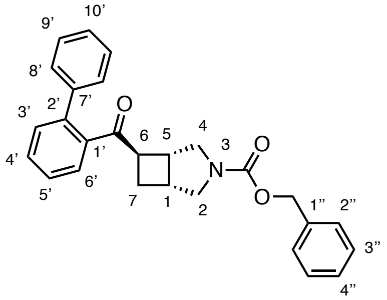

K_2CO_3 (34.6 mg, 250 µmol) and [Pd(PPh_3)$_4$] (5.8 mg, 5.0 µmol) were added to a solution of **7a** (48.4 mg, 100 µmol) and phenylboronic acid (24.4 mg, 200 µmol) in toluene (1.0 mL) under an argon atmosphere. The mixture was stirred for 16 h at 110 °C. The mixture was then filtered over a short plug of silica, rinsed with EtOAc, and the solvents were removed under reduced pressure. Purification by automated medium-pressure chromatography (hexane/EtOAc = 100/0 to 70/30) afforded the product **7b** as a colorless oil (28 mg, 67% yield).

^{1}H NMR ($CDCl_3$, 400 MHz): δ (ppm) 7.51 (app. td, *J* = 7.3, 1.7 Hz, 1H; 1x C(Ar)–H), 7.47 – 7.19 (m, 13H; 13x C(Ar)–H), 5.10 (s, 2H; CH_2Ph), 3.46 (rot. s, 1H; 1x CH_2N), 3.22 (dd, *J* = 11.7, 7.5 Hz, 1H; 1x CH_2N), 3.08 – 2.63 (br. rot. m, 5H; 2x CH_2N, C(1)–H , C(5)–H, C(6)–H), 2.25 (dt, *J* = 12.3, 8.4 Hz, 1H; C(7)–H), 1.54 (br. s, 1H; C(7)–H).

^{13}C NMR ($CDCl_3$, 101 MHz; C=O not detected): δ (ppm) 155.3 (CO_2N), 140.5 (C(Ar)$_{quart}$), 140.1 (C(Ar)$_{quart}$), 137.0 (C(1'')), 130.8, 130.3, 128.9, 128.9, 128.6, 128.10, 128.08, 128.0, 127.9, 127.6 (14x C(Ar)–H, C(Ar)$_{quart}$), 66.8 (CH_2Ph), 52.7, 51.7 (rot.; 2x CH_2N), 48.0 (C(6)), 41.3, 40.8 (rot.; 2x C(5)), 33.3 (rot.; C(1)), 28.9, 28.7 (rot.; 2x C(7)).

IR ($CHCl_3$ film): 2971 (w), 2869 (w), 1702 (w), 1416 (m), 1358 (m), 1229 (m), 1211 (m) 1158 (w), 1098 (m), 954 (w), 746 (m), 699 (m) cm^{-1}.

HRMS (ESI): m/z calcd for $C_{27}H_{26}O_3N^+$ [M + H]$^+$ 412.1907 found 412.1913.

[α]^{25}$_D$ = –40.6 (*c* = 1.0, $CHCl_3$).

Synthesis of 7c

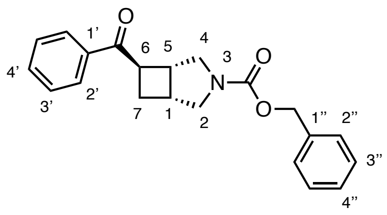

Acetonitrile (1.0 mL) was added to mixture of **7a** (48.4 mg, 100 µmol), K_2CO_3 (13.8 mg, 100 µmol), $(CH_3)_2NH·BH_3$ (6.5 mg, 110 µmol) and $[Pd(PPh_3)_4]$ (5.8 mg, 5.0 µmol) under an argon atmosphere. The mixture was stirred at 40 °C for 6 h, then filtered over a short plug of silica, rinsed with EtOAc, and the solvents were removed under reduced pressure. Purification by automated medium-pressure chromatography (hexane/EtOAc = 100/0 to 70/30) afforded the product **7c** as a pale-yellow oil (22 mg, 67% yield).

^{1}H NMR ($CDCl_3$, 400 MHz): δ (ppm) 7.90 – 7.78 (m, 2H; 2x C(2')–H), 7.58 – 7.51 (m, 1H; C(4')–H), 7.48 – 7.28 (m, 7H; 2x C(2'')–H, 2x C(3'')–H, C(4'')–H, 2x C(3')–H), 5.21 (s, 2H; CH_2Ph), 4.04 – 3.61 (m, 3H; 2x CH_2N, C(6)–H), 3.45 – 3.22 (m, 3H; 2x CH_2N, C(5)–H), 2.96 – 2.83 (m, 1H; C(1)–H), 2.54 (br. s, 1H; C(7)–H), 2.07 (br. s, 1H; C(7)–H).

^{13}C NMR ($CDCl_3$, 101 MHz): δ (ppm) 199.9 (C=O), 155.7 (CO_2N), 137.0 (C(1'')), 135.3 (C(1')), 133.3 (C(4')), 128.8 (2x C(3')), 128.6 (2x C(3'')), 128.4 (2x C(2')), 128.1 (C(4'')), 128.0 (2x C(2'')), 67.1 (CH_2Ph), 52.9, 52.3 (rot.; 2x CH_2N), 44.5 (C(6)), 39.8 (rot.; C(5)), 34.7, 34.0 (rot.; 2x C(1)), 28.9, 28.4 (rot.; 2x C(7)).

IR ($CHCl_3$ film): 2970 (w), 2870 (w), 1701 (s), 1678 (s), 1148 (w), 1416 (m), 1359 (m), 1217 (m), 1098 (m), 769 (w), 696 (m) cm^{-1}.

HRMS (ESI): m/z calcd for $C_{21}H_{24}O_3N^+$ $[M + H]^+$ 338.1751 found 338.1746.

$[\alpha]^{25}_D$ = –91.4 (*c* = 1.0, $CHCl_3$).

Synthesis of 7d

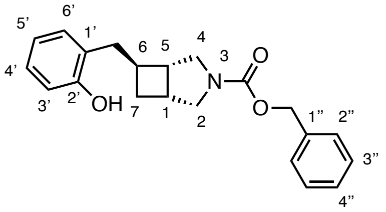

Trifluoroacetic acid (310 µL, 4.0 mmol) was added to **3a** (70 mg, 0.20 mmol) at 0 °C under an argon atmosphere. Et_3SiH (130 µL, 0.80 mmol) was then added dropwise. The mixture was then allowed to reach room temperature (23 °C) and stirring was maintained for 5 h. The solvent was removed under reduced pressure, and purification by automated medium-pressure chromatography (hexane/EtOAc = 100/0 to 70/30) afforded the product **7e** as a colorless oil (44 mg, 65% yield).

^{1}H NMR ($CDCl_3$, 400 MHz): δ (ppm) 7.43 – 7.28 (m, 5H; 2x C(2'')–H, 2x C(3'')–H, C(4'')–H), 7.10 – 6.98 (m, 2H; C(4')–H, C(6')–H), 6.82 (td, *J* = 7.4, 1.2 Hz, 1H; C(5')–H), 6.77 (dd, *J* = 7.8, 1.2 Hz, 1H; C(3')–H), 6.12 (br. s, 1H; OH), 5.18 (s, 2H; CH_2Ph), 3.68 – 3.51 (m, 2H; 2x CH_2N), 3.42 (dd, *J* = 11.6, 7.7 Hz, 1H; 1x CH_2N), 3.25 (dd, *J* = 11.5, 6.8 Hz, 1H; 1x CH_2N), 2.87 (qt, *J* = 8.8, 3.5 Hz, 1H, C(5)–H), 2.81 – 2.71 (m, 2H; C(6)HC*H_2*Ar), 2.66 (q, *J* = 6.6 Hz, 1H, C(5)–H), 2.42 (q, *J* = 7.2 Hz, 1H; C(1)–H), 2.00 – 1.90 (m, 1H; C(7)–H), 1.84 (ddd, *J* = 12.3, 8.7, 4.0 Hz, 1H; C(7)–H).

^{13}C NMR ($CDCl_3$, 101 MHz): δ (ppm) 155.8 (CO_2N), 154.2 (C(2')), 137.0 (C(1'')), 130.4 (C(6')), 128.6 (2x C(3'')), 128.0 (C(4'')), 127.9 (2x C(2'')), 127.3 (C(4')), 126.6 (C(1'')), 120.3 (C(5')), 115.3 (C(3')), 67.0 (CH_2Ph), 53.1, 52.7 (rot.; 2x CH_2N), 43.9, 43.1 (rot.:; 2x C(5)), 38.0 (C(1)), 36.2 (C(6)H*C*H_2Ar), 34.5, 33.8 (rot.: 2x C(6)), 30.8 (C(7)).

IR ($CHCl_3$ film): 3306 (br. w), 2959 (w), 1673 (s), 1594 (w), 1455 (s), 1428 m), 1360 (m), 1240 (m), 1154 (w), 1107 (m), 753 (m), 697 (w) cm^{-1}.

HRMS (ESI): m/z calcd for $C_{21}H_{22}O_3N^+$ $[M + H]^+$ 336.1594 found 336.1594.

$[\alpha]^{25}_D$ = –23.9 (*c* = 1.0, $CHCl_3$).

2. References

(1) Goetzke, F. W.; Hell, A. M. L.; van Dijk, L.; Fletcher, S. P. *Nat. Chem.* **2021***, 13*, 880.

(2) Gauvry, N.; Comoy, C.; Lescop, C.; Huet, F. *Synthesis* **1999**, 574.

3. NMR spectra

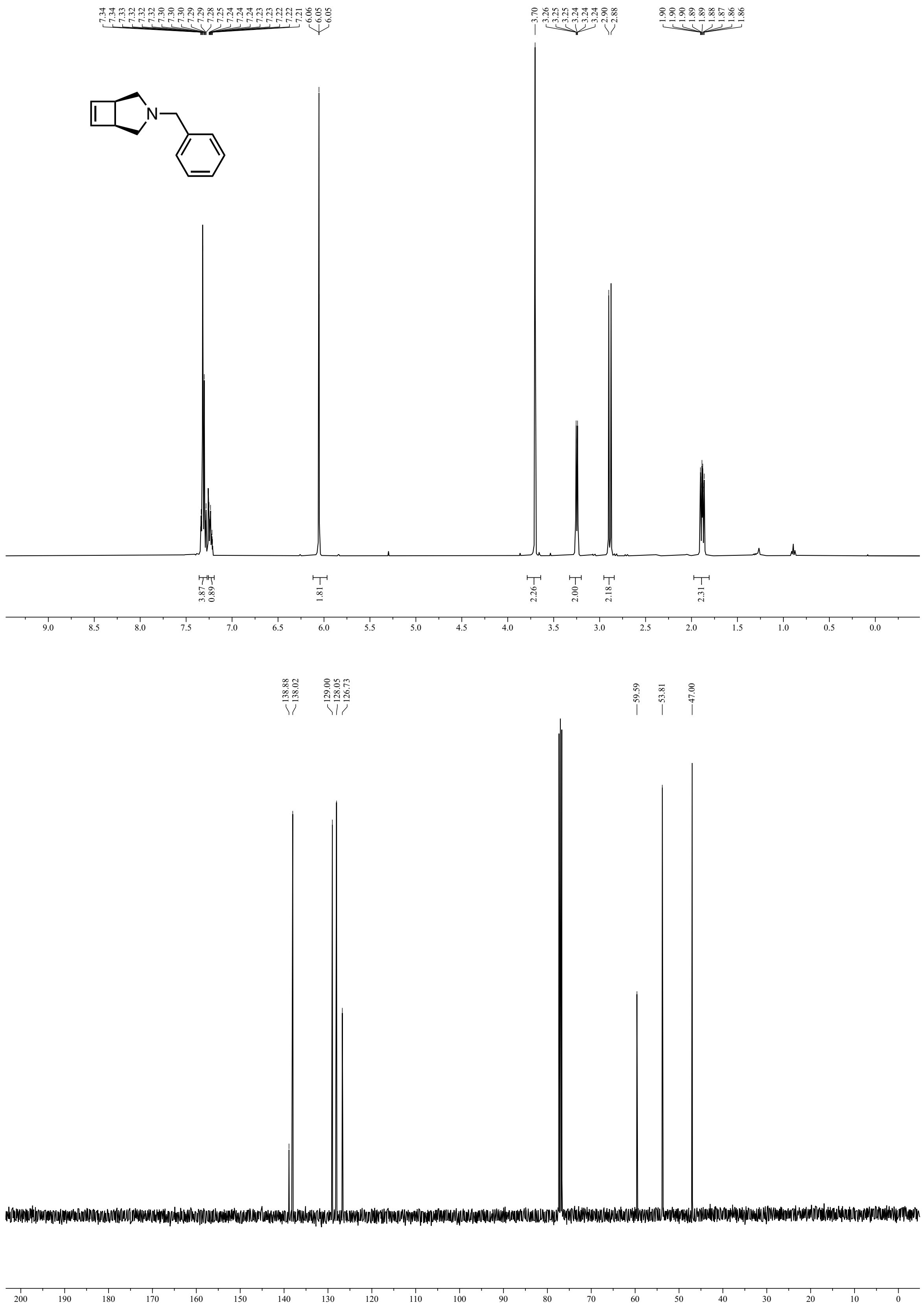

Figure S2: ^{1}H NMR (400 MHz, $CDCl_3$, top) and ^{13}C NMR (101 MHz, $CDCl_3$, bottom) spectra of **S1** recorded at 298 K.

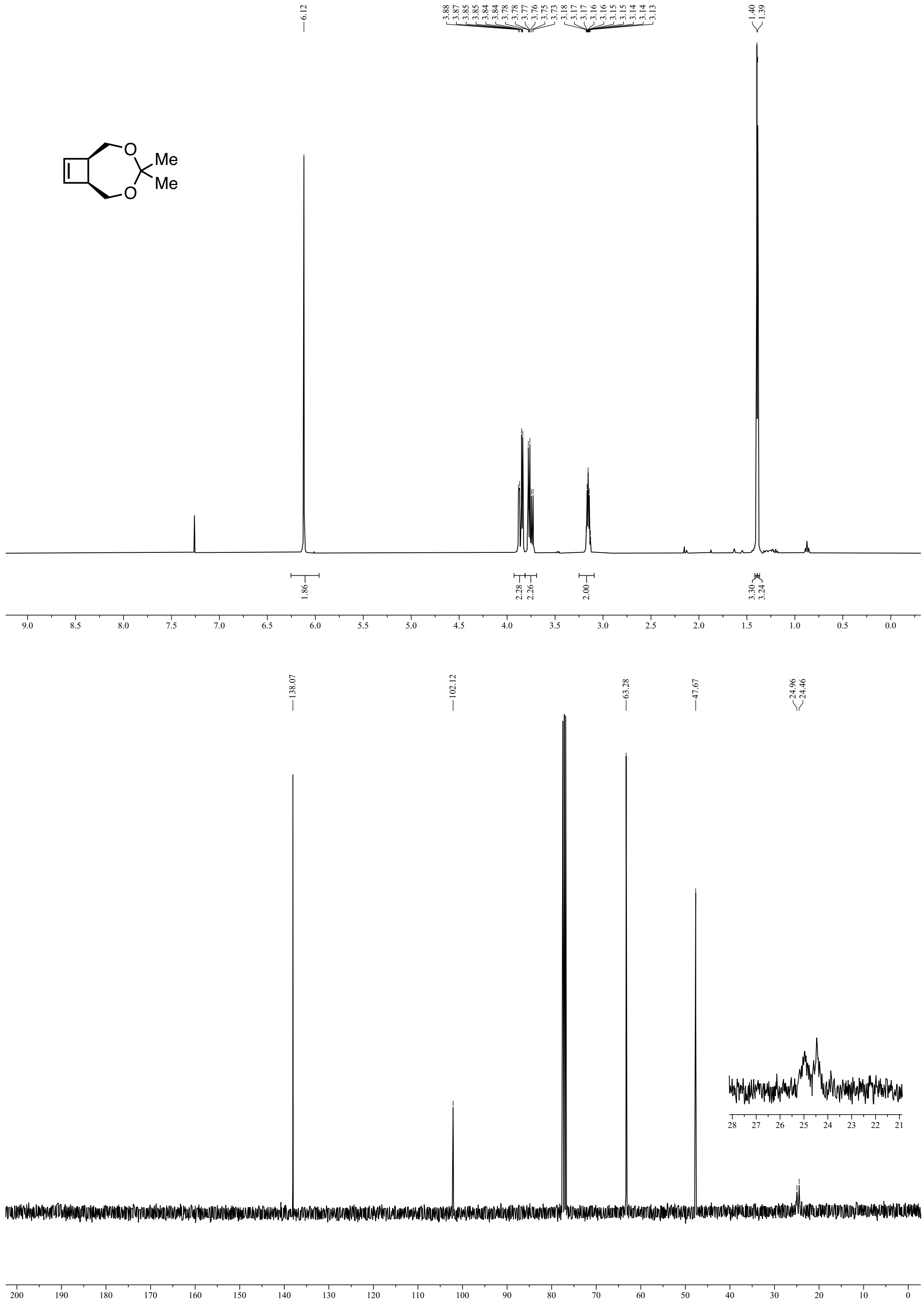

Figure S3: ^{1}H NMR (400 MHz, $CDCl_3$, top) and ^{13}C NMR (101 MHz, $CDCl_3$, bottom) spectra of **2f** recorded at 298 K.

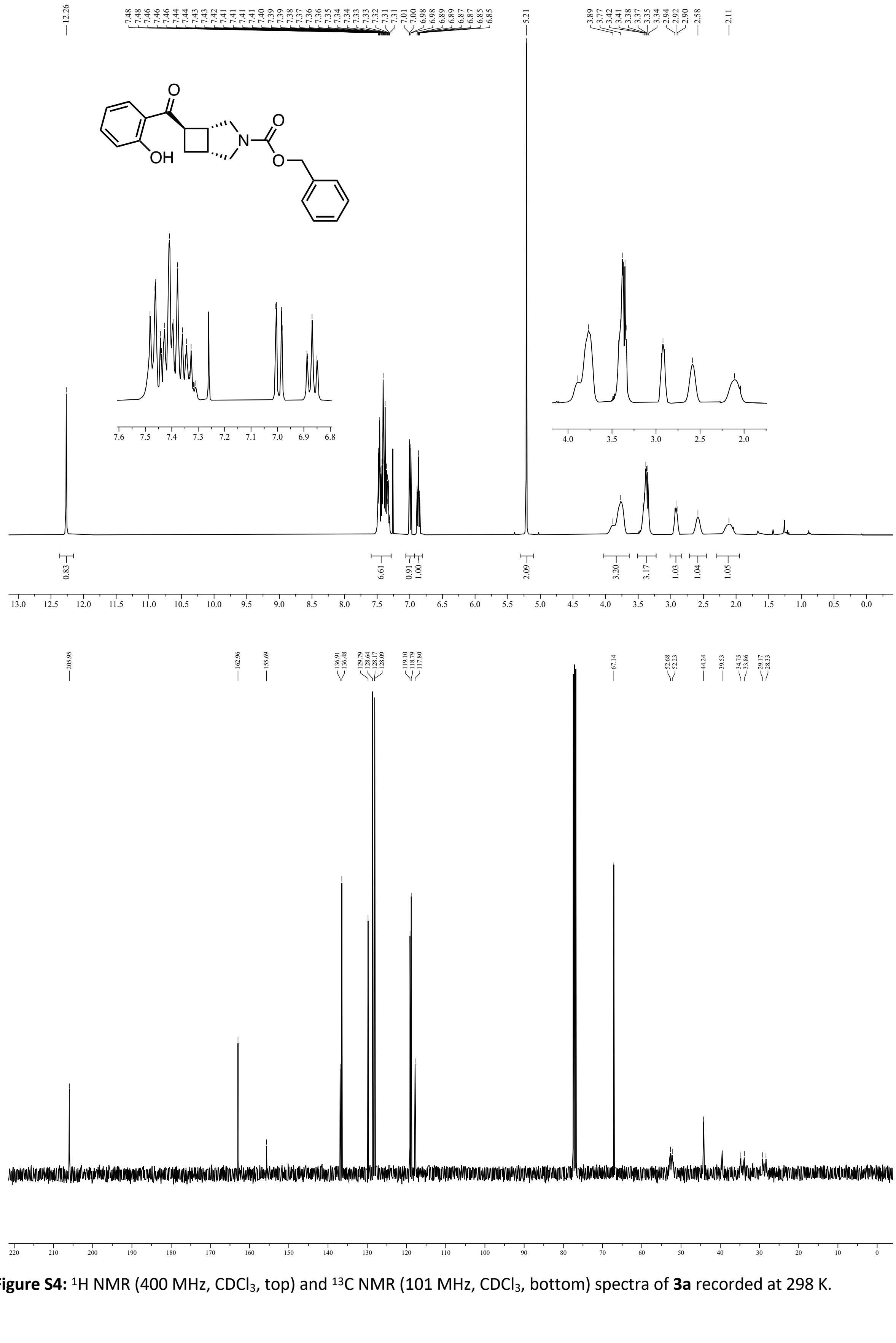

Figure S4: ^{1}H NMR (400 MHz, $CDCl_3$, top) and ^{13}C NMR (101 MHz, $CDCl_3$, bottom) spectra of **3a** recorded at 298 K.

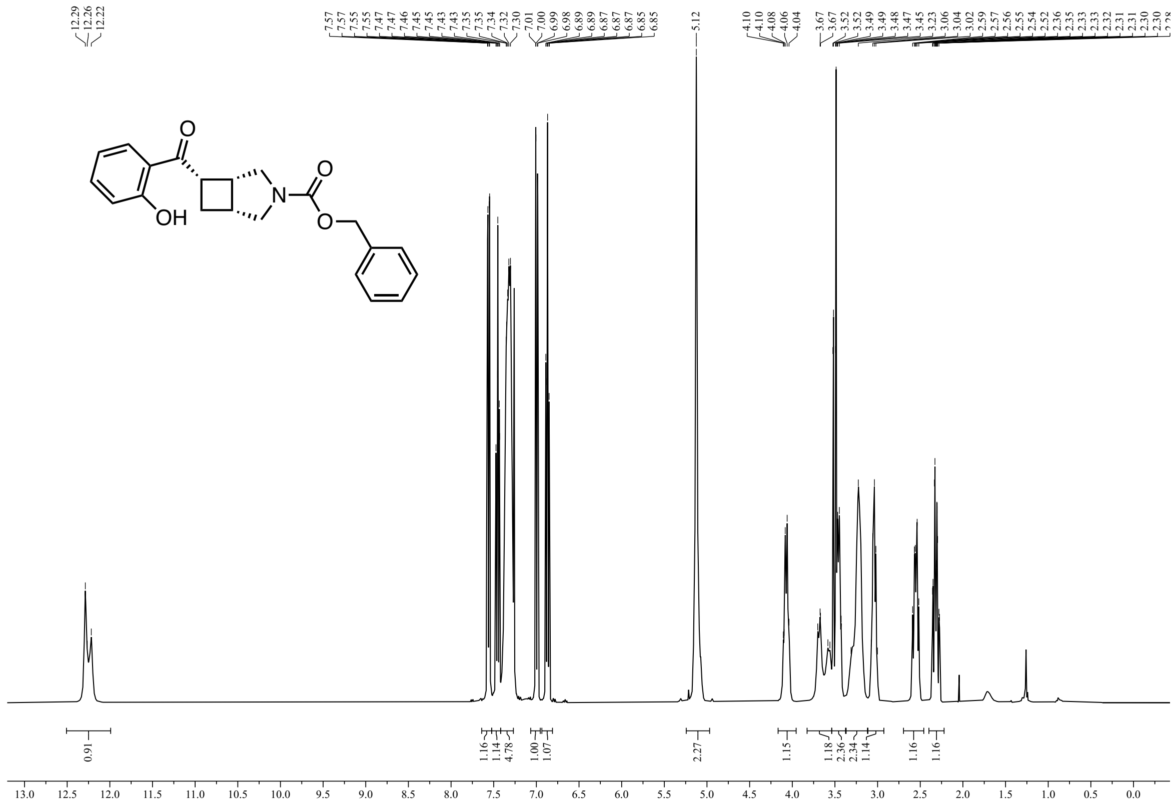

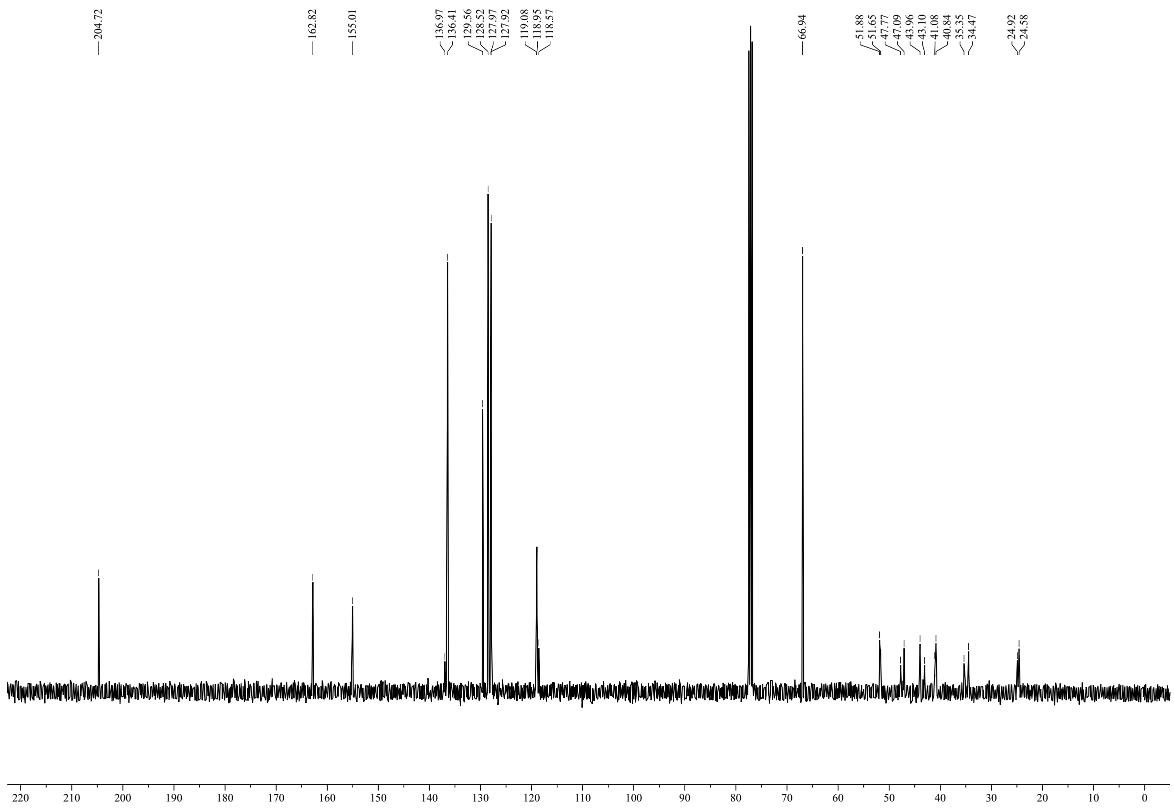

Figure S5: ^{1}H NMR (400 MHz, $CDCl_3$, top) and ^{13}C NMR (101 MHz, $CDCl_3$, bottom) spectra of **3a'** recorded at 298 K.

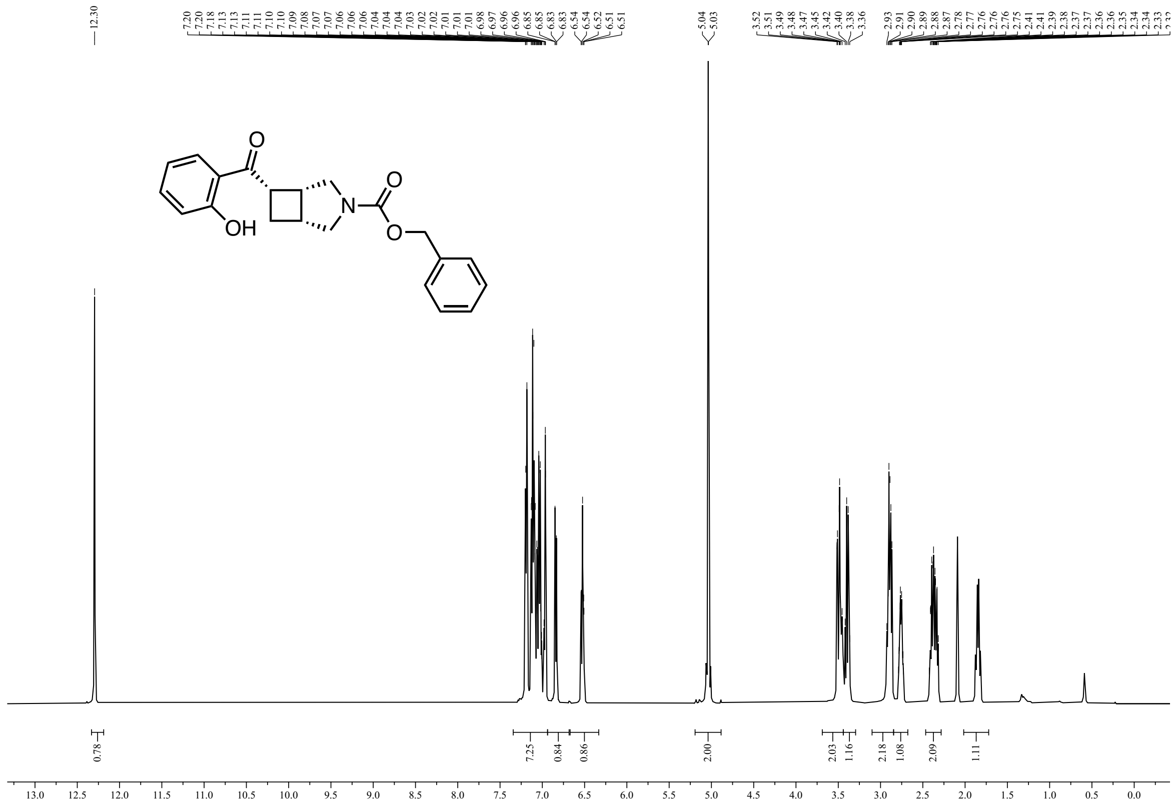

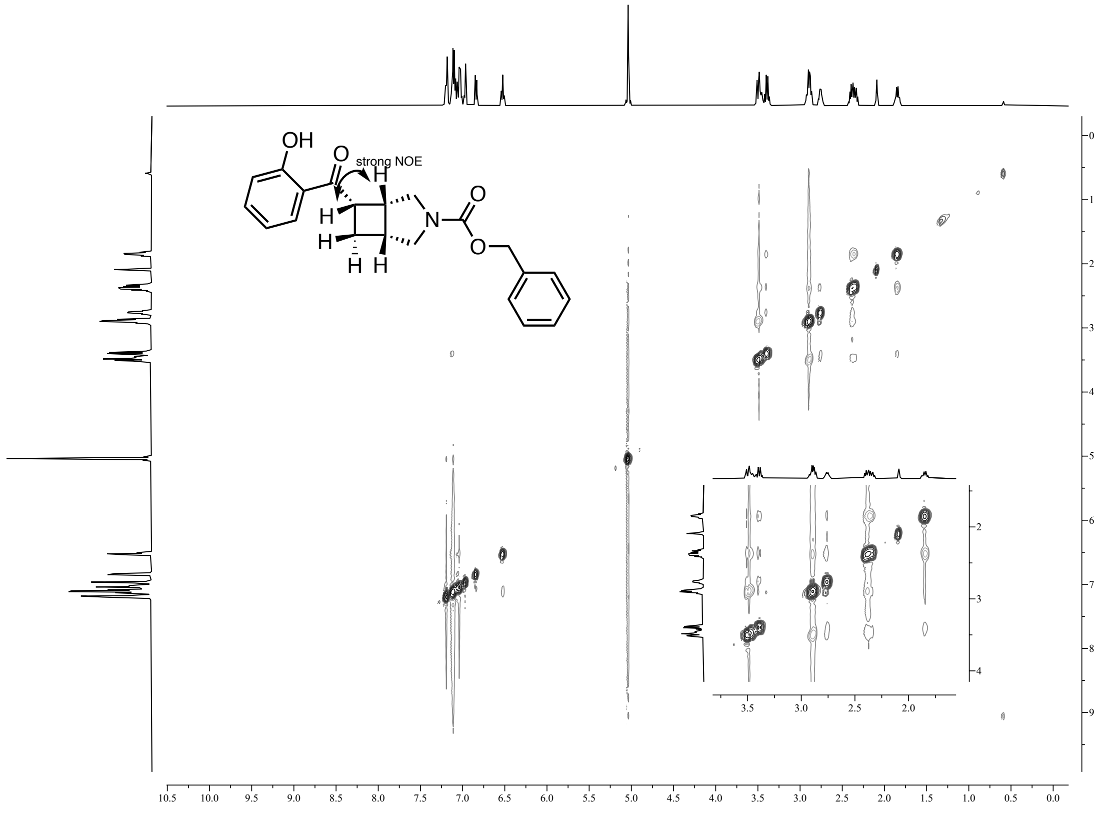

Figure S6: ^{1}H NMR (500 MHz, d8-toluene, top) and ^{1}H-^{1}H NOESY NMR (500 MHz, d8-toluene, bottom) spectrum of **3a’** recorded at 373 K.

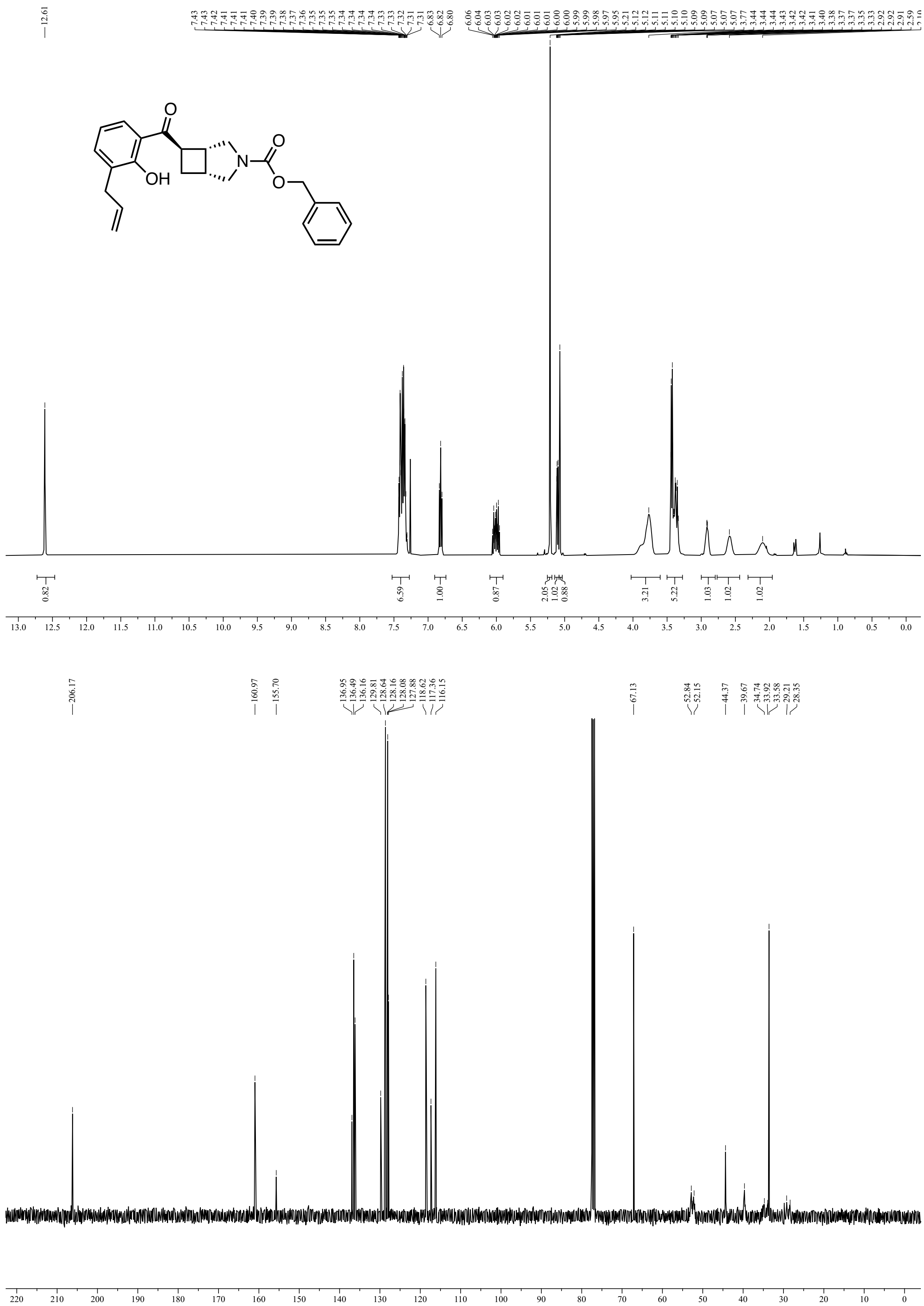

Figure S7: ^{1}H NMR (400 MHz, $CDCl_3$, top) and ^{13}C NMR (101 MHz, $CDCl_3$, bottom) spectra of **3b** recorded at 298 K.

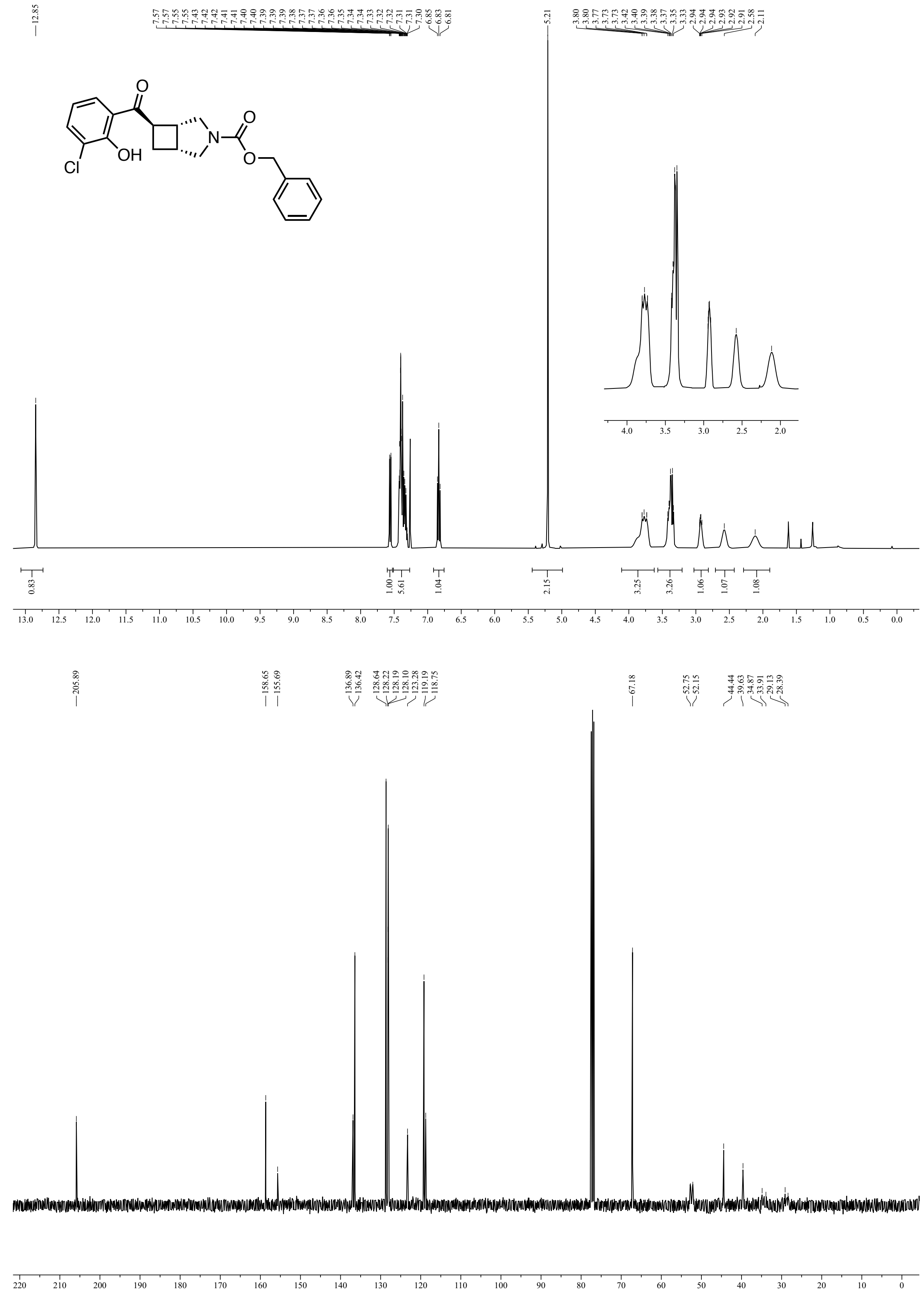

Figure S8: ^{1}H NMR (400 MHz, $CDCl_3$, top) and ^{13}C NMR (101 MHz, $CDCl_3$, bottom) spectra of **3c** recorded at 298 K.

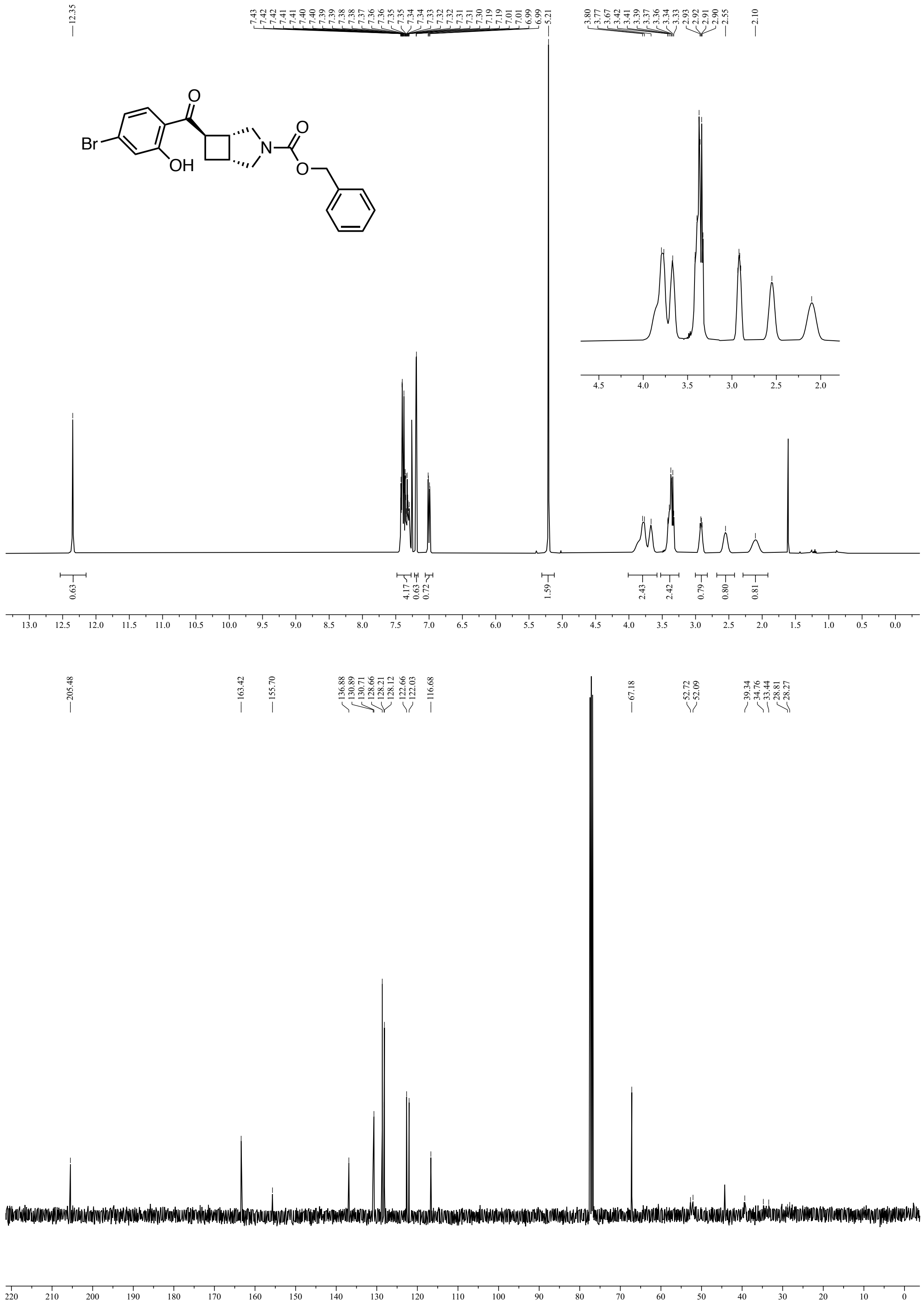

Figure S9: ^{1}H NMR (400 MHz, $CDCl_3$, top) and ^{13}C NMR (101 MHz, $CDCl_3$, bottom) spectra of **3d** recorded at 298 K.

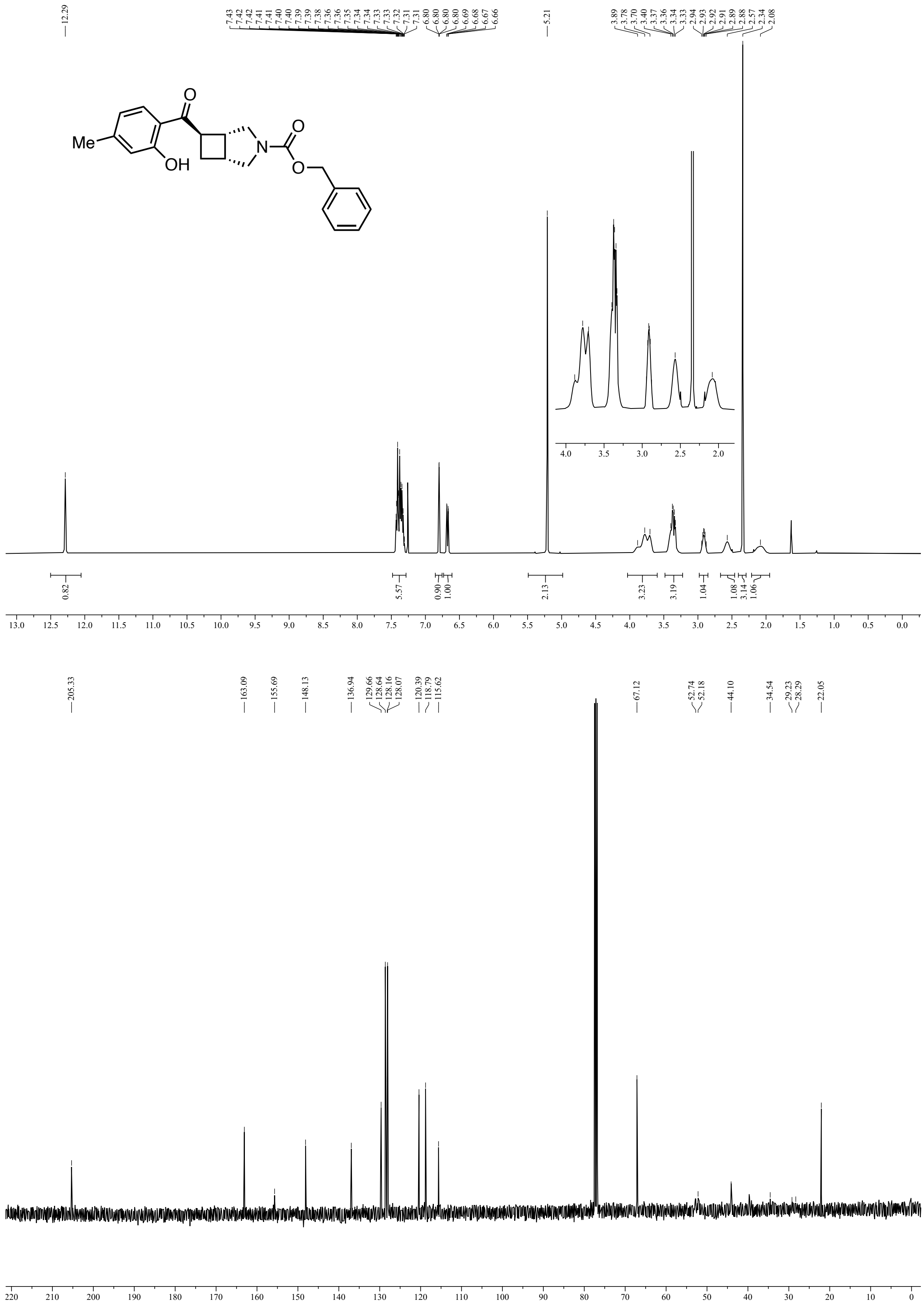

Figure S10: ^{1}H NMR (400 MHz, $CDCl_3$, top) and ^{13}C NMR (101 MHz, $CDCl_3$, bottom) spectra of **3e** recorded at 298 K.

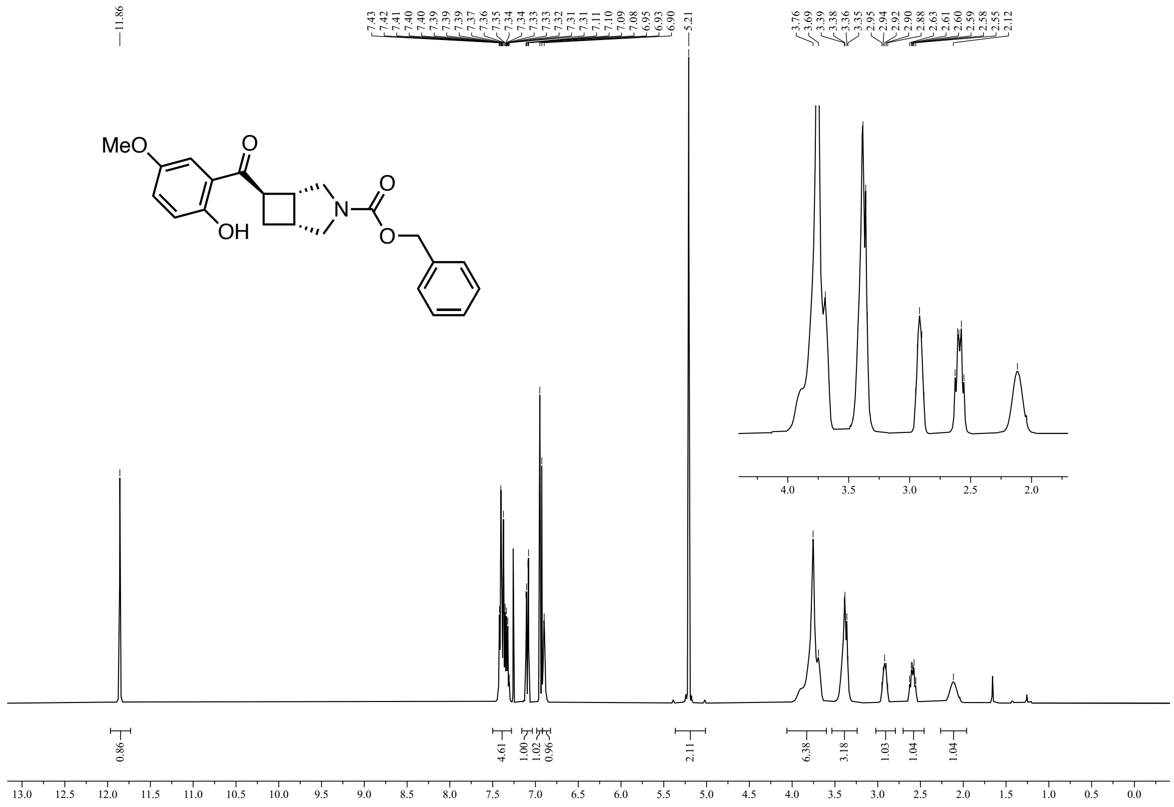

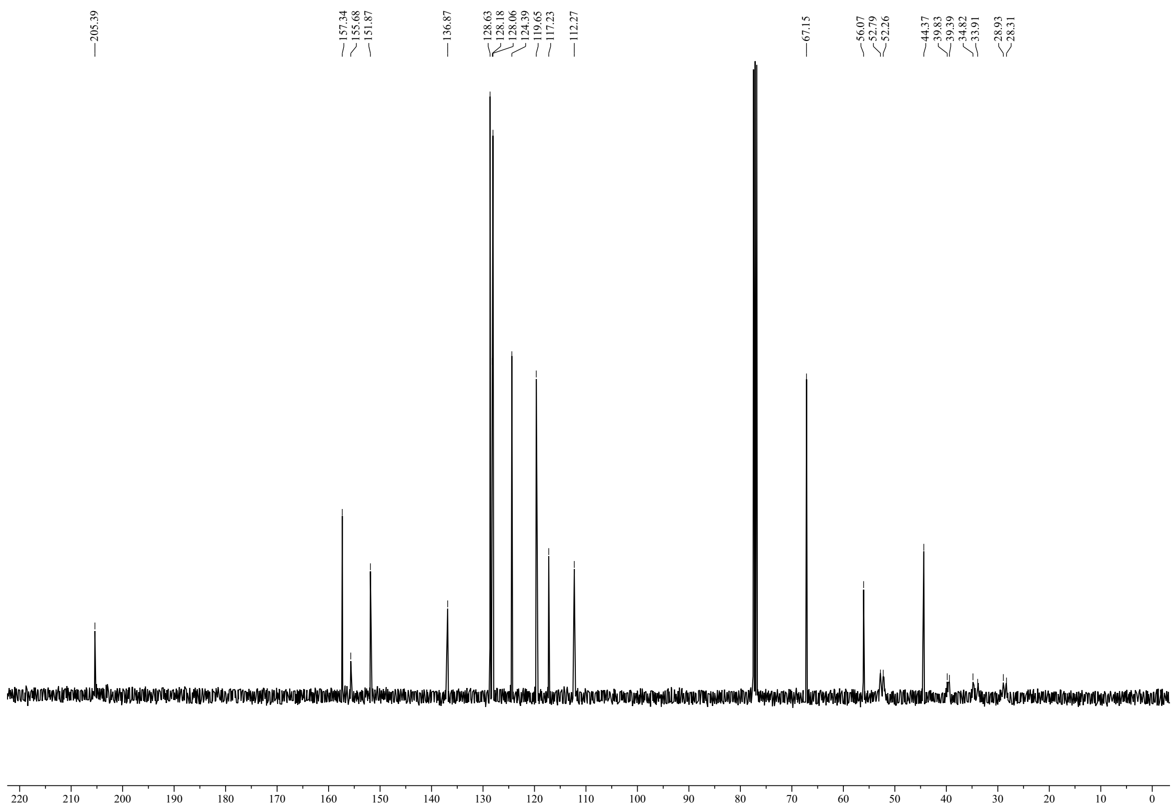

Figure S11: ^{1}H NMR (400 MHz, $CDCl_3$, top) and ^{13}C NMR (101 MHz, $CDCl_3$, bottom) spectra of **3f** recorded at 298 K.

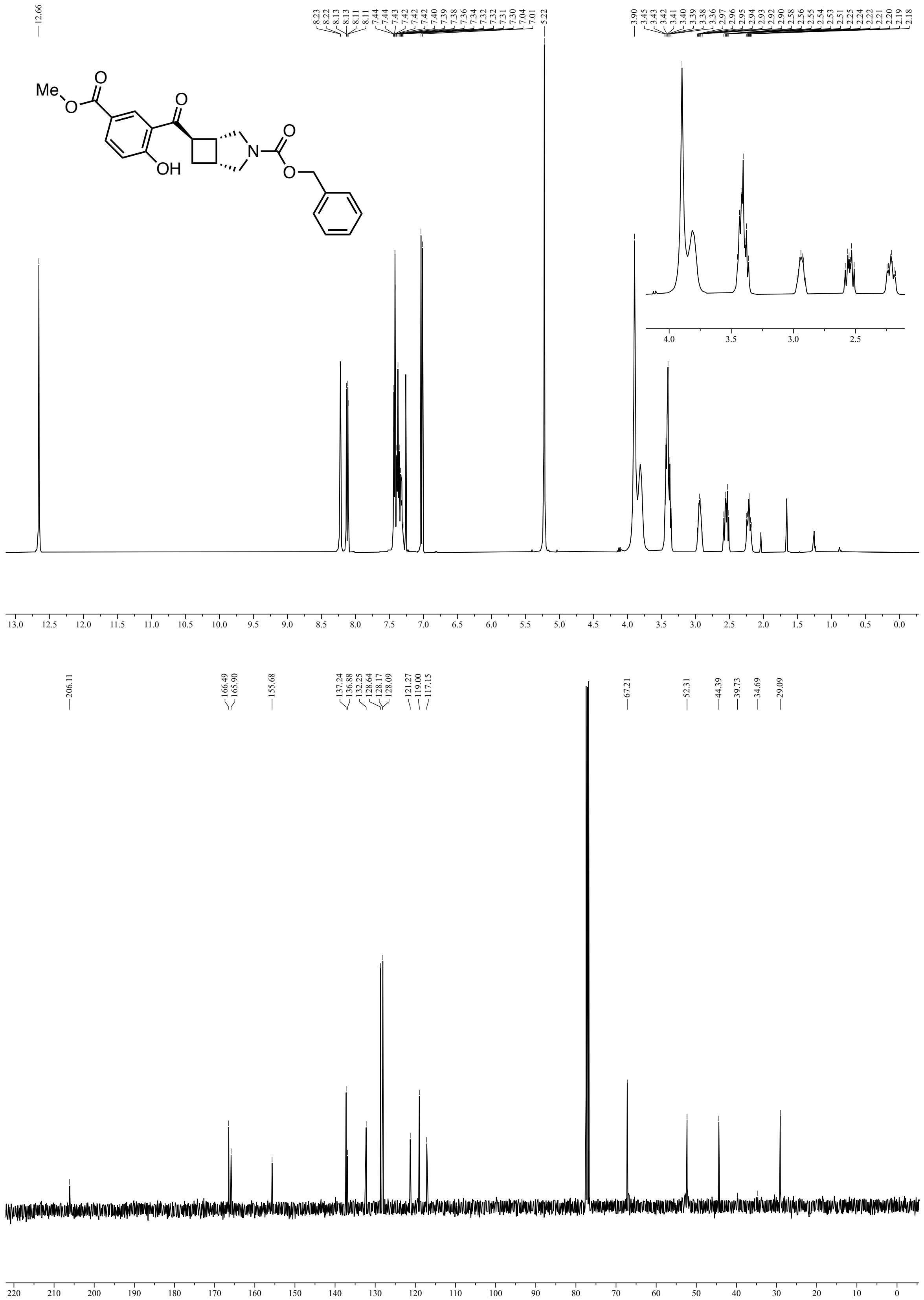

Figure S12: ^{1}H NMR (400 MHz, $CDCl_3$, top) and ^{13}C NMR (101 MHz, $CDCl_3$, bottom) spectra of **3g** recorded at 298 K.

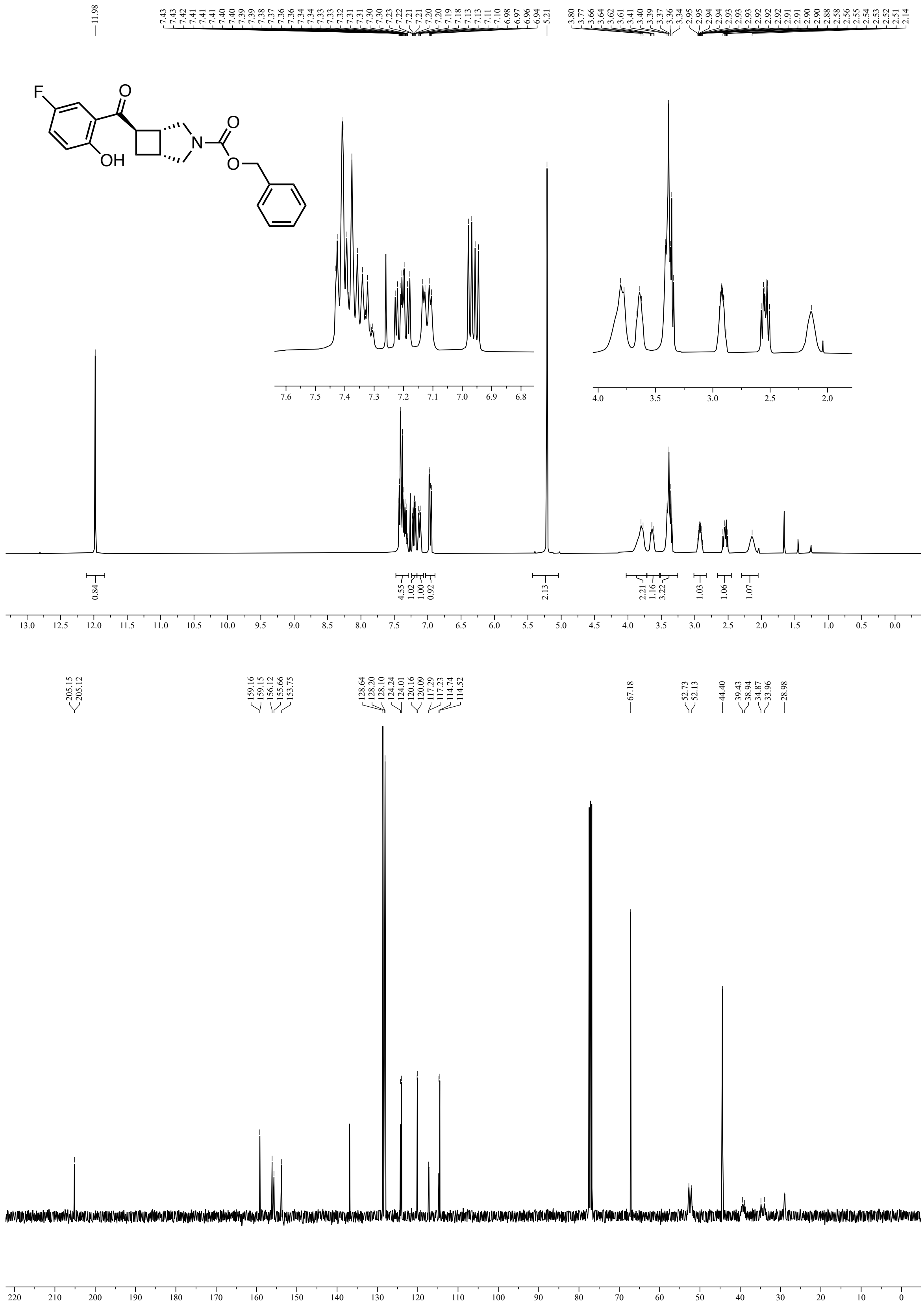

Figure S13: ^{1}H NMR (400 MHz, $CDCl_3$, top) and ^{13}C NMR (101 MHz, $CDCl_3$, bottom) spectra of **3h** recorded at 298 K.

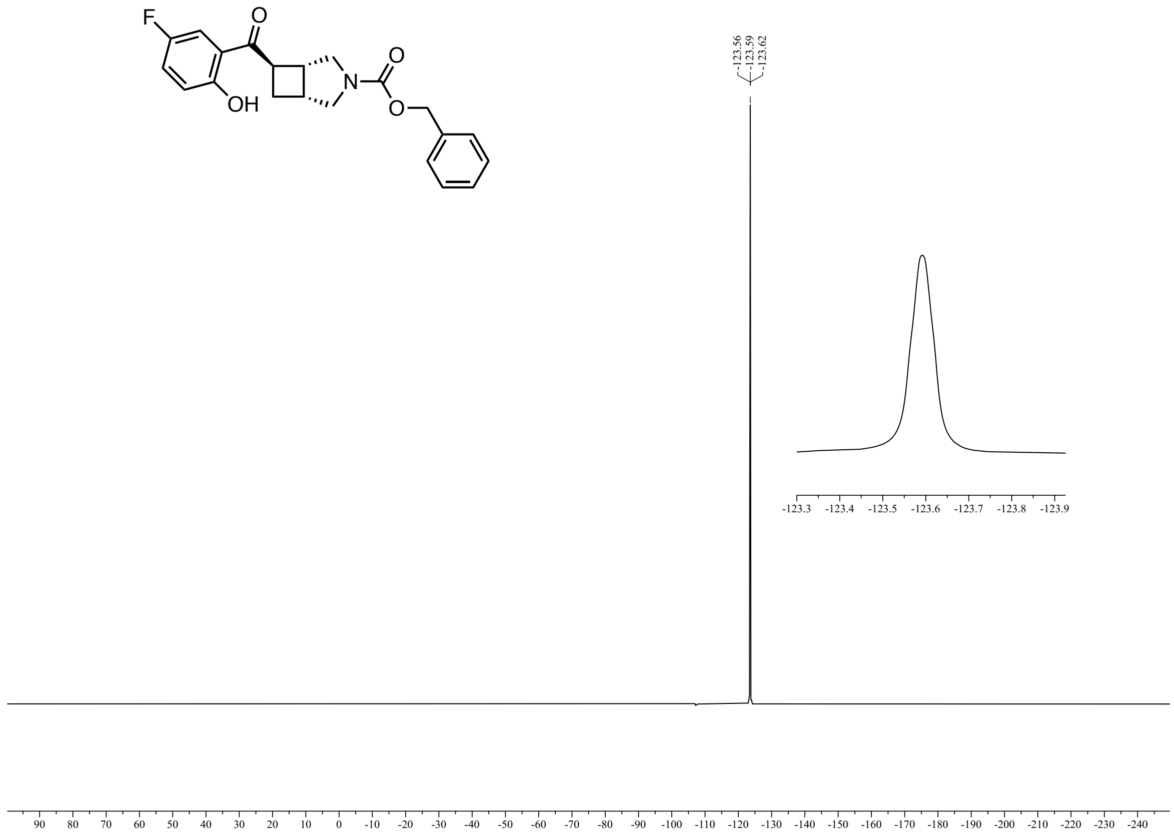

Figure S14: ^{19}F NMR (376 MHz, $CDCl_3$) spectrum of **3h** recorded at 298 K.

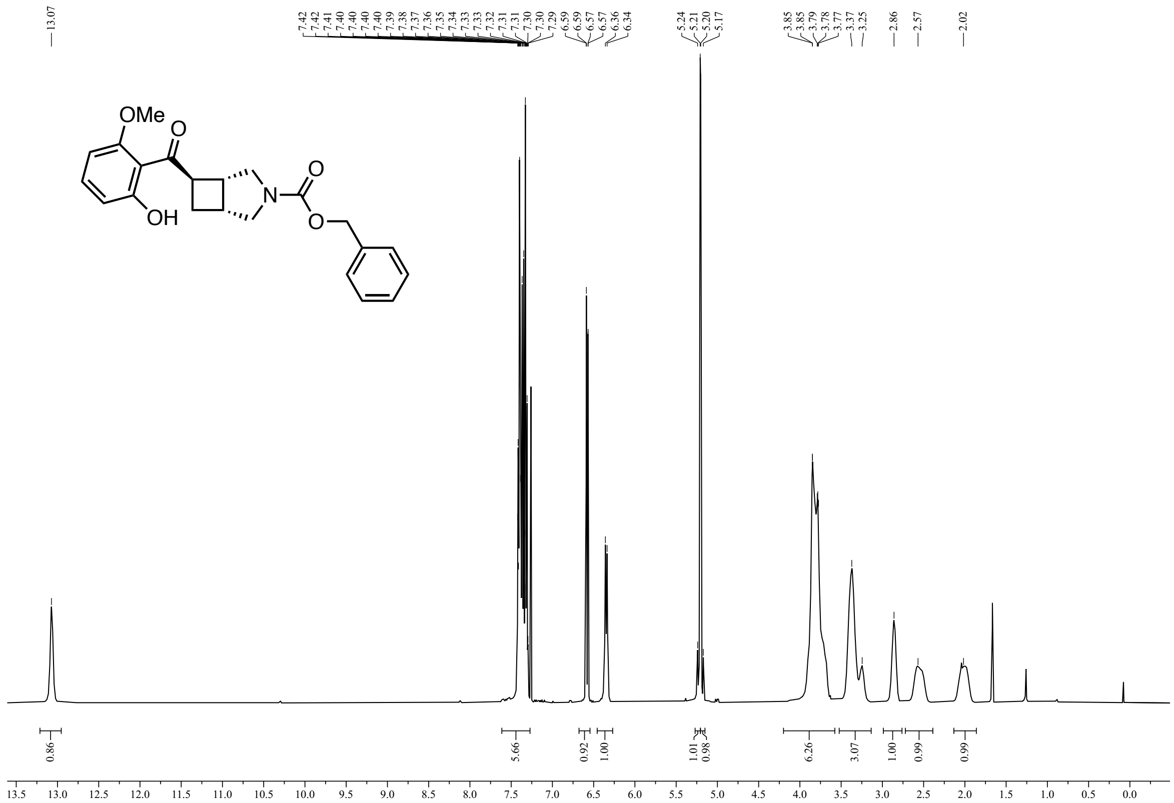

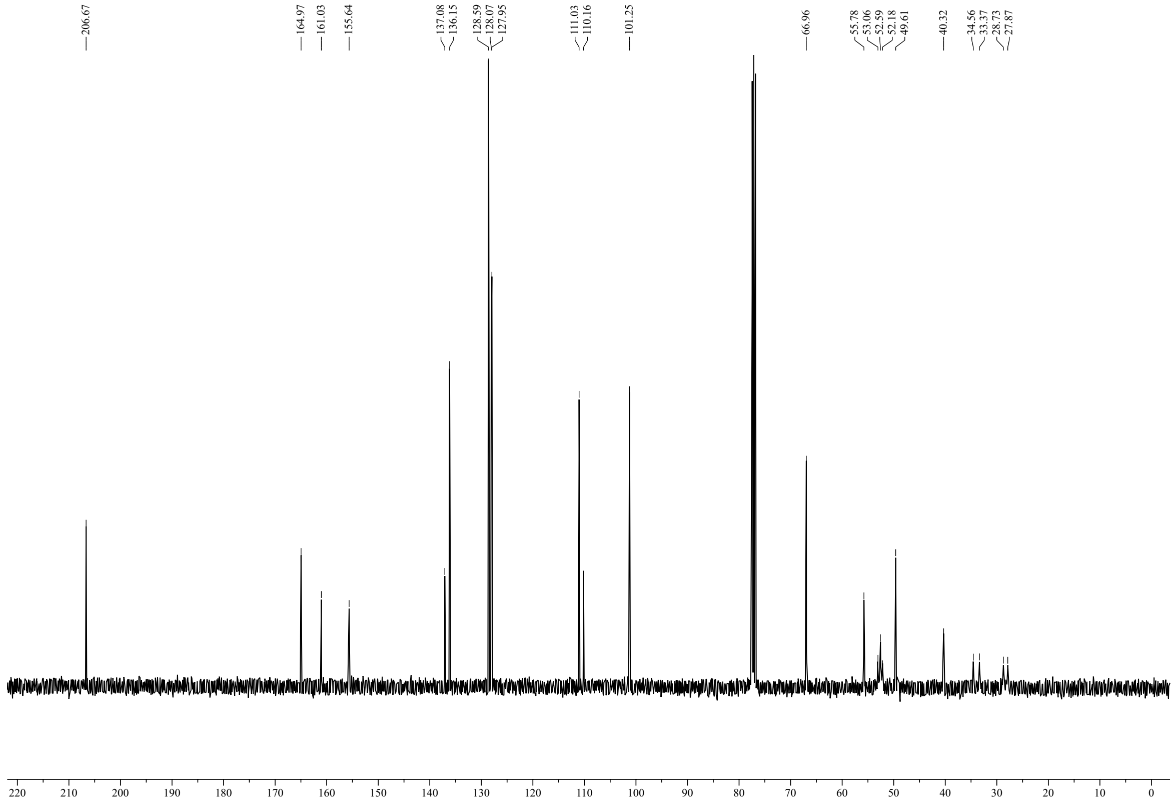

Figure S15: ^1H NMR (400 MHz, $CDCl_3$, top) and ^{13}C NMR (101 MHz, $CDCl_3$, bottom) spectra of **3i** recorded at 298 K.

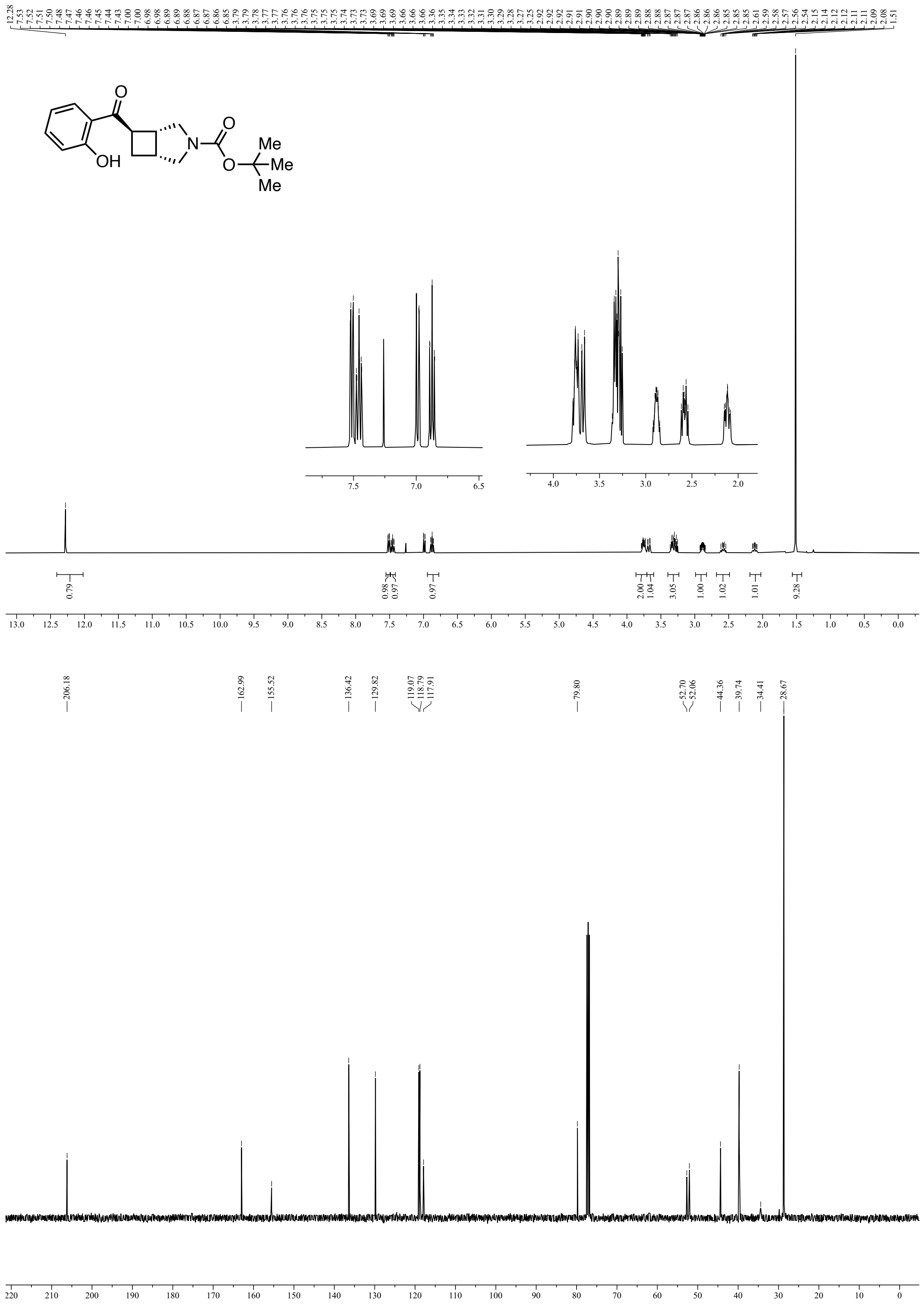

Figure S16: ^{1}H NMR (400 MHz, $CDCl_3$, top) and ^{13}C NMR (101 MHz, $CDCl_3$, bottom) spectra of **4a** recorded at 298 K.

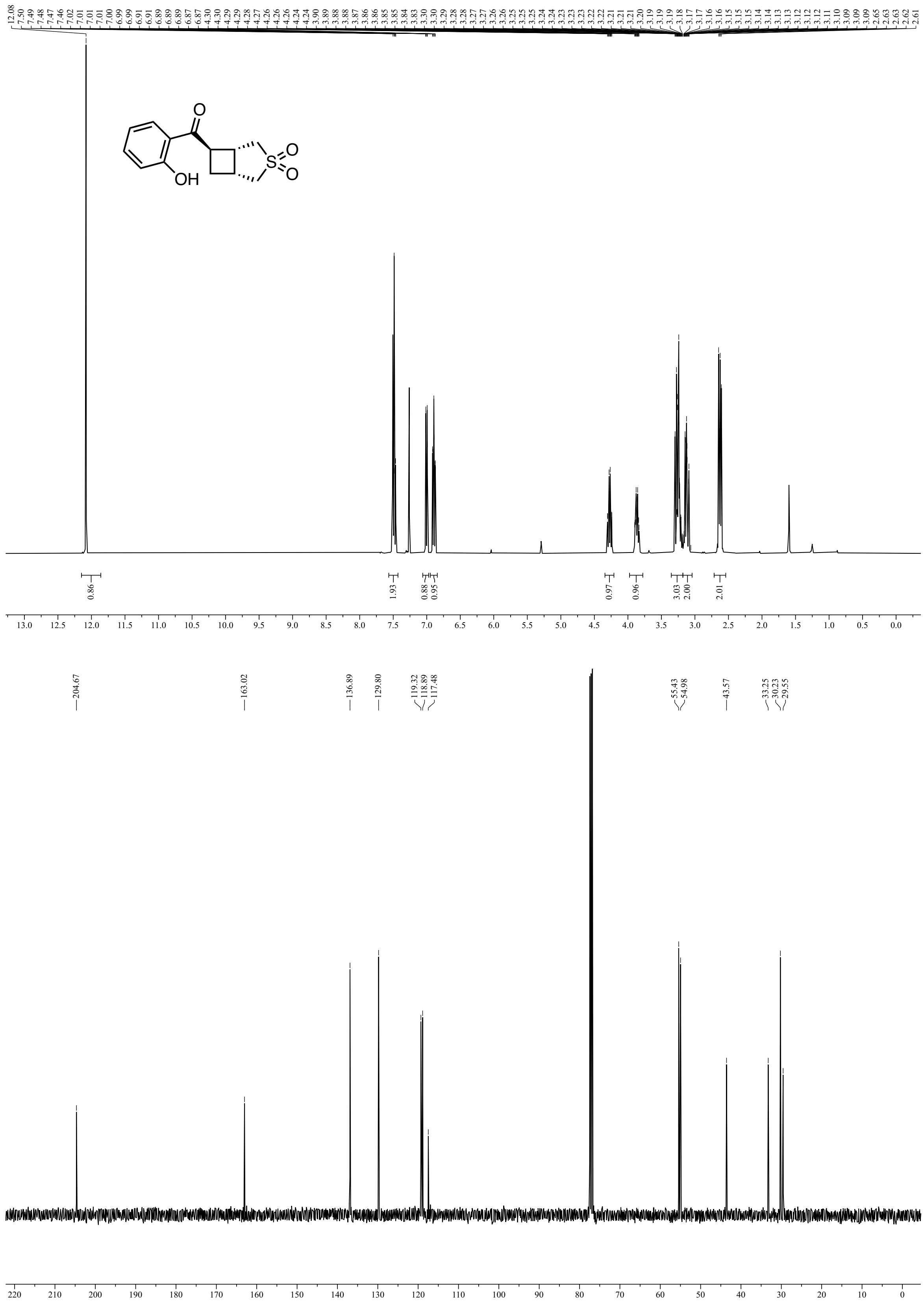

Figure S17: ^1H NMR (400 MHz, $CDCl_3$, top) and ^{13}C NMR (101 MHz, $CDCl_3$, bottom) spectra of **4b** recorded at 298 K.

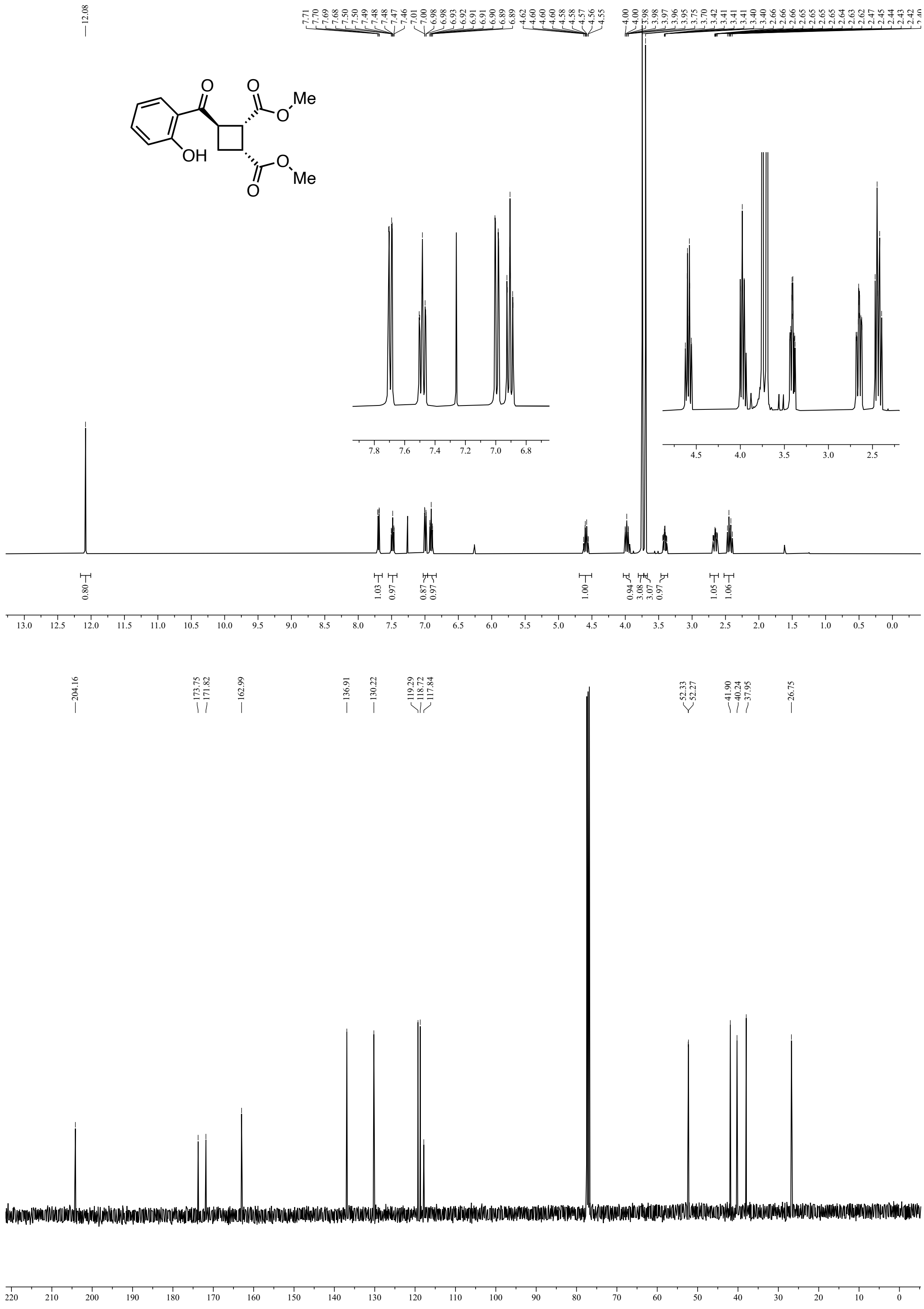

Figure S18: ^{1}H NMR (400 MHz, $CDCl_3$, top) and ^{13}C NMR (101 MHz, $CDCl_3$, bottom) spectra of **4c** (contaminated with 3% unreacted **2c**) recorded at 298 K.

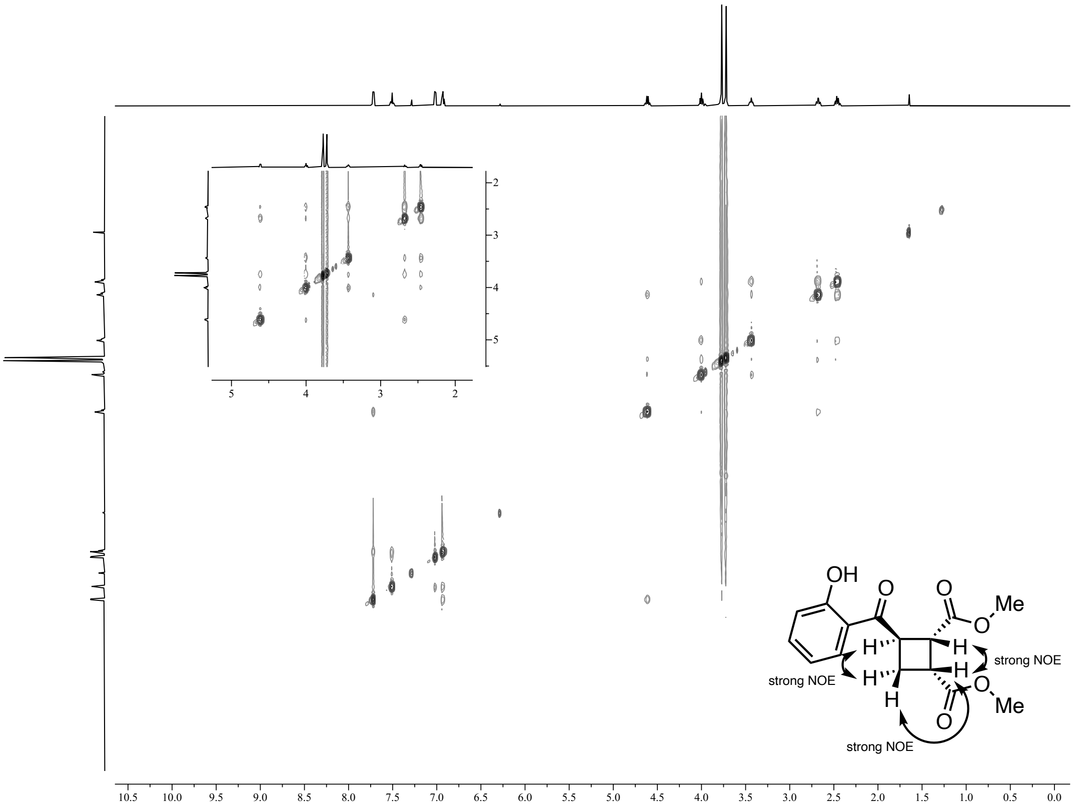

Figure S19: ^{1}H-^{1}H NOESY NMR (600 MHz, $CDCl_3$) spectrum of **4c** recorded at 298 K.

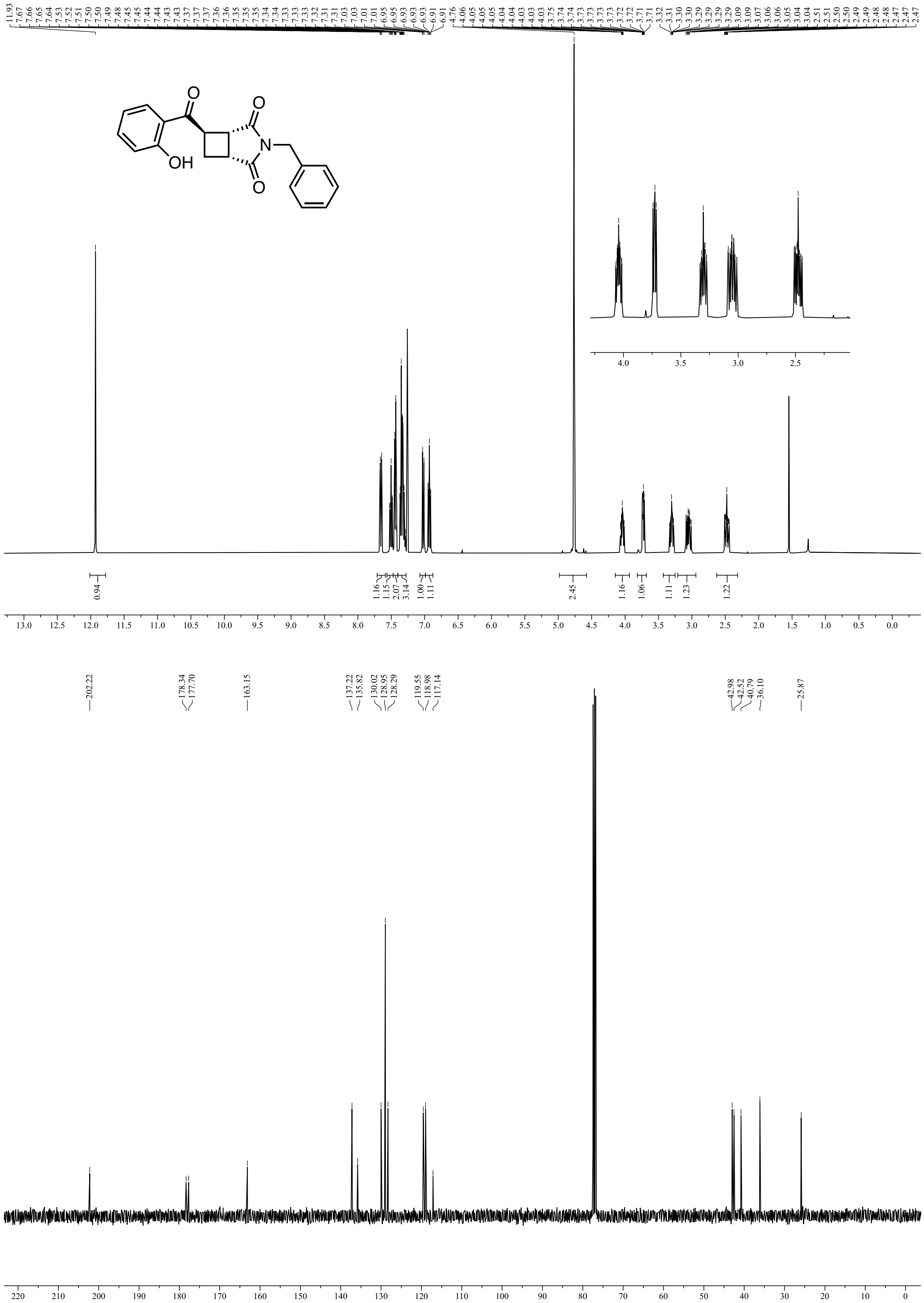

Figure S20: ^{1}H NMR (400 MHz, $CDCl_3$, top) and ^{13}C NMR (101 MHz, $CDCl_3$, bottom) spectra of **4d** recorded at 298 K.

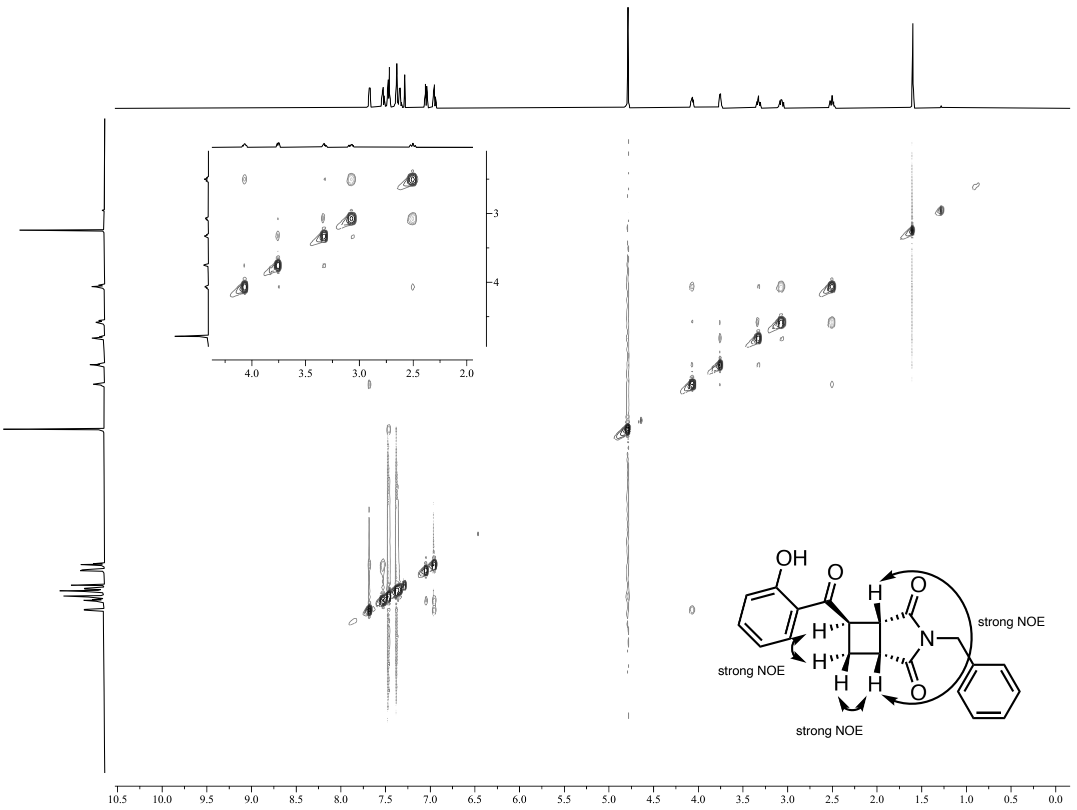

Figure S21: ^{1}H-^{1}H NOESY NMR (600 MHz, $CDCl_3$) spectrum of **4e** recorded at 298 K.

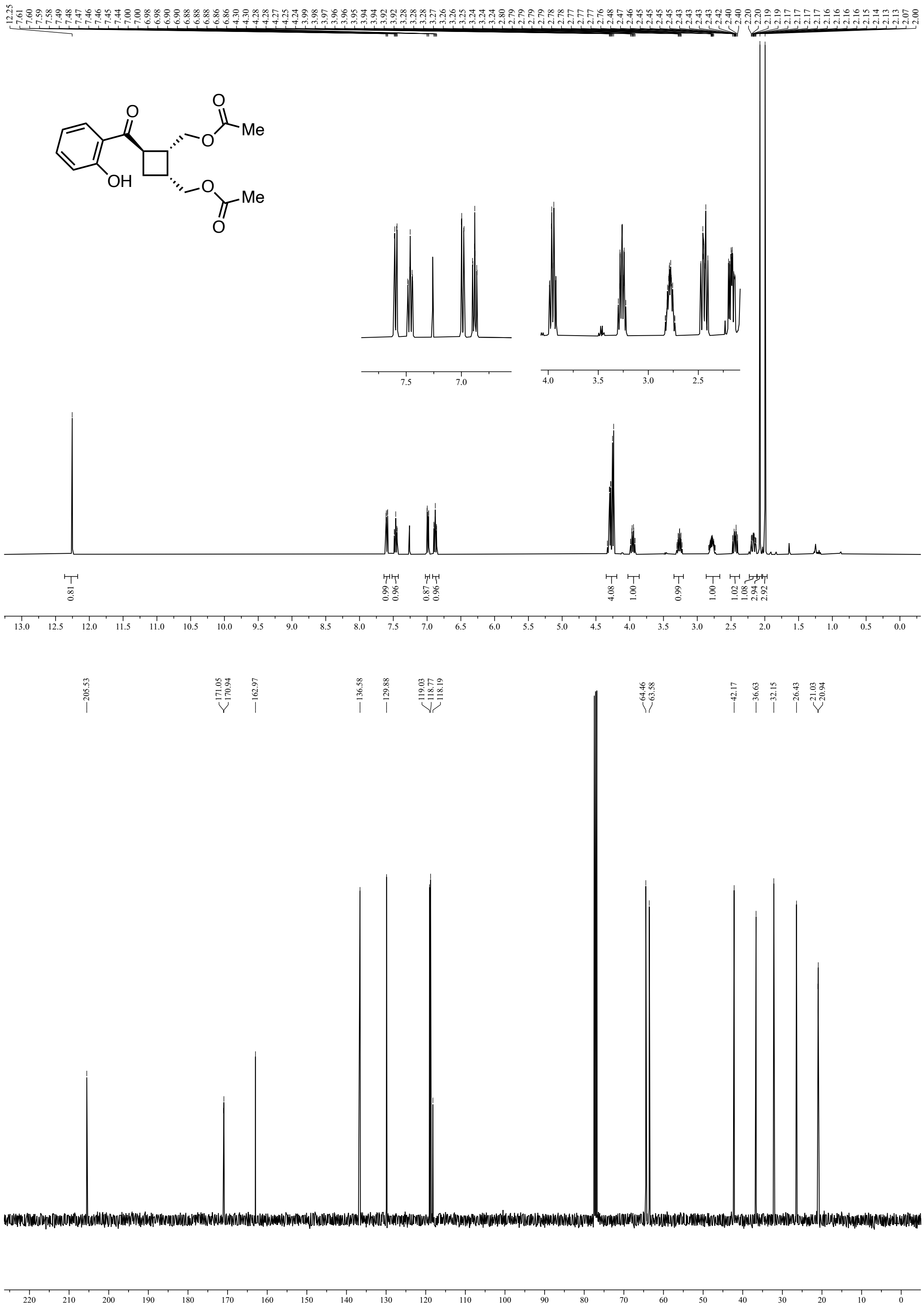

Figure S22: ^{1}H NMR (400 MHz, $CDCl_3$, top) and ^{13}C NMR (101 MHz, $CDCl_3$, bottom) spectra of **4e** recorded at 298 K.

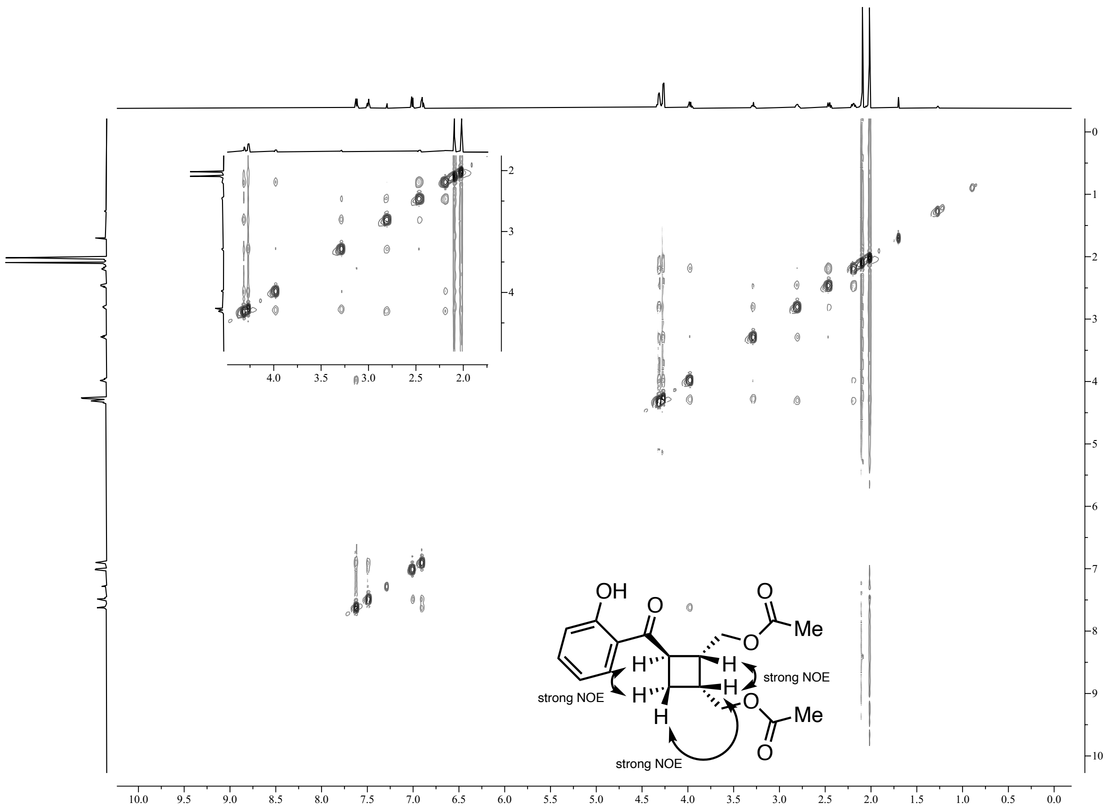

Figure S23: ^{1}H-^{1}H NOESY NMR (600 MHz, $CDCl_3$) spectrum of **4e** recorded at 298 K.

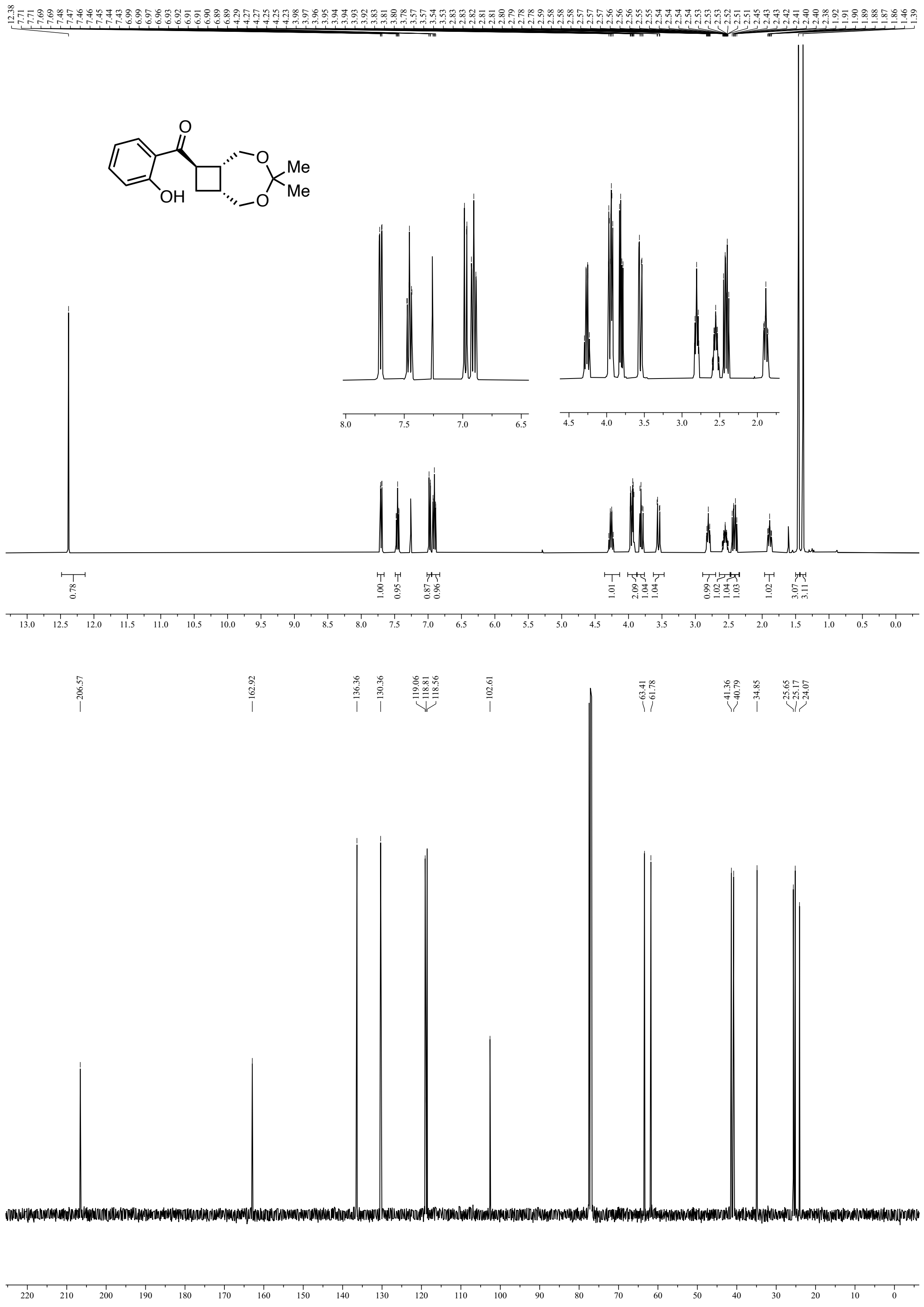

Figure S24: ^{1}H NMR (400 MHz, $CDCl_3$, top) and ^{13}C NMR (101 MHz, $CDCl_3$, bottom) spectra of **4f** recorded at 298 K.

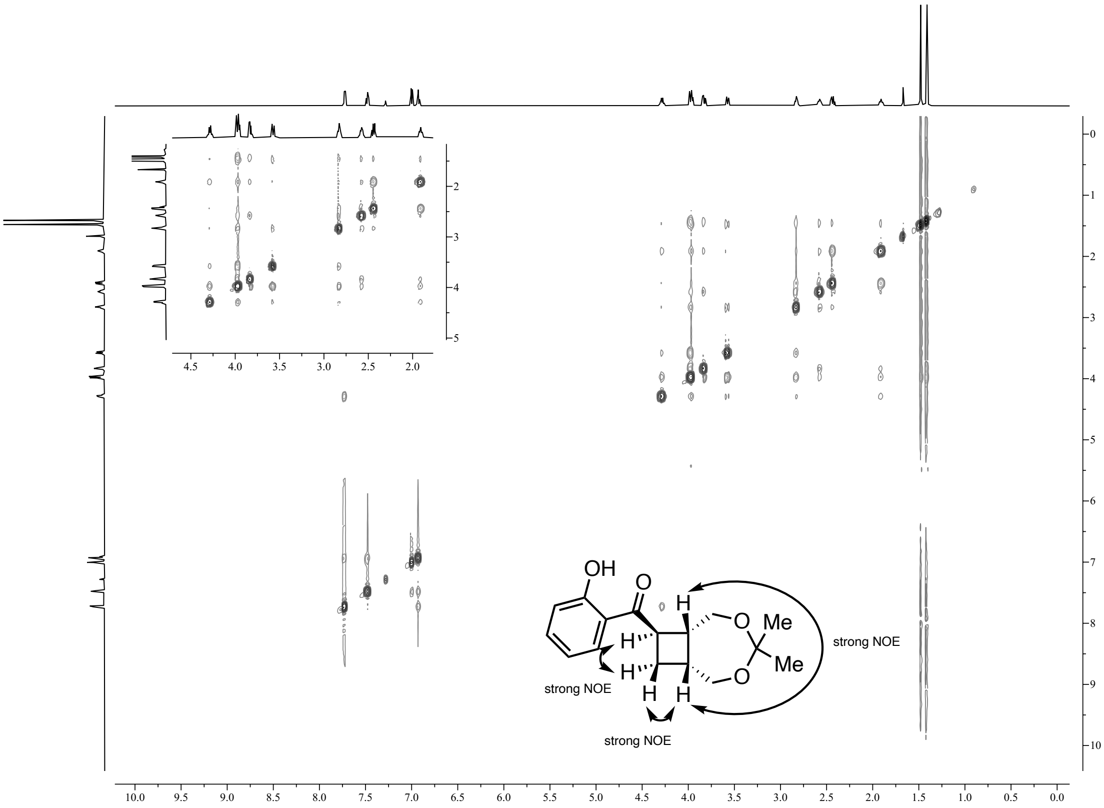

Figure S25: ^{1}H-^{1}H NOESY NMR (600 MHz, $CDCl_3$) spectrum of **4f** recorded at 298 K.

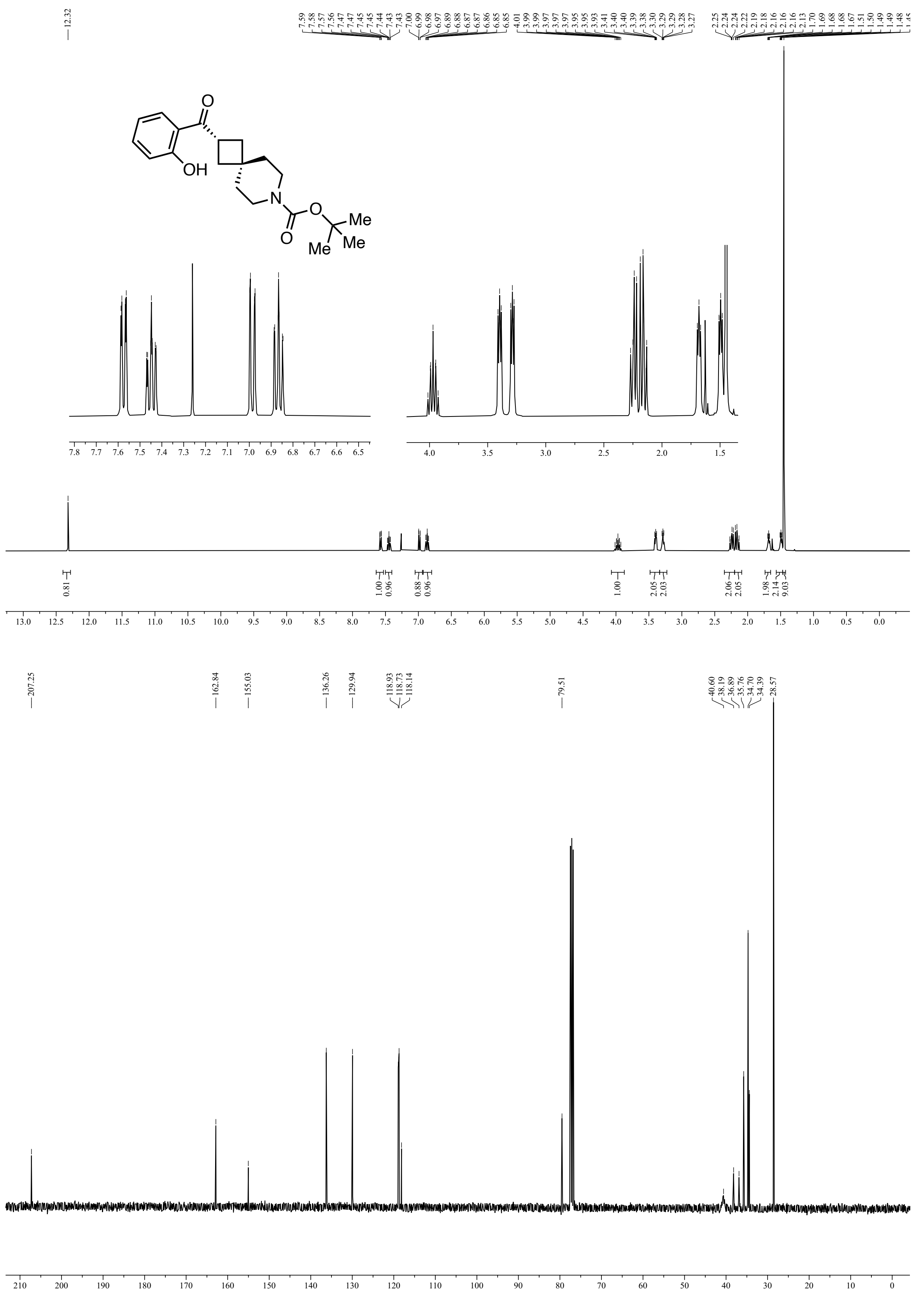

Figure S26: ^1H NMR (400 MHz, $CDCl_3$, top) and ^{13}C NMR (101 MHz, $CDCl_3$, bottom) spectra of **6a** recorded at 298 K.

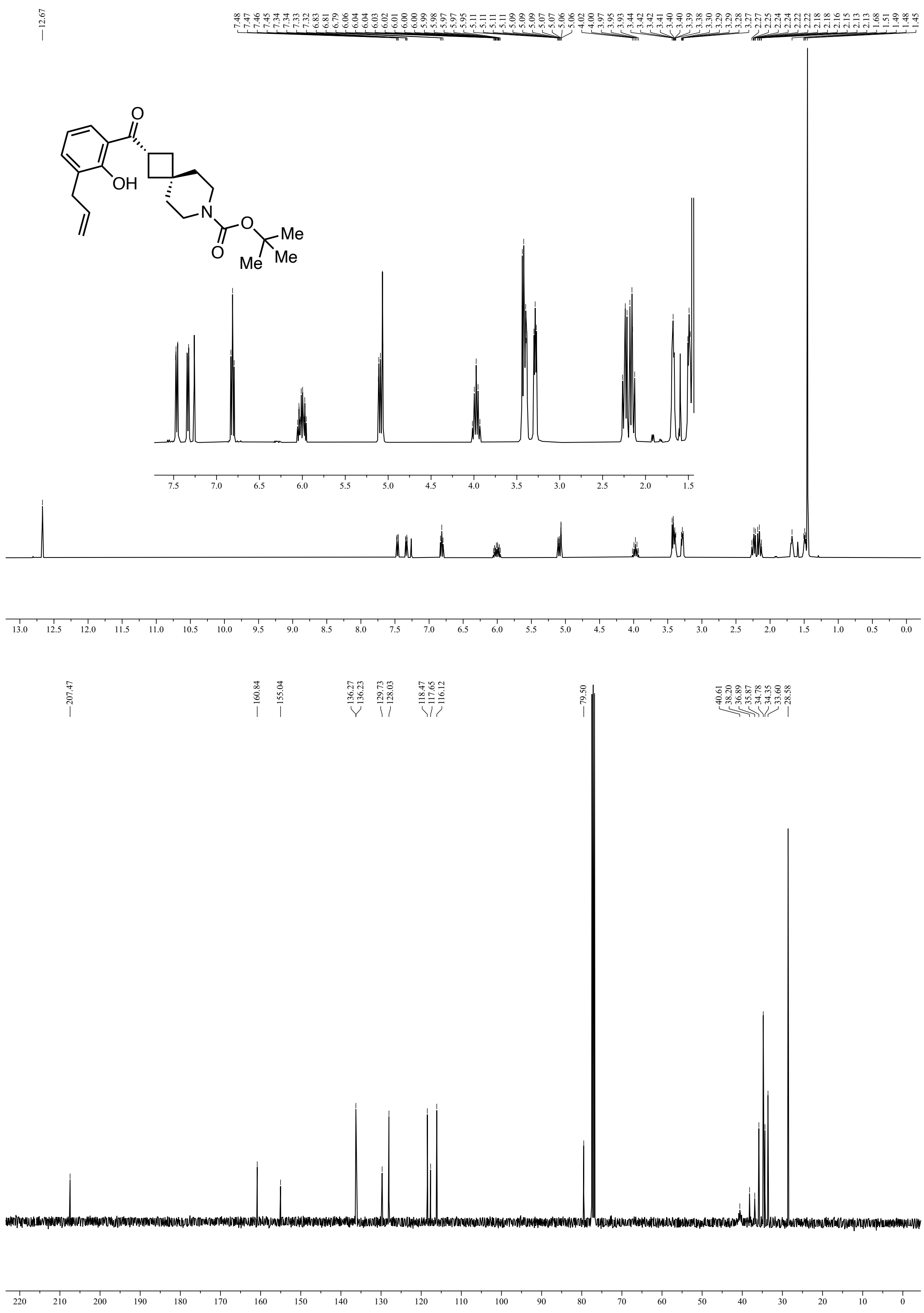

Figure S27: ^{1}H NMR (400 MHz, $CDCl_3$, top) and ^{13}C NMR (101 MHz, $CDCl_3$, bottom) spectra of **6b** recorded at 298 K.

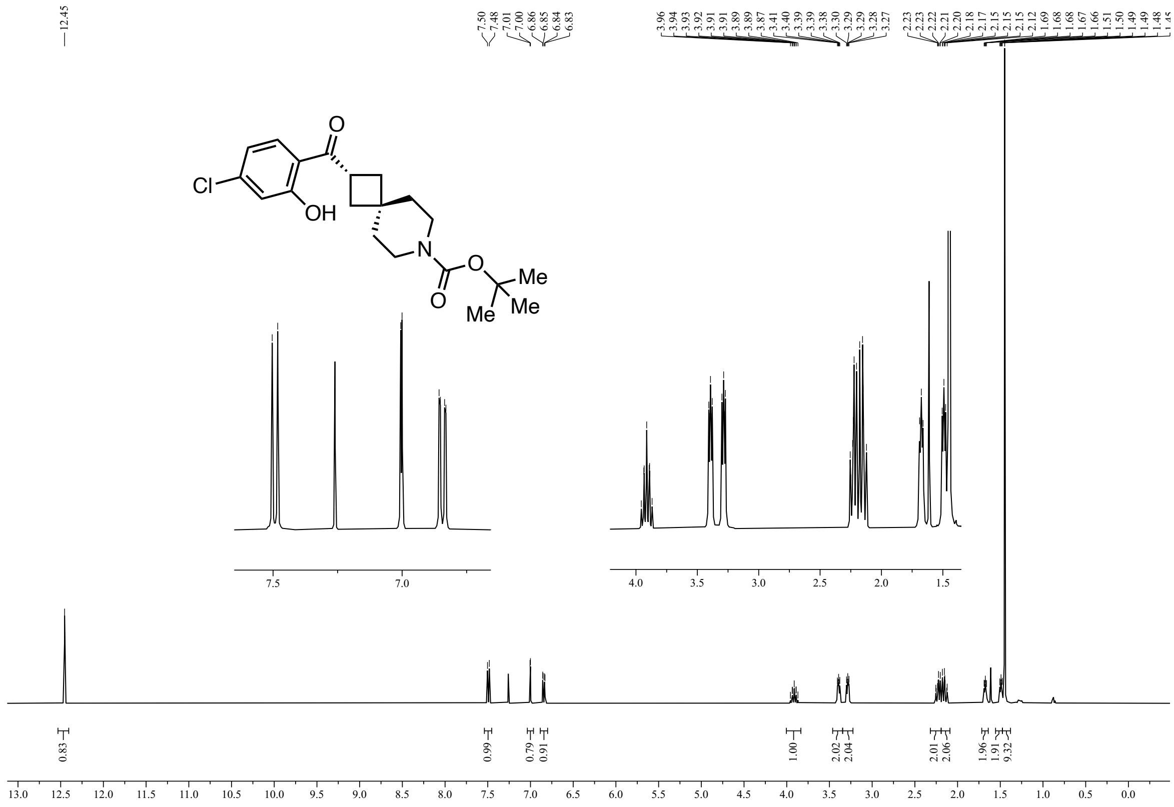

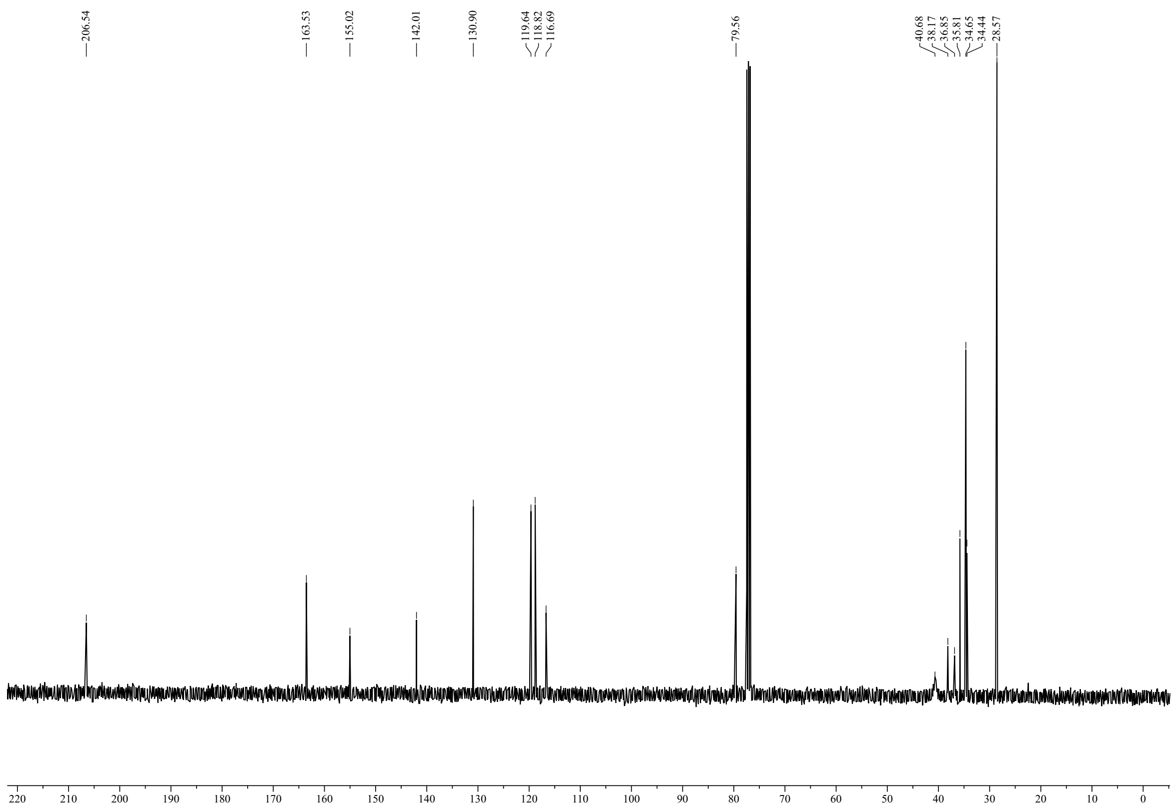

Figure S28: ^{1}H NMR (400 MHz, $CDCl_3$, top) and ^{13}C NMR (101 MHz, $CDCl_3$, bottom) spectra of **6c** recorded at 298 K.

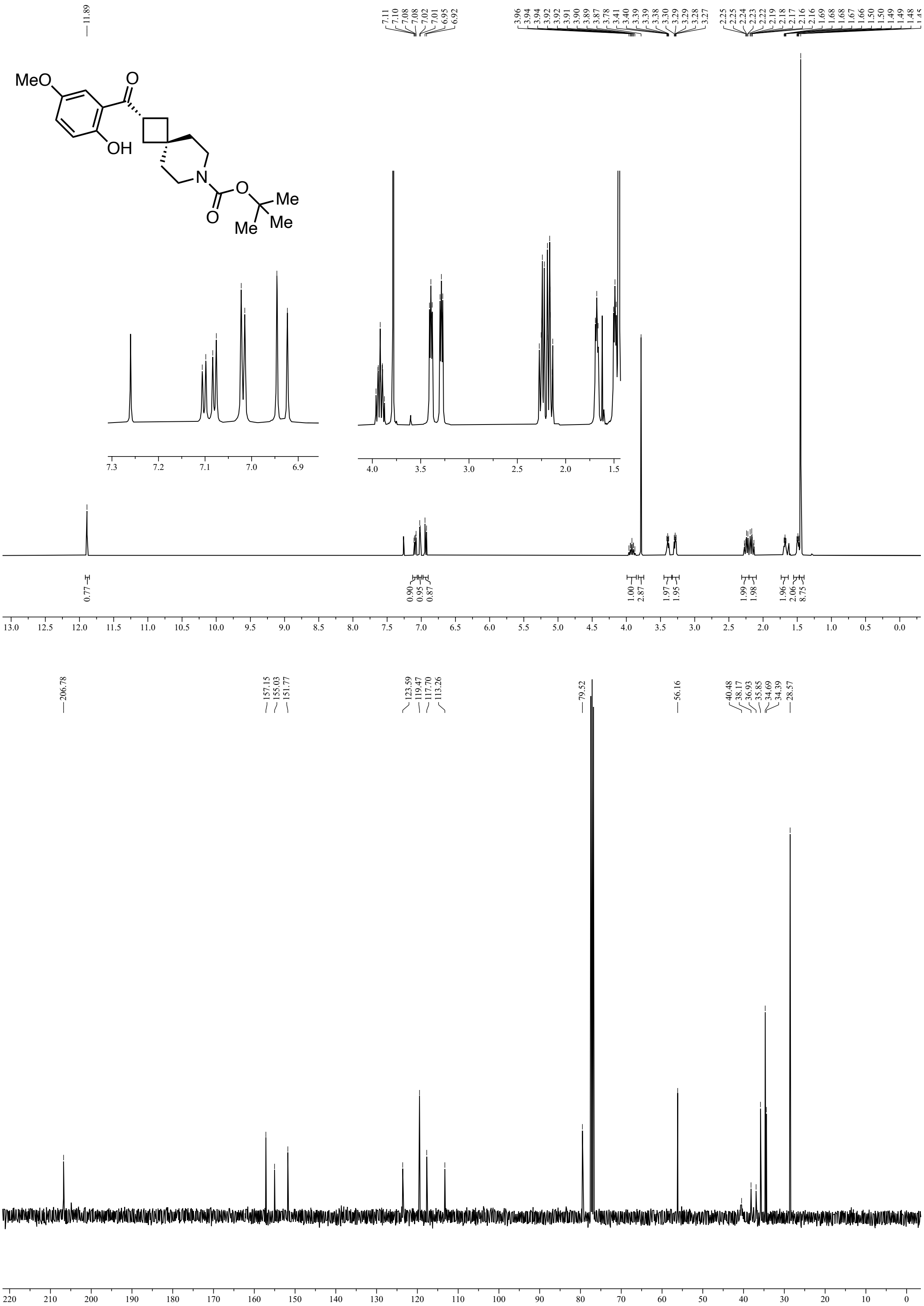

Figure S29: ^{1}H NMR (400 MHz, $CDCl_3$, top) and ^{13}C NMR (101 MHz, $CDCl_3$, bottom) spectra of **6d** recorded at 298 K.

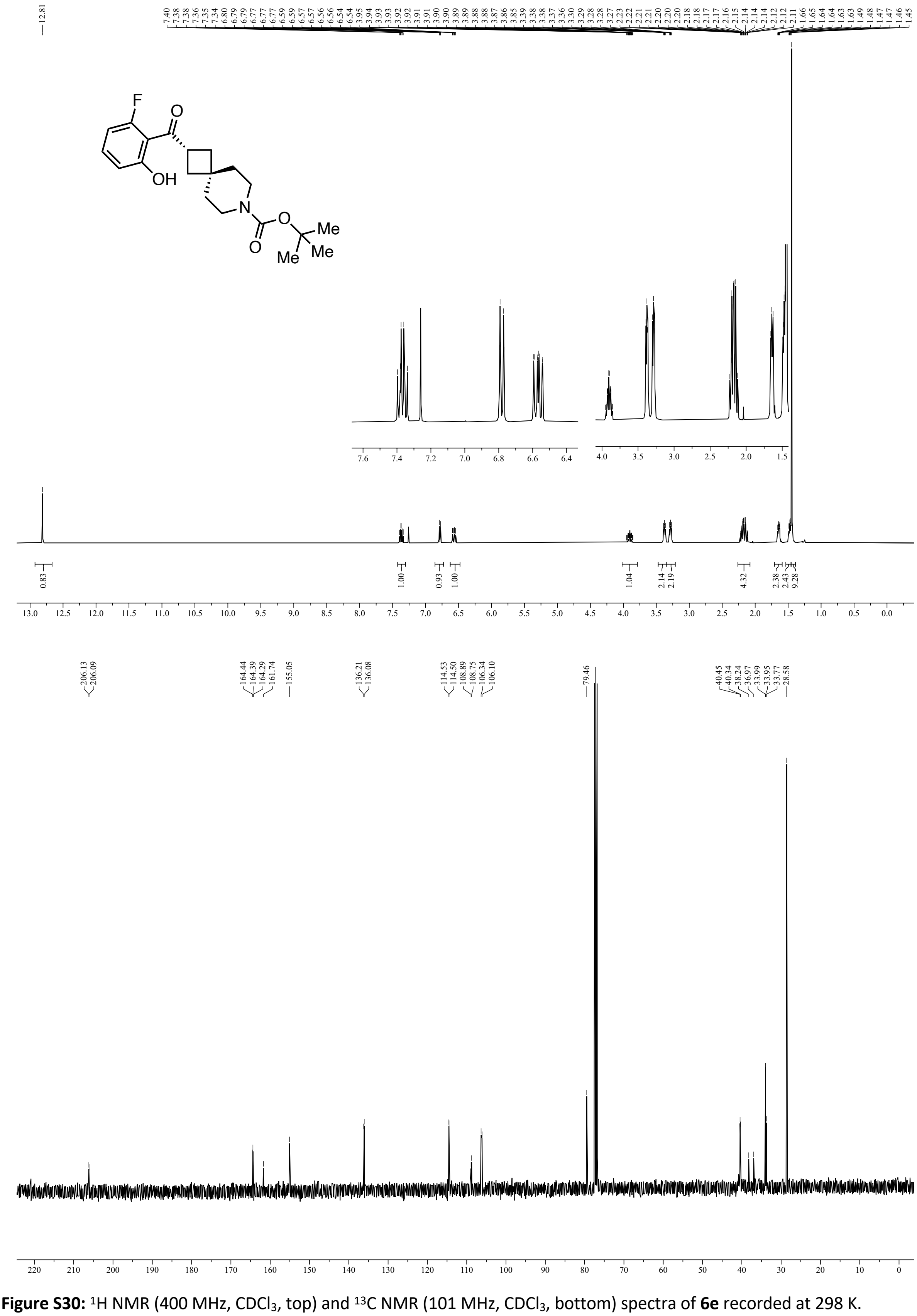

Figure S30: ^{1}H NMR (400 MHz, $CDCl_3$, top) and ^{13}C NMR (101 MHz, $CDCl_3$, bottom) spectra of **6e** recorded at 298 K.

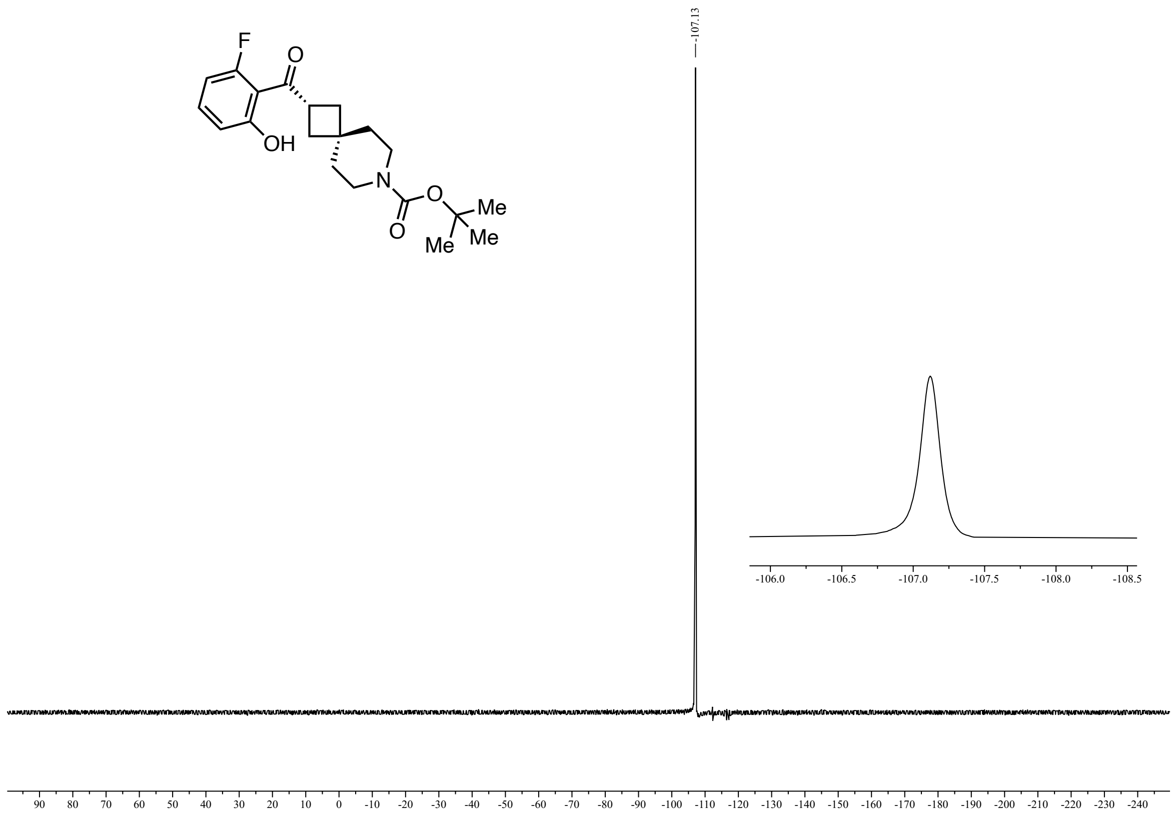

Figure S31: ^{19}F NMR (376 MHz, $CDCl_3$) spectrum of **6e** recorded at 298 K.

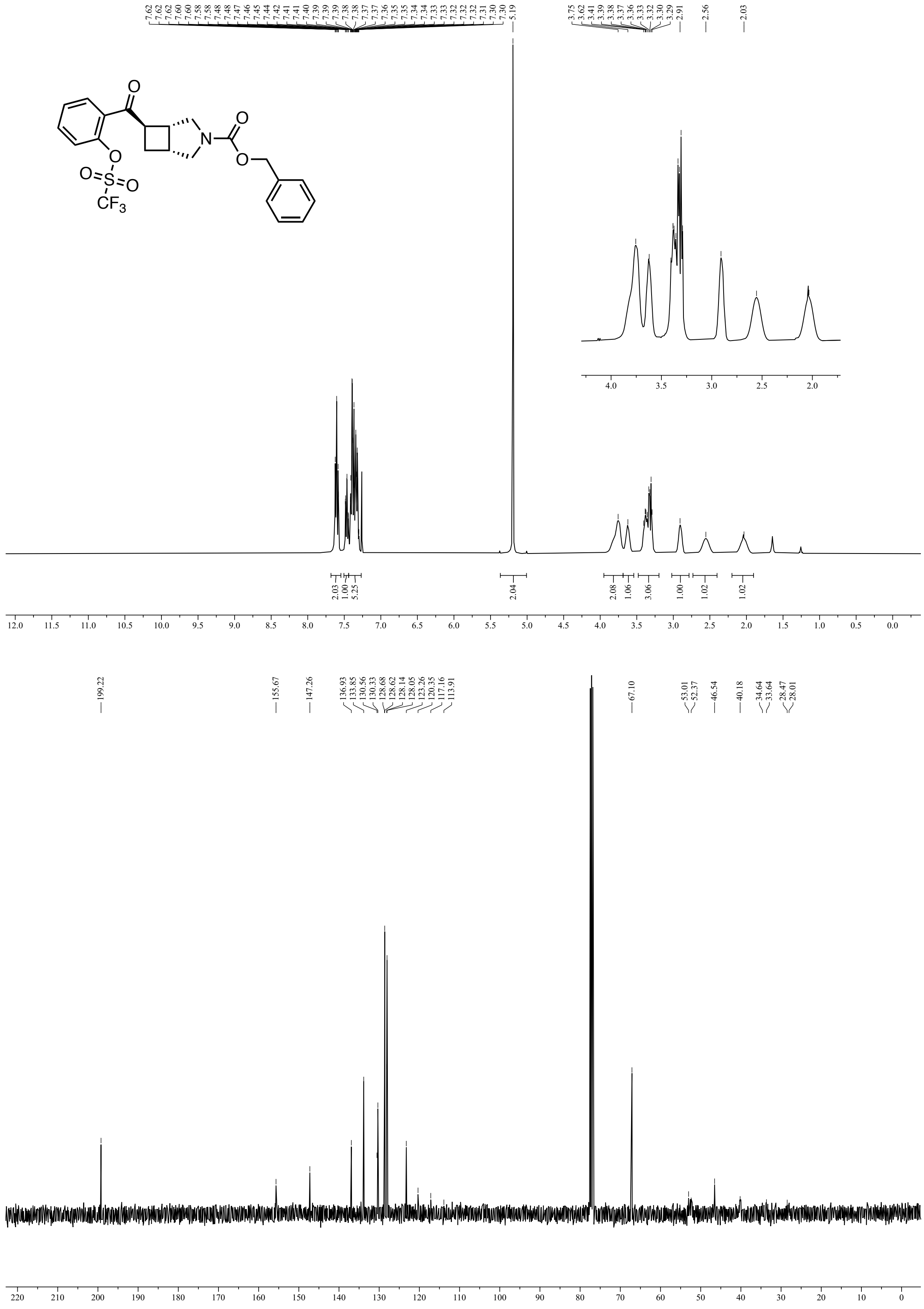

Figure S32: ^{1}H NMR (400 MHz, $CDCl_3$, top) and ^{13}C NMR (101 MHz, $CDCl_3$, bottom) spectra of **7a** recorded at 298 K.

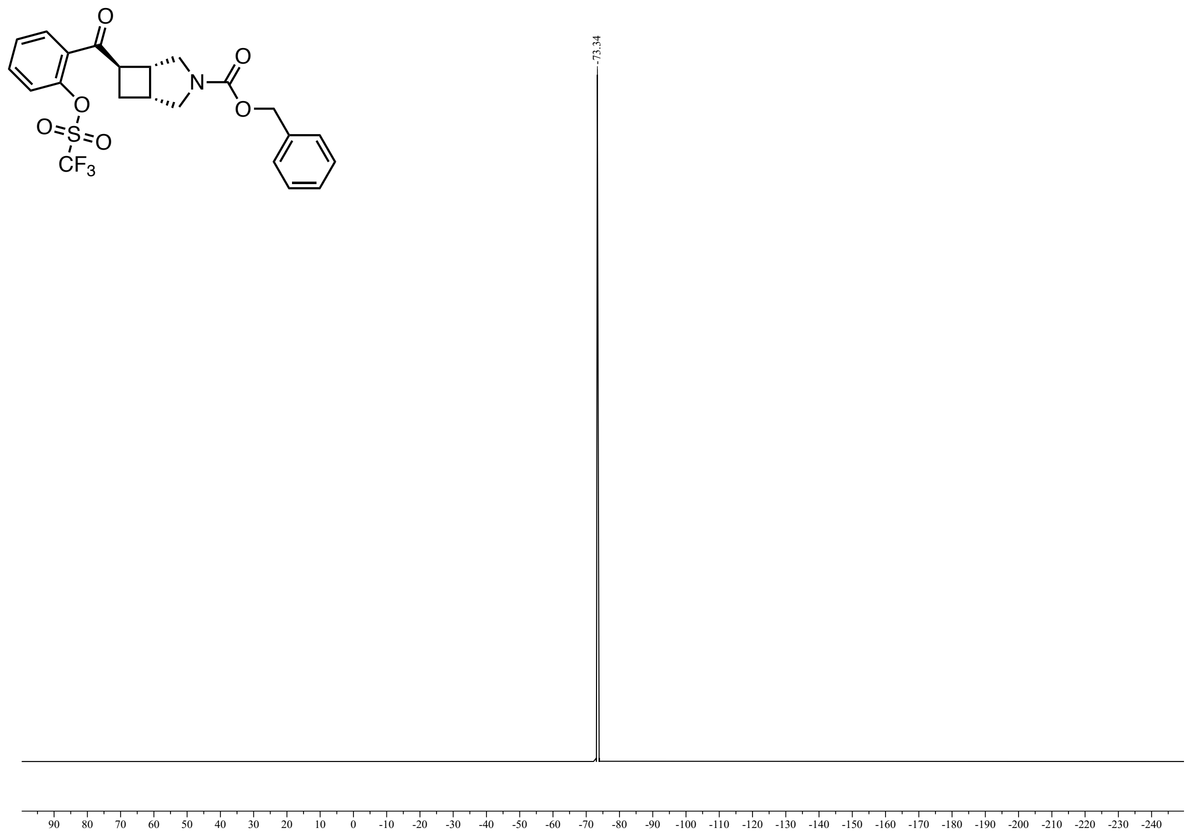

Figure S33: ^{19}F NMR (376 MHz, $CDCl_3$) spectrum of **7a** recorded at 298 K.

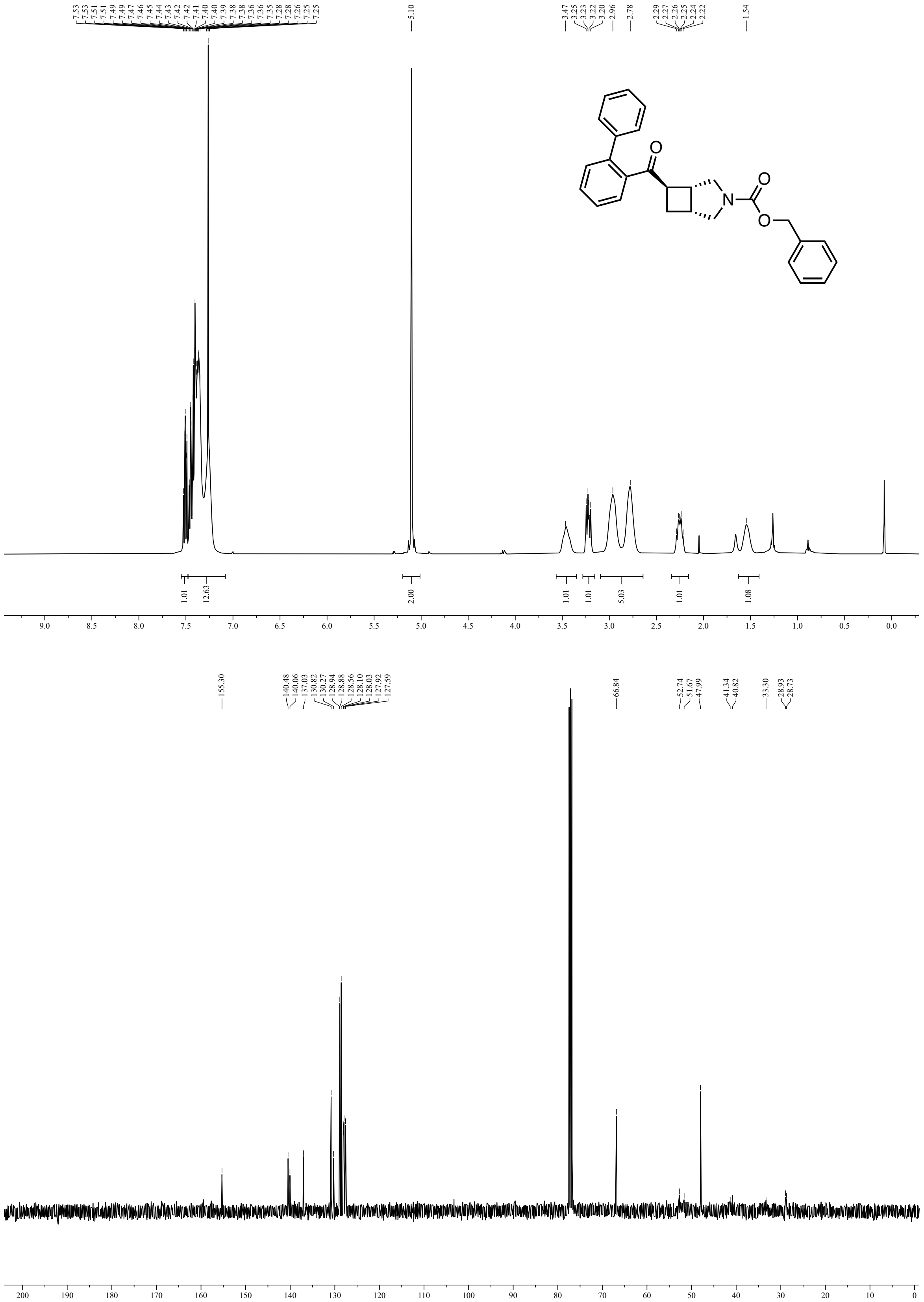

Figure S34: ^{1}H NMR (400 MHz, $CDCl_3$, top) and ^{13}C NMR (101 MHz, $CDCl_3$, bottom) spectra of **7b** recorded at 298 K.

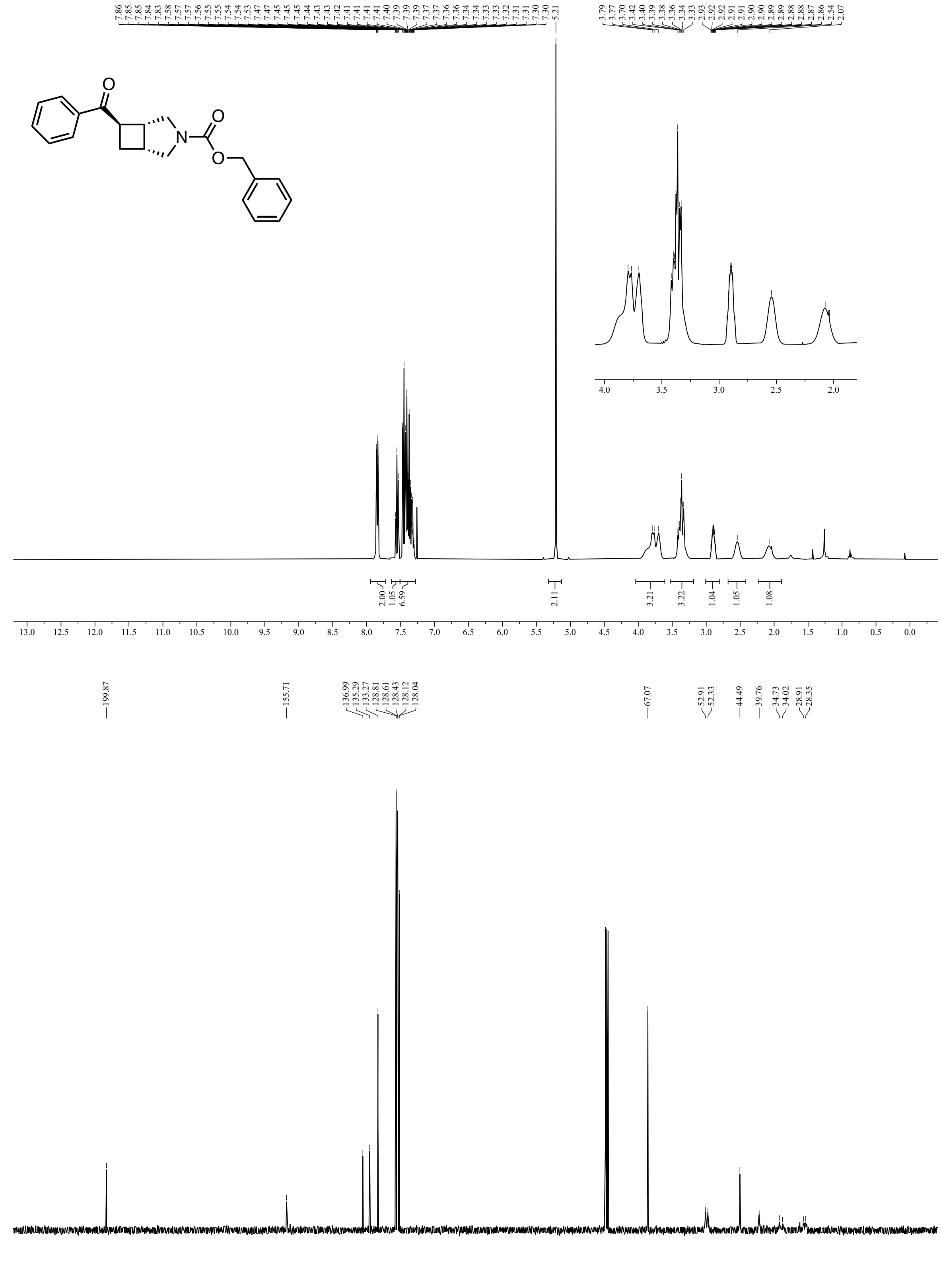

Figure S35: ^1H NMR (400 MHz, $CDCl_3$, top) and ^{13}C NMR (101 MHz, $CDCl_3$, bottom) spectra of **7c** recorded at 298 K.

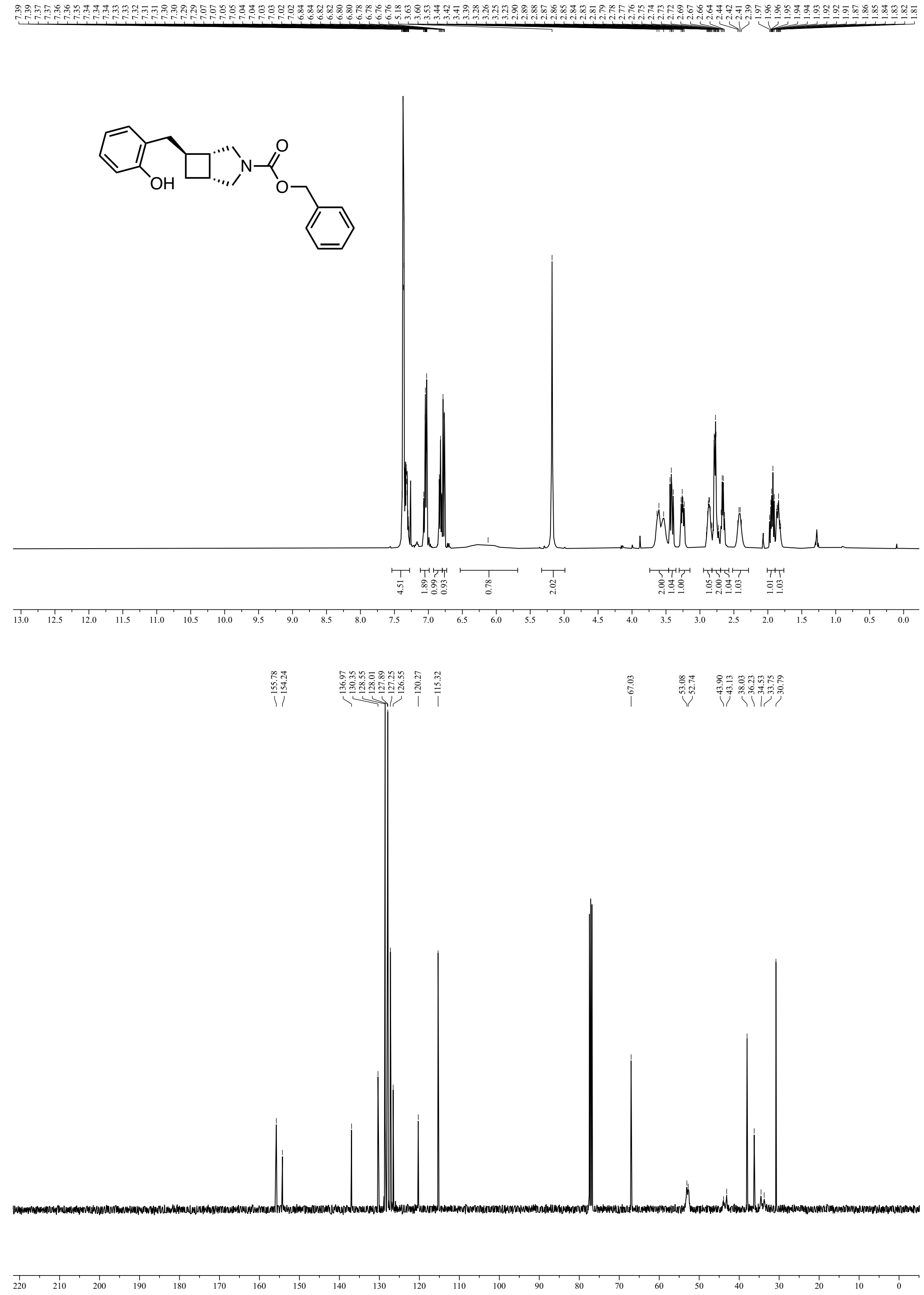

Figure S36: ^{1}H NMR (400 MHz, $CDCl_3$, top) and ^{13}C NMR (101 MHz, $CDCl_3$, bottom) spectra of **7d** recorded at 298 K.

4. SFC traces

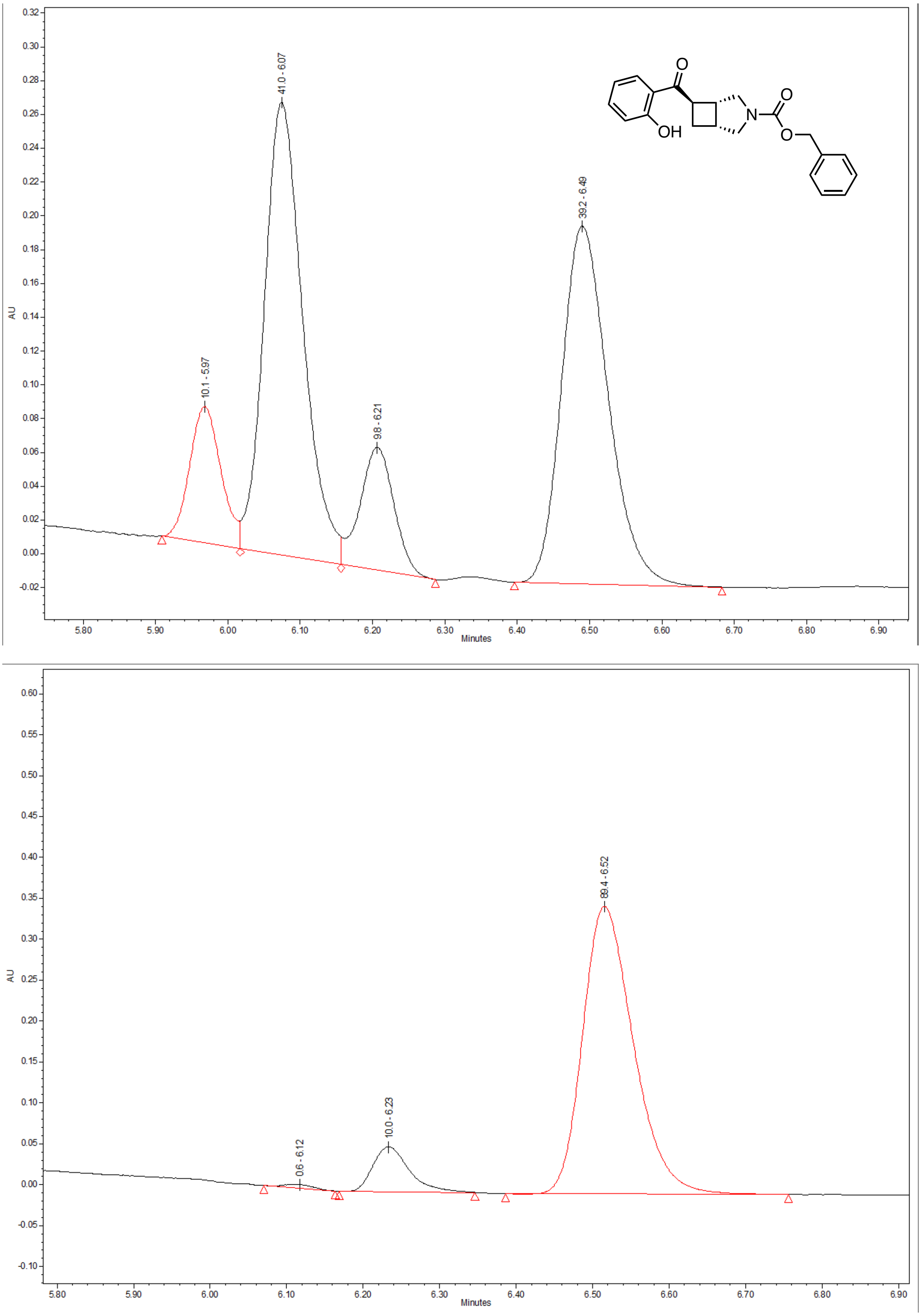

Figure S37: SFC trace of epimerized racemic (±)-**3a** and unpurified, enantioenriched (–)-**3a**.

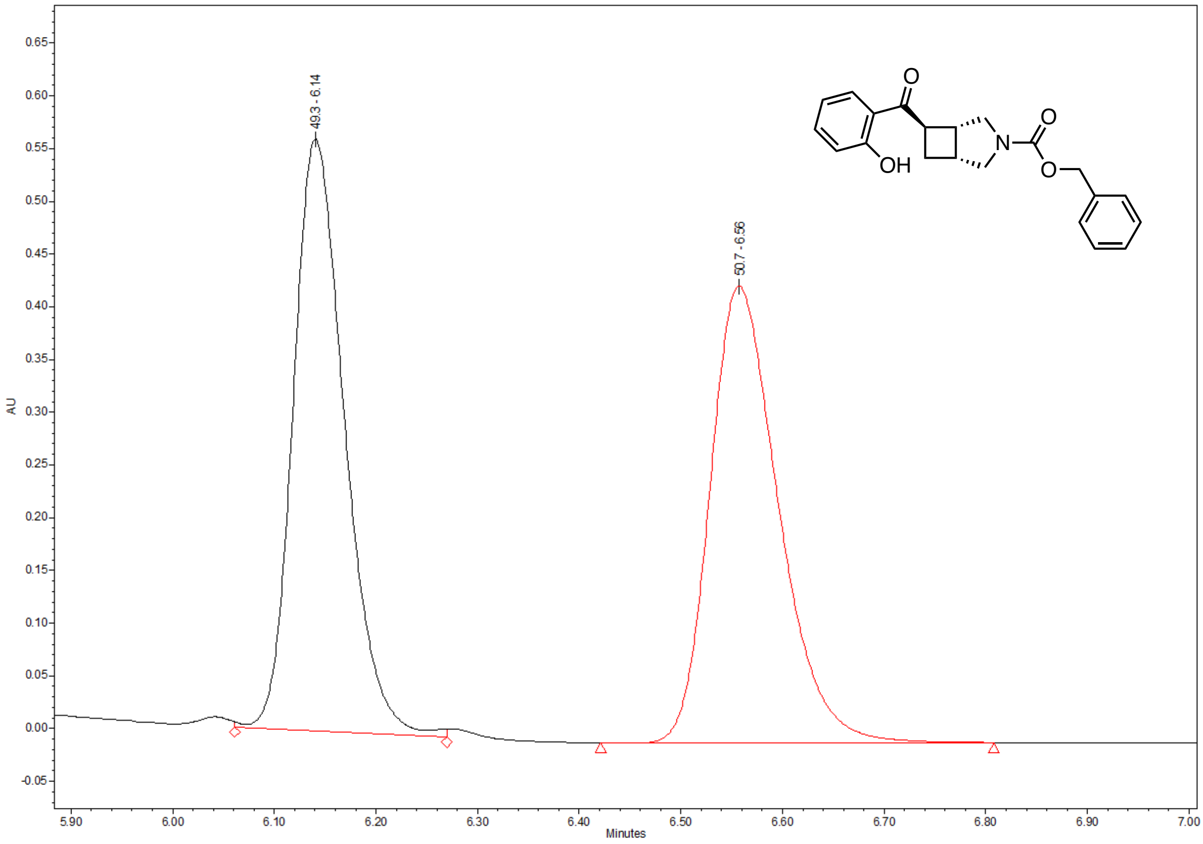

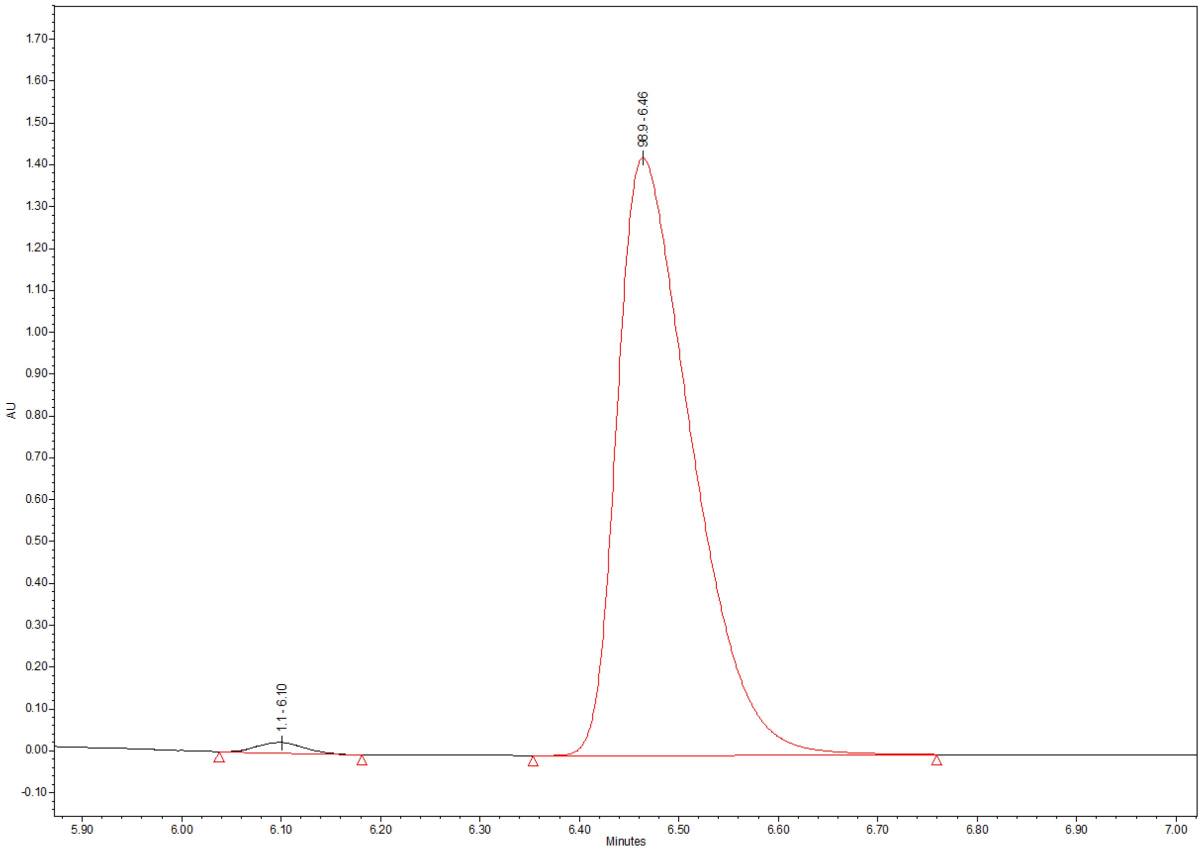

Figure S38: SFC trace of racemic (±)-**3a** and enantioenriched (–)-**3a**.

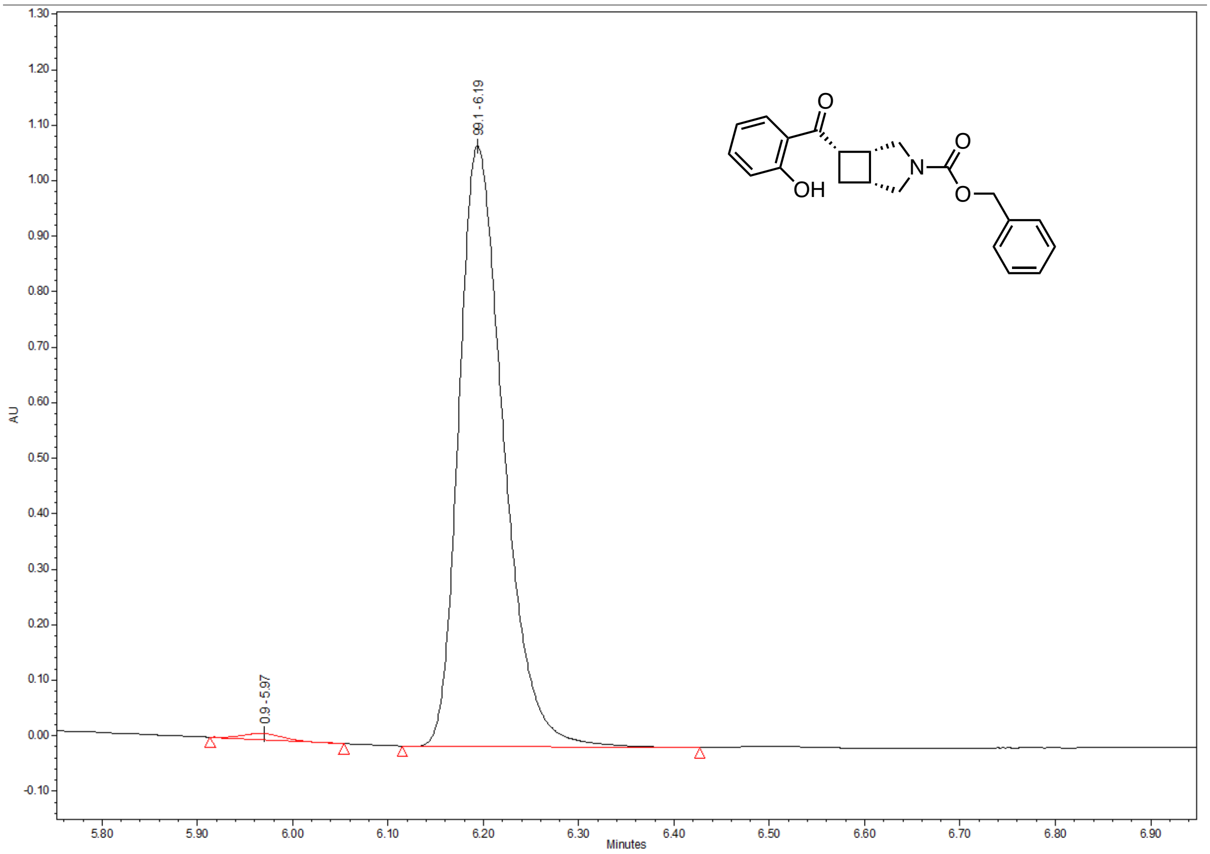

Figure S39: SFC trace of enantioenriched (–)-**3a'**.

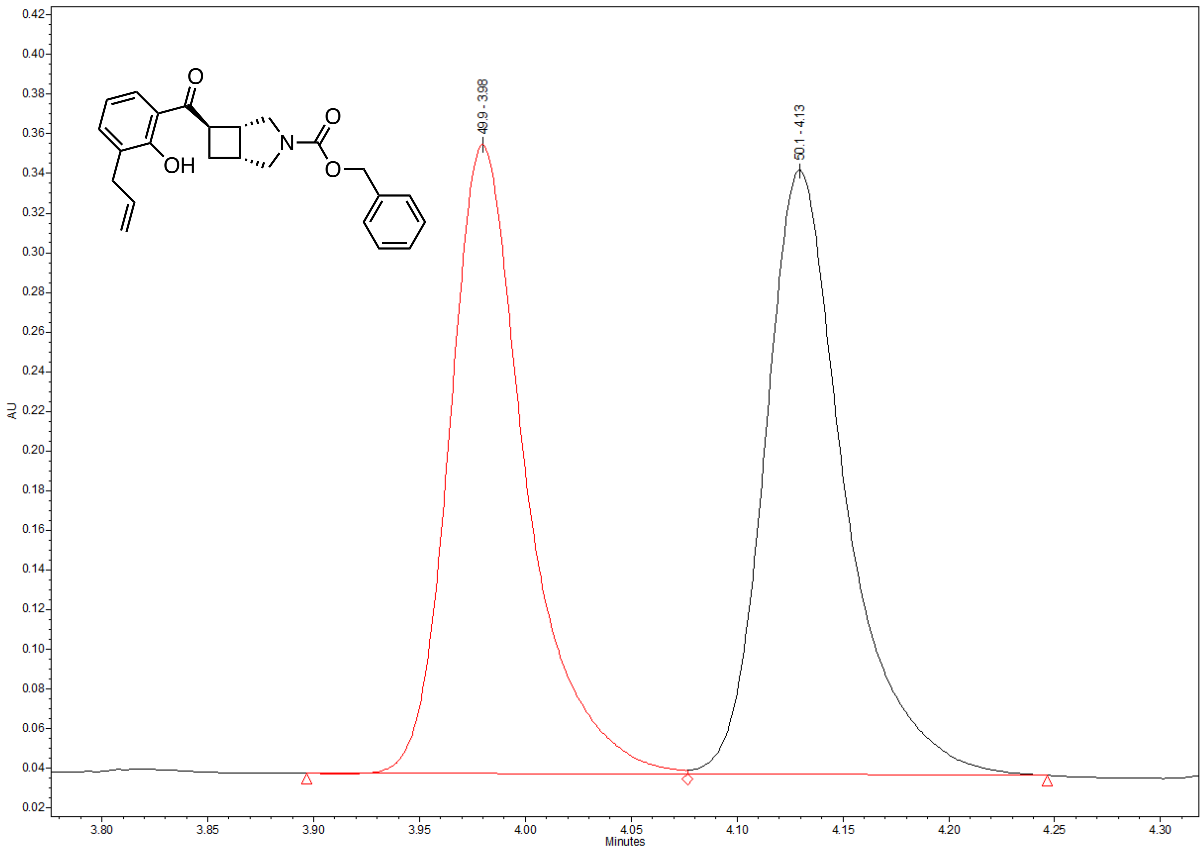

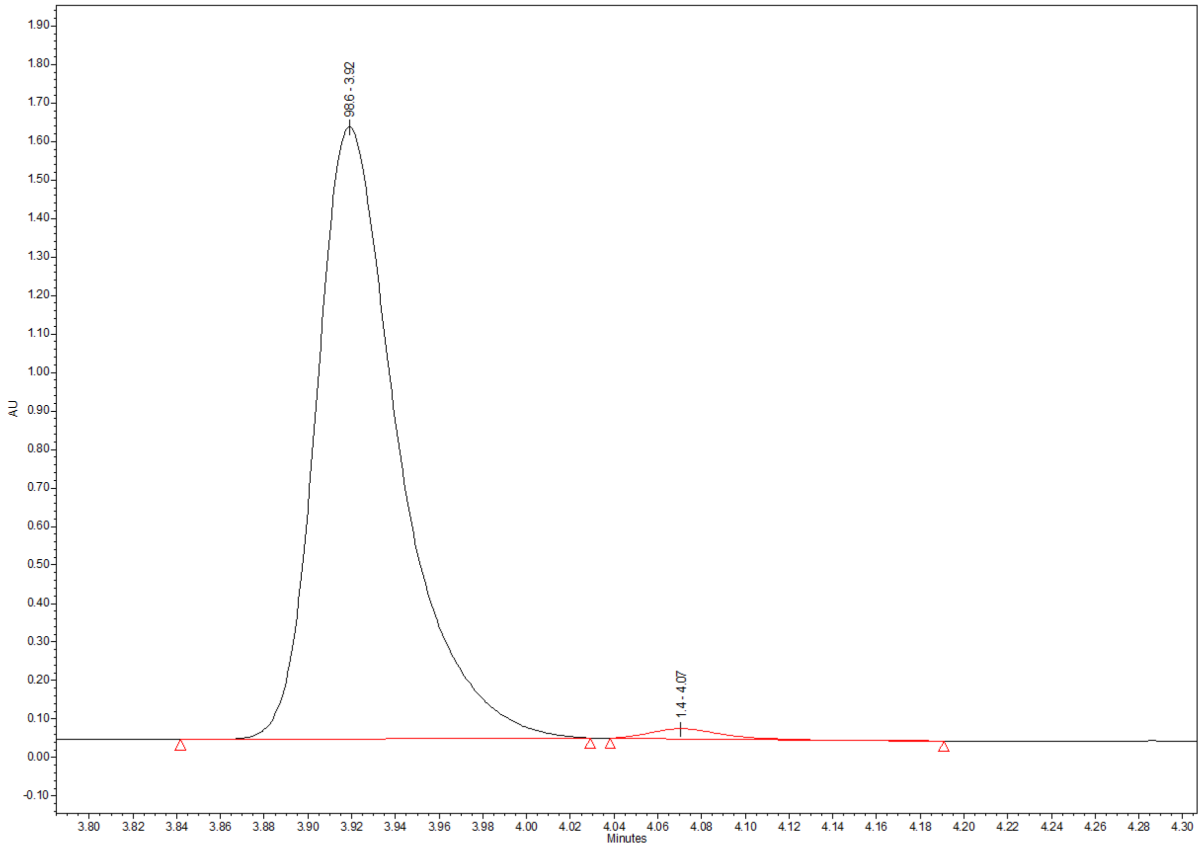

Figure S40: SFC trace of racemic (±)-**3b** and enantioenriched (–)-**3b**.

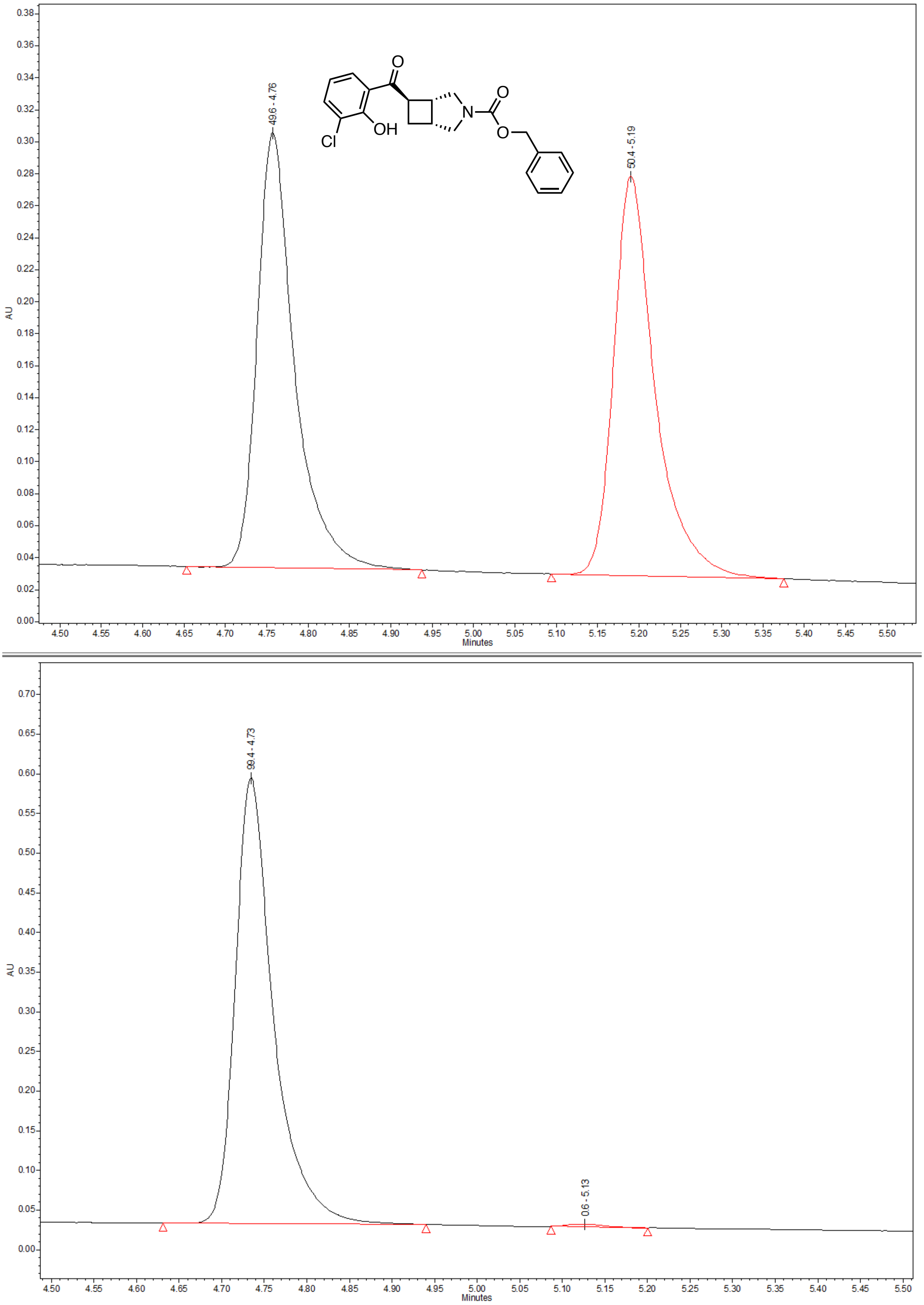

Figure S41: SFC trace of racemic (±)-**3c** and enantioenriched (–)-**3c**.

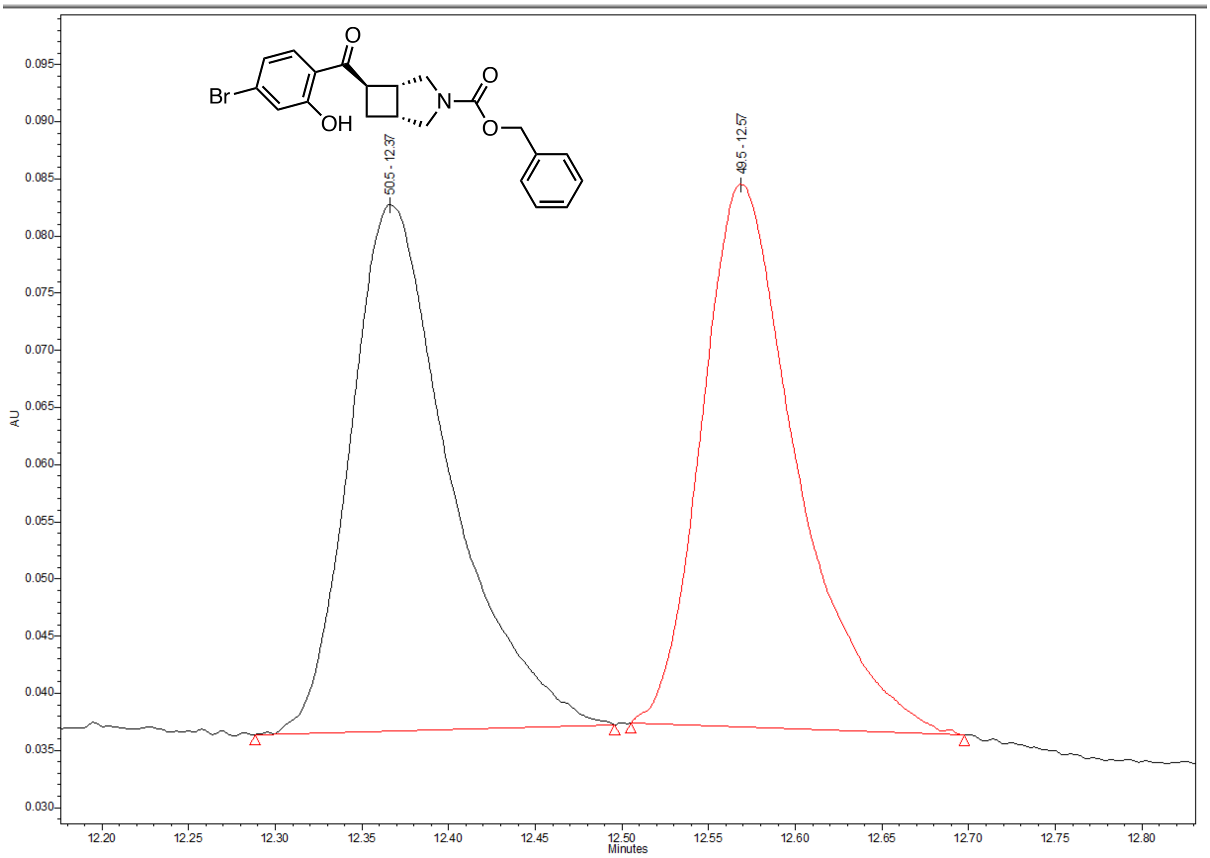

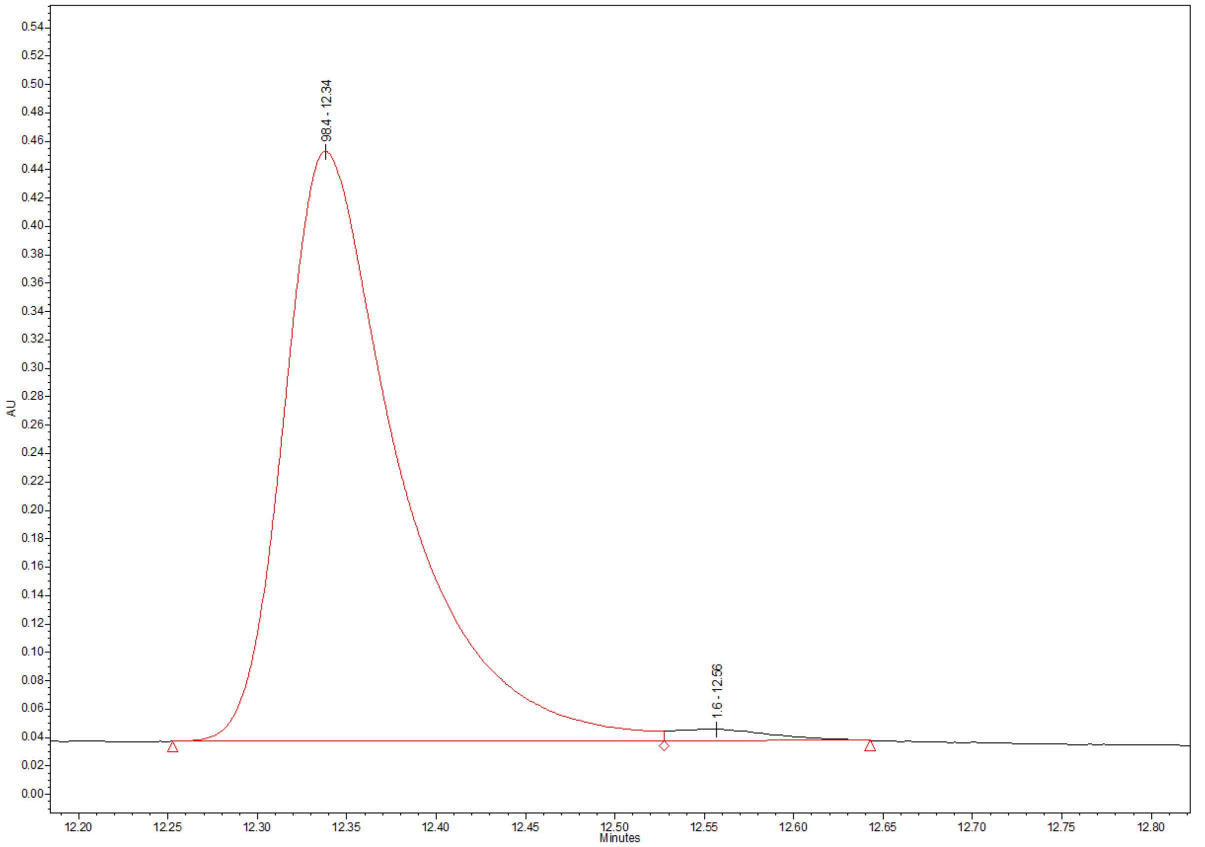

Figure S42: SFC trace of racemic (±)-**3d** and enantioenriched (–)-**3d**.

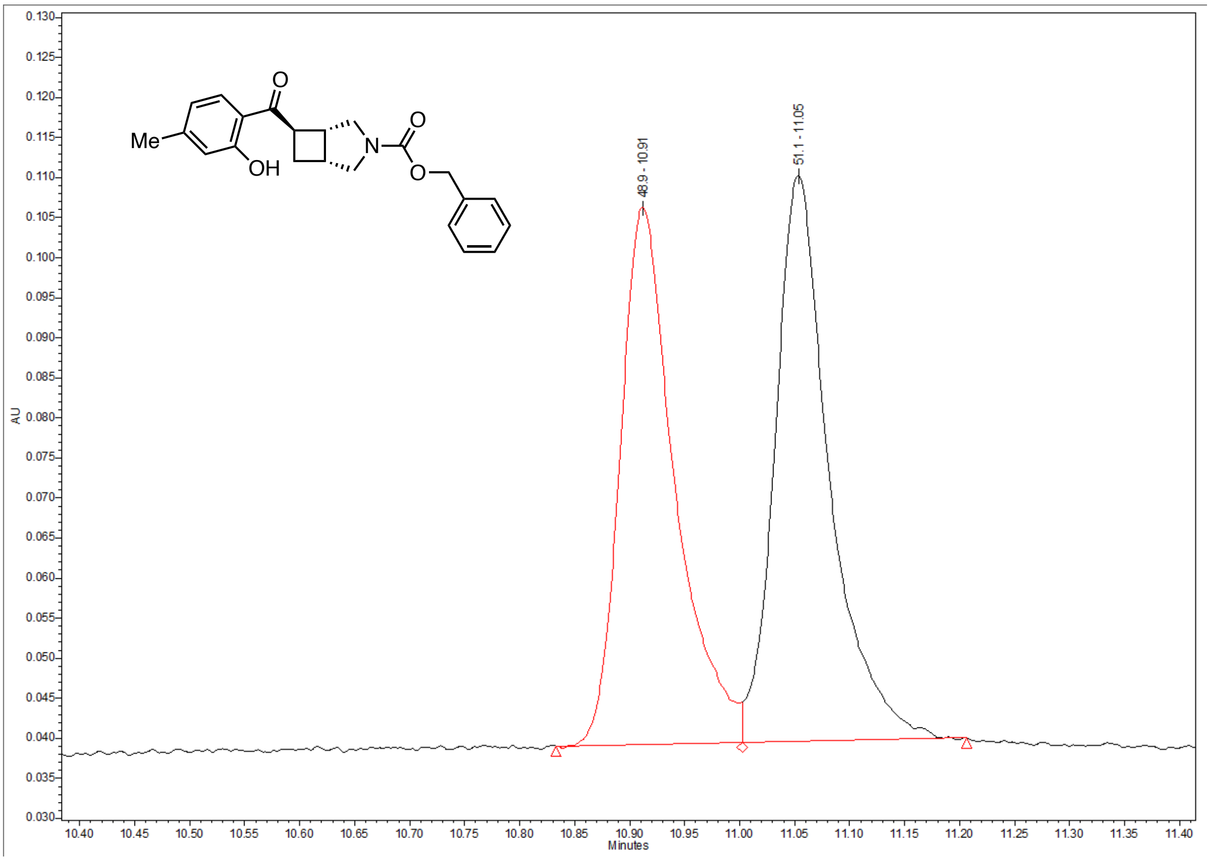

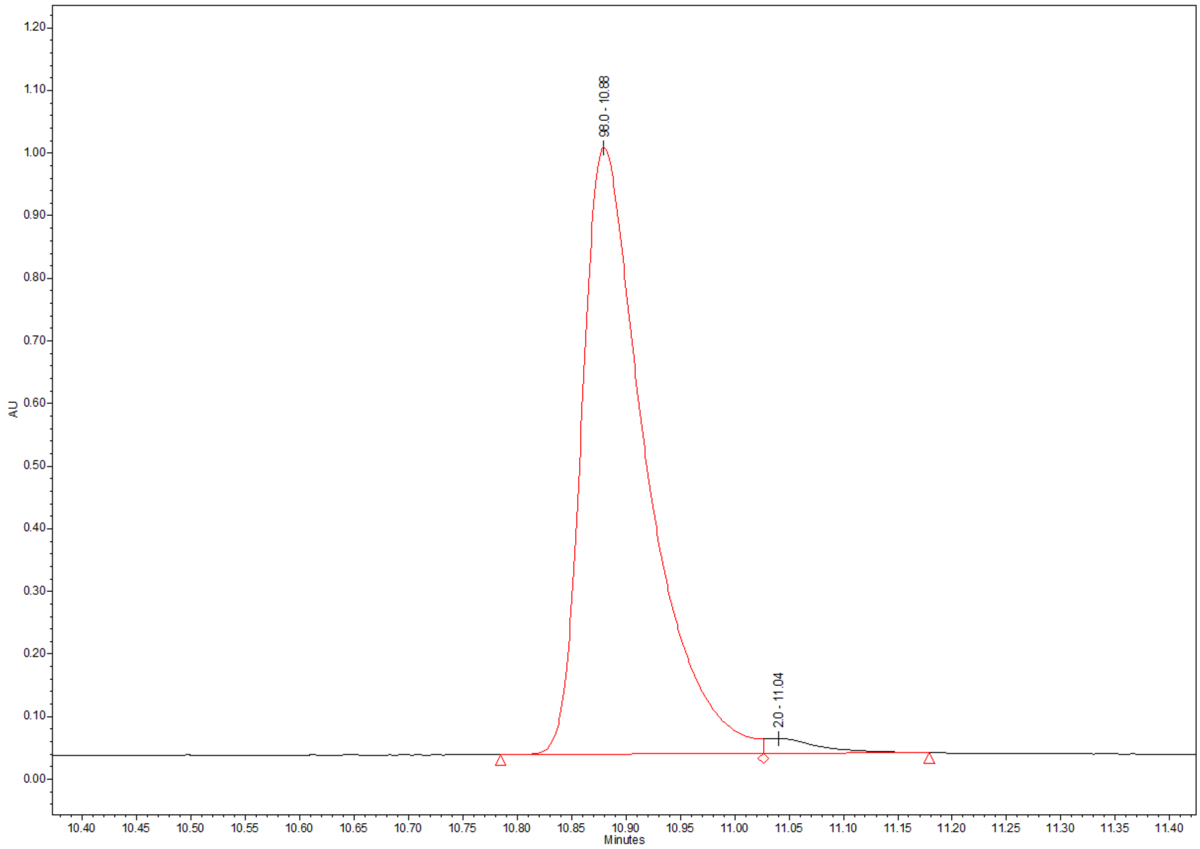

Figure S43: SFC trace of racemic (±)-**3e** and enantioenriched (–)-**3e**.

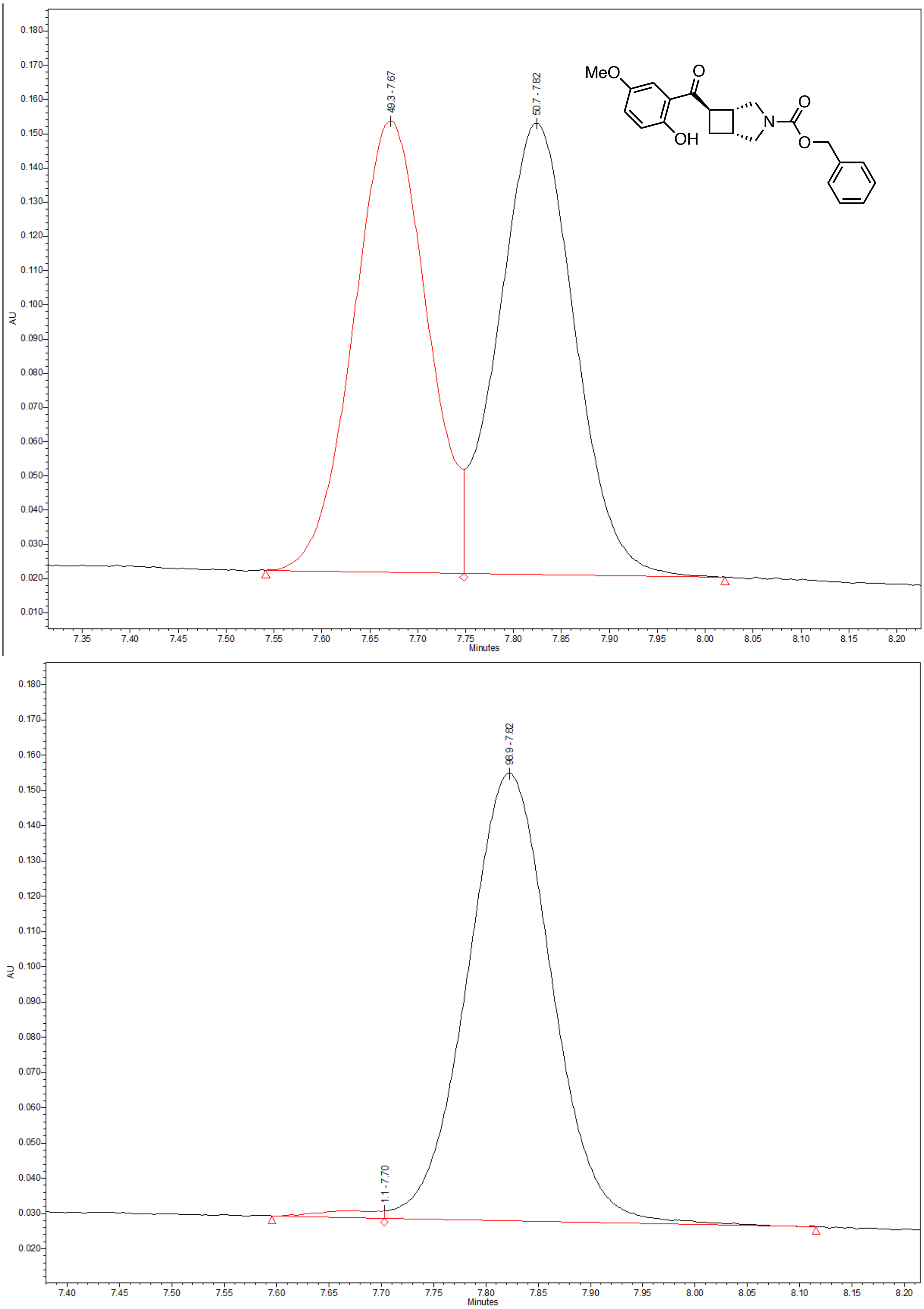

Figure S44: SFC trace of racemic (±)-**3f** and enantioenriched (–)-**3f**.

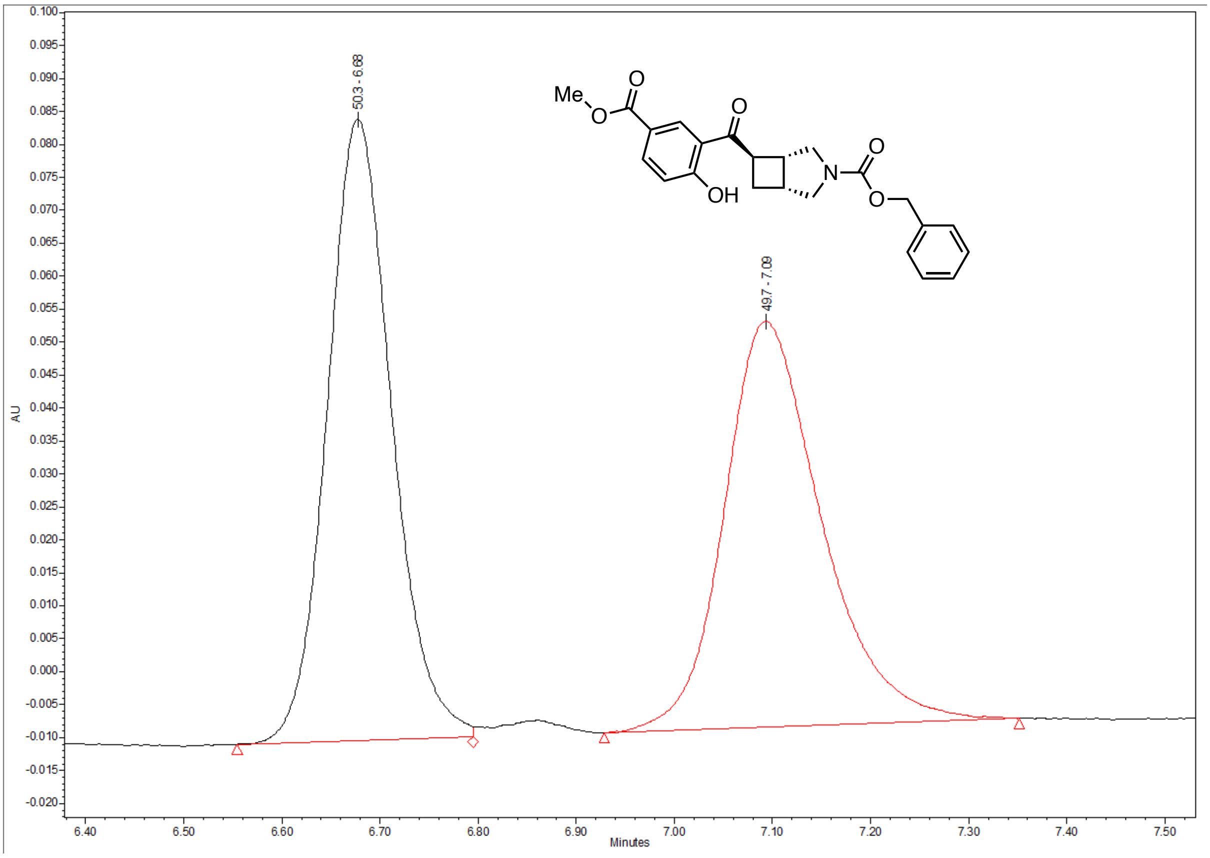

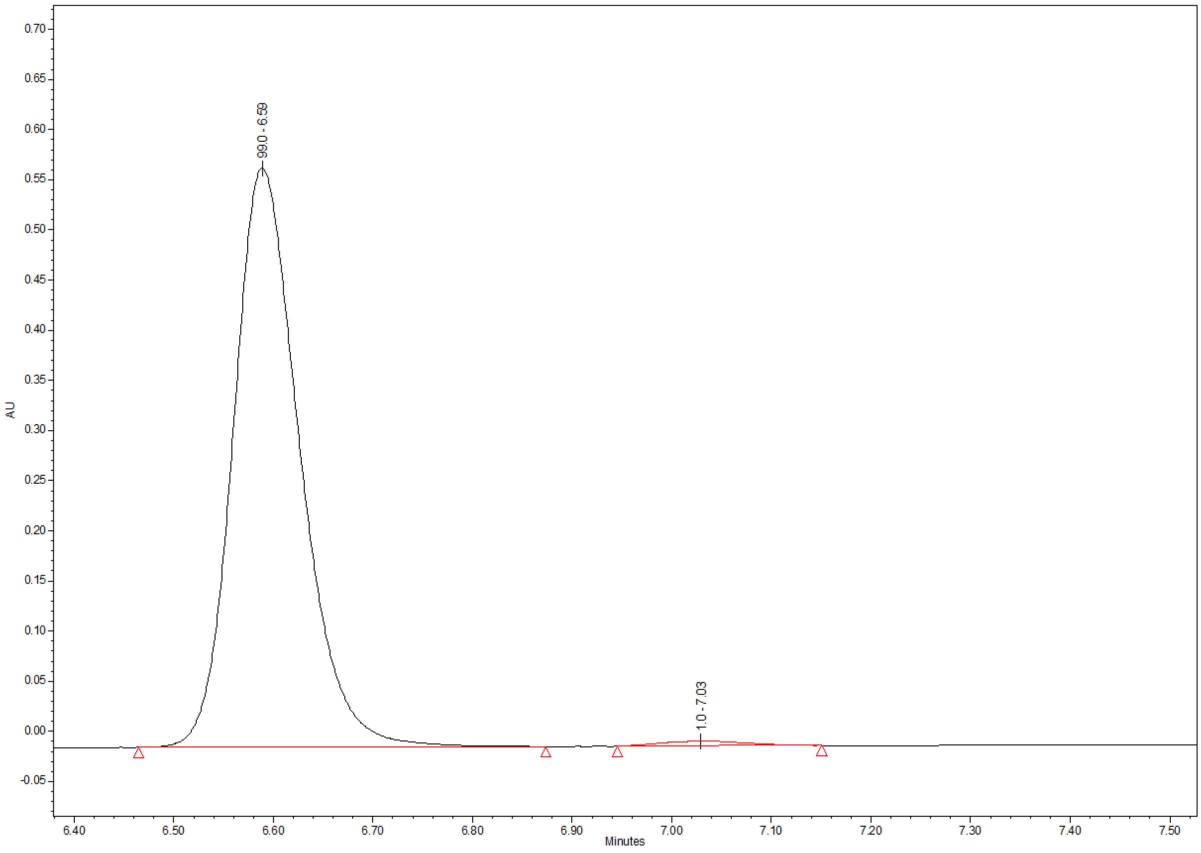

Figure S45: SFC trace of racemic (±)-**3g** and enantioenriched (–)-**3g**.

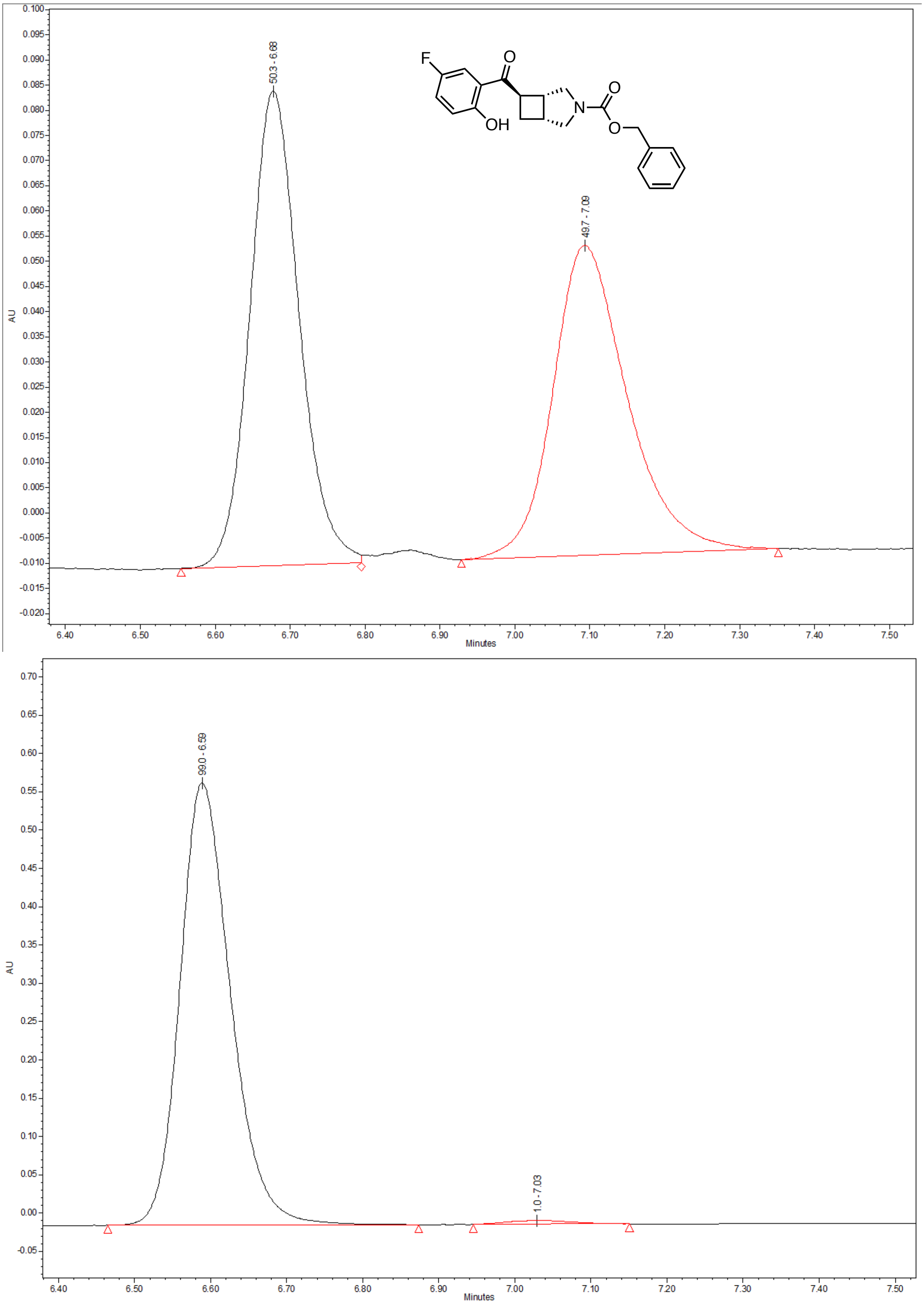

Figure S46: SFC trace of racemic (±)-**3h** and enantioenriched (–)-**3h**.

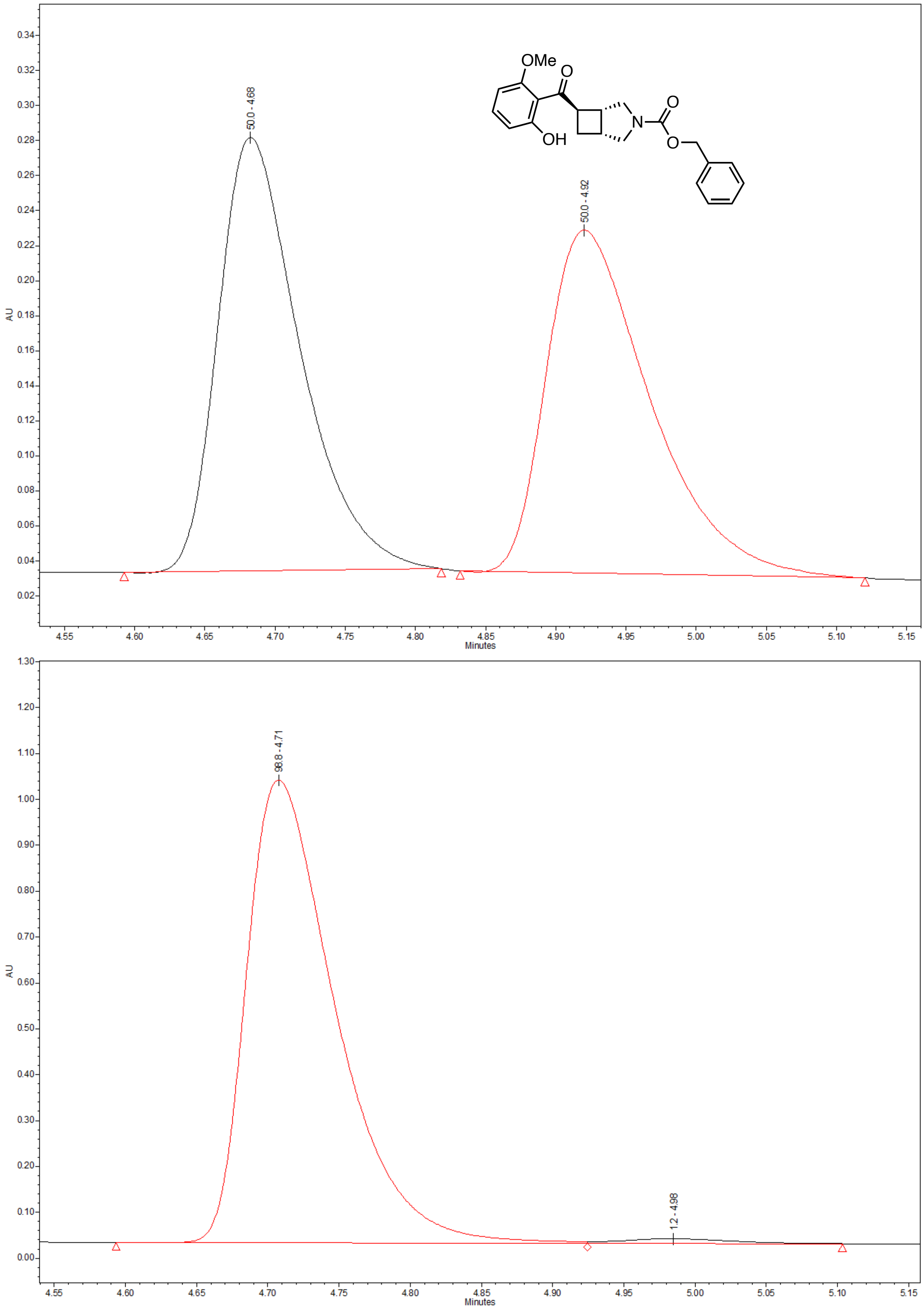

Figure S47: SFC trace of racemic (±)-**3i** and enantioenriched (–)-**3i**.

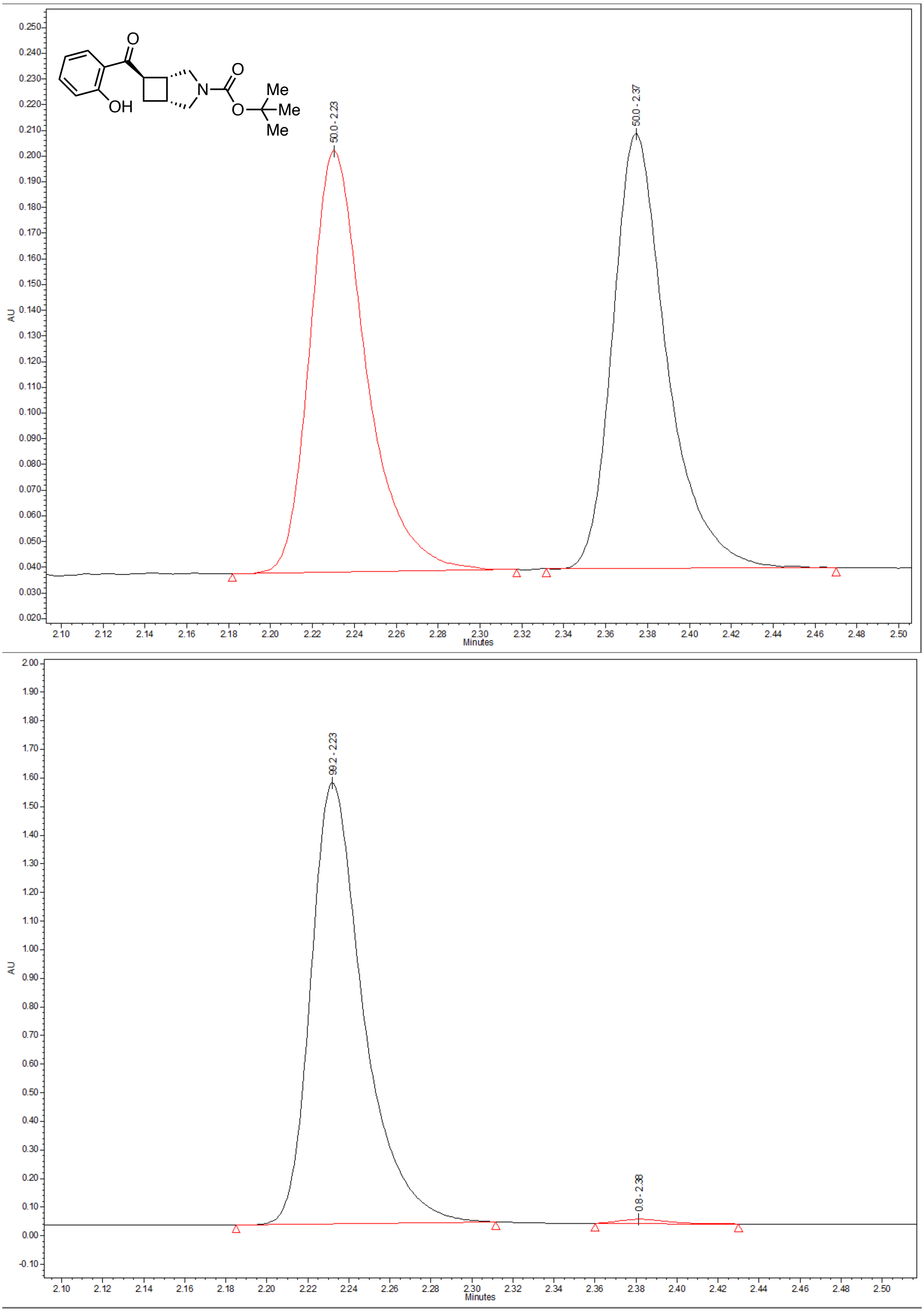

Figure S48: SFC trace of racemic (±)-**4a** and enantioenriched (–)-**4a**.

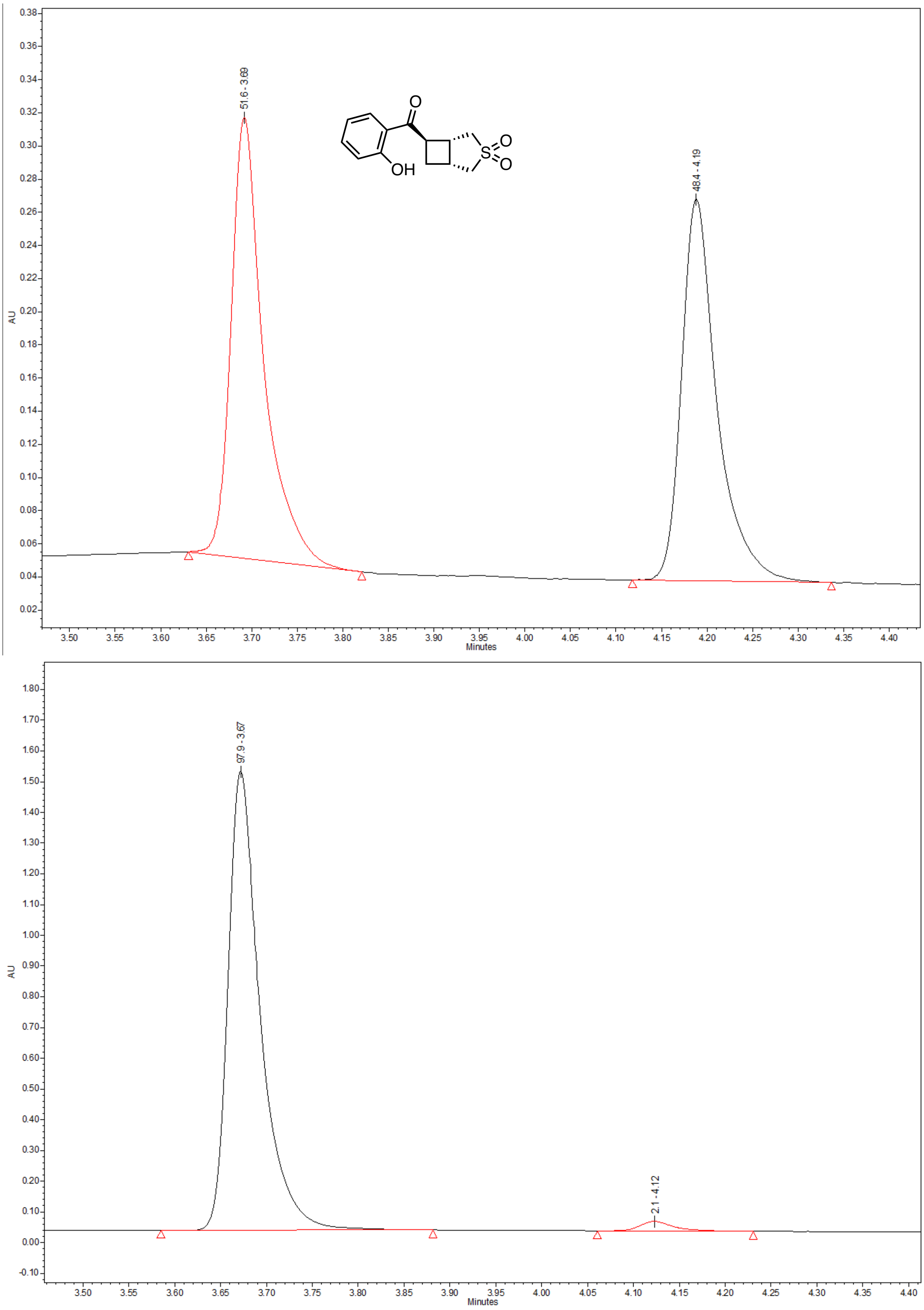

Figure S49: SFC trace of racemic (±)-**4b** and enantioenriched (–)-**4b**.

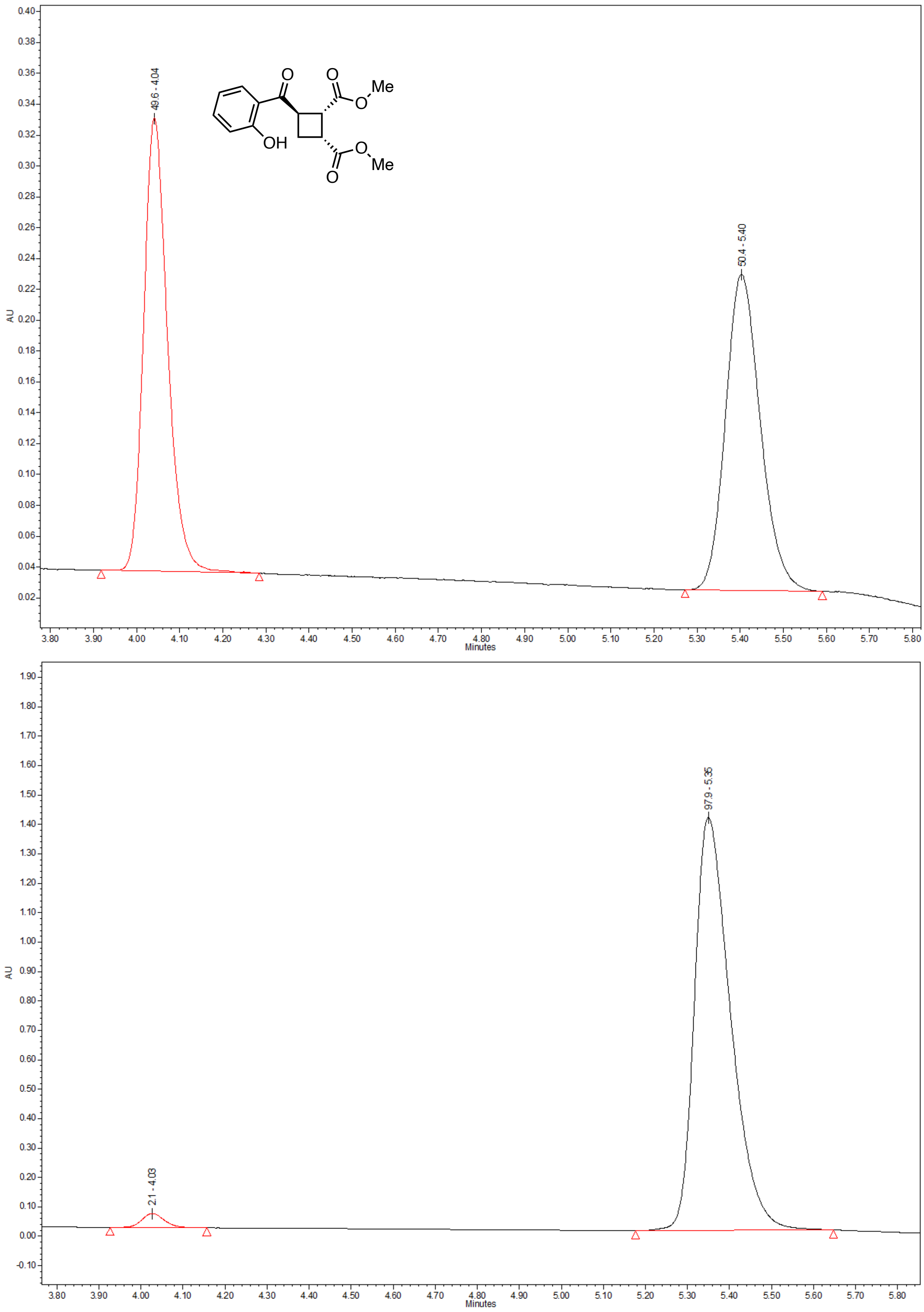

Figure S50: SFC trace of racemic (±)-**4c** and enantioenriched (–)-**4c**.

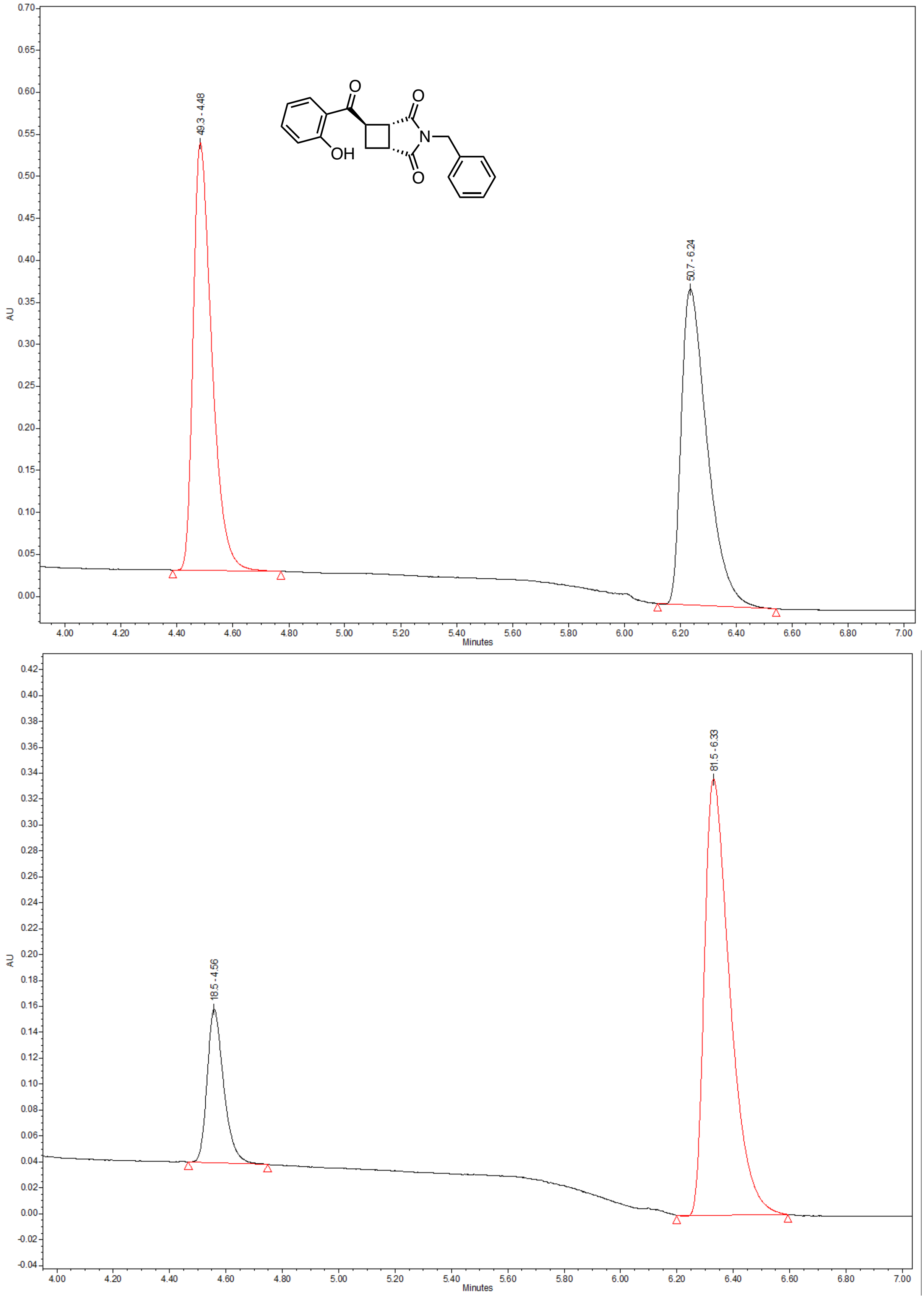

Figure S51: SFC trace of racemic (±)-**4d** and enantioenriched (–)-**4d**.

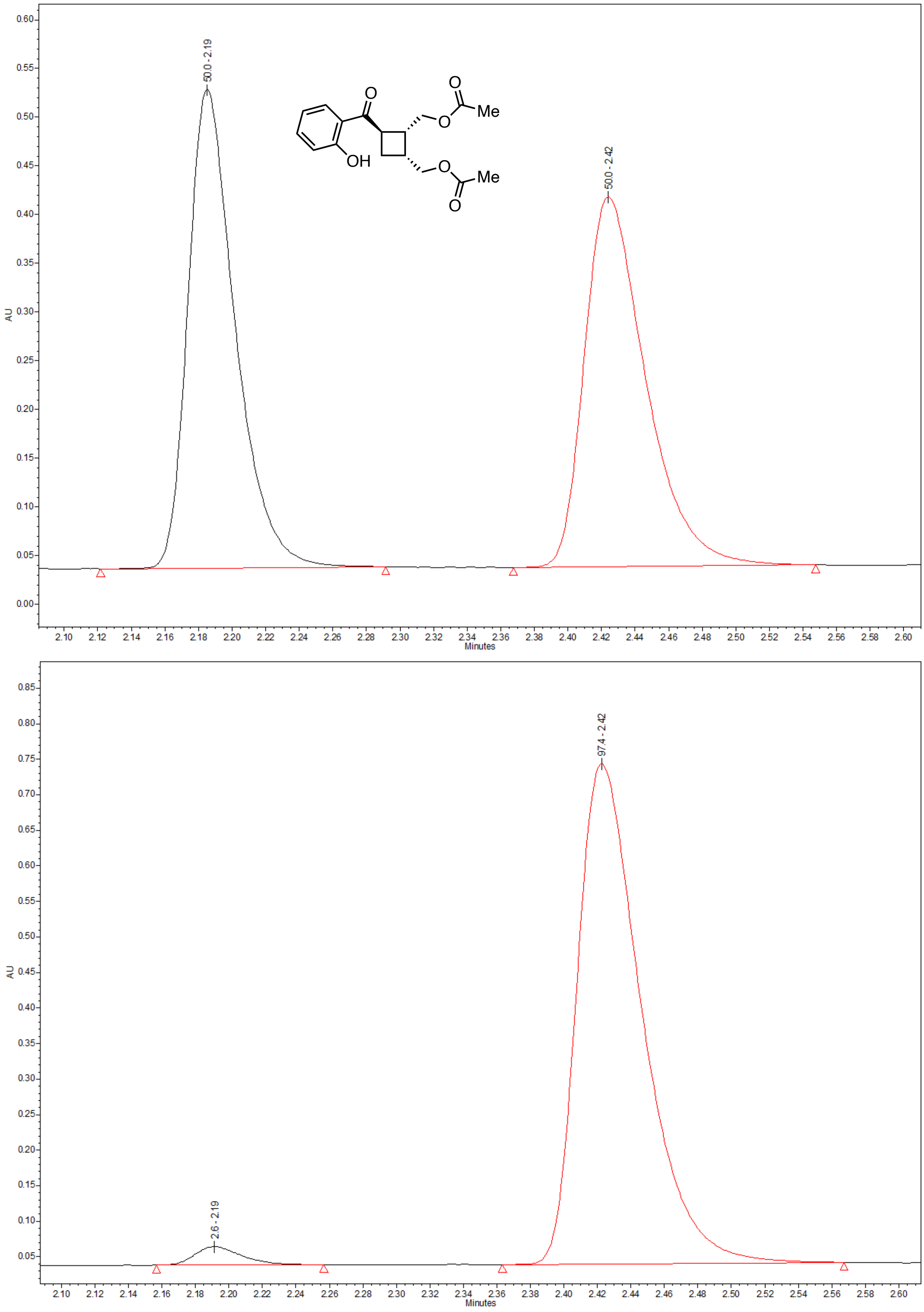

Figure S52: SFC trace of racemic (±)-**4e** and enantioenriched (–)-**4e**.

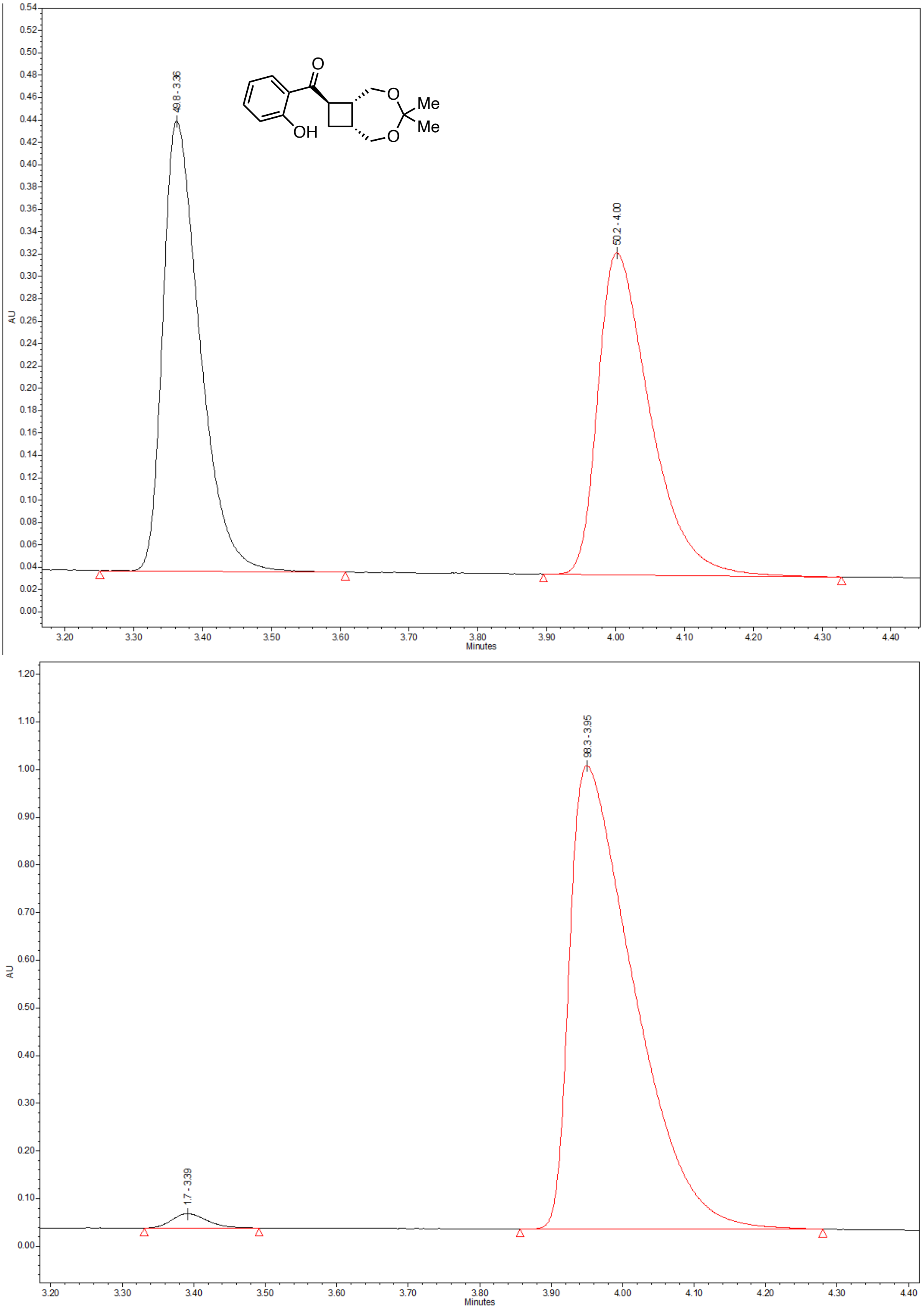

Figure S53: SFC trace of racemic (±)-**4f** and enantioenriched (–)-**4f**.

5. X-ray crystallographic analysis

The X-ray crystallographic analysis was performed by Dr. Curtis Moore (Ohio State University).

The single crystal X-ray diffraction studies were carried out on a Bruker Kappa Photon III CPAD diffractometer equipped with Mo K_α radiation (λ = 0.71073 Å). A 0.434 x 0.106 x 0.055 mm piece of a colorless rod was mounted on a Cryoloop with Paratone 24EX oil. Data were collected in a nitrogen gas stream at 100(2) K using ϕ and ϖ scans. Crystal-to-detector distance was 60 mm using variable exposure time (1s-20s) depending on θ with a scan width of 1.0°. Data collection was 99.6% complete to 25.00° in θ, 0.83Å. A total of 30549 reflections were collected covering the indices, -11<=h<=11, -8<=k<=8, -14<=l<=14. 2918 reflections were found to be symmetry independent, with a R_{int} of 0.0331. Indexing and unit cell refinement indicated a primitive, monoclinic lattice. The space group was found to be $P2_1$. The data were integrated using the Bruker SAINT software program and scaled using the SADABS software program. Solution by direct methods (SHELXT) produced a complete phasing model for refinement.

All nonhydrogen atoms were refined anisotropically by full-matrix least-squares (SHELXL-2014). All carbon bonded hydrogen atoms were placed using a riding model. Their positions were constrained relative to their parent atom using the appropriate HFIX command in SHELXL-2014. All other hydrogen atoms (H-bonding) were located in the difference map. Their relative positions were restrained using DFIX commands and their thermals freely refined. The absolute stereochemistry of the molecule was established by anomalous dispersion using the Parson's method with a Flack parameter of-0.025(19). Crystallographic data are summarized in Table S1.

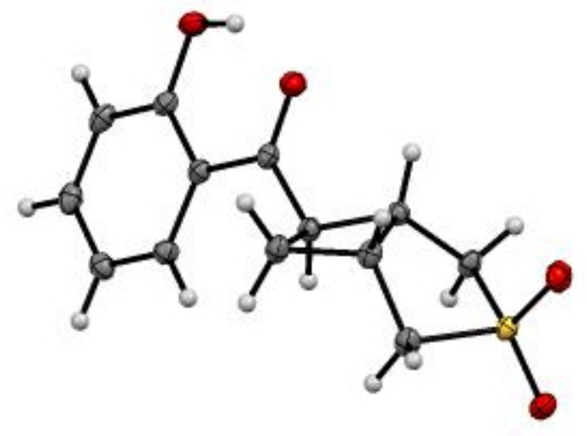

Figure S54: Ortep representation of **4b** with a probability level of 50% for the ellipsoids. (grey = carbon, white = hydrogen, yellow = sulfur).

Table S1: Crystal data and structure refinement for **4b**. (CCDC deposition number 2116804).

Empirical formula	C13 H14 O4 S	
Molecular formula	C13 H14 O4 S	
Formula weight	266.30	
Temperature	100.0 K	
Wavelength	0.71073 Å	
Crystal system	Monoclinic	
Space group	P 1 21 1	
Unit cell dimensions	a = 8.6416(9) Å	a = 90°.
	b = 6.2775(6) Å	b = 90°.
	c = 10.9475(12) Å	g = 90°.
Volume	589.90(11) $Å^3$	
Z	2	
Density (calculated)	1.499 Mg/m^3	
Absorption coefficient	0.278 mm^{-1}	
F(000)	280	
Crystal size	0.434 x 0.106 x 0.055 mm^3	
Crystal color, habit	Colorless Rod	
Theta range for data collection	3.189 to 28.301°.	
Index ranges	-11<=h<=11, -8<=k<=8, -14<=l<=14	
Reflections collected	30549	
Independent reflections	2918 [R(int) = 0.0331, R(sigma) = 0.0170]	
Completeness to theta = 25.000°	99.6 %	
Absorption correction	Semi-empirical from equivalents	
Max. and min. transmission	0.2627 and 0.2279	
Refinement method	Full-matrix least-squares on F^2	
Data / restraints / parameters	2918 / 2 / 167	
Goodness-of-fit on F^2	1.098	
Final R indices [I>2sigma(I)]	R1 = 0.0229, wR2 = 0.0634	
R indices (all data)	R1 = 0.0234, wR2 = 0.0639	
Absolute structure parameter	-0.025(19)	
Extinction coefficient	n/a	
Largest diff. peak and hole	0.283 and -0.235 e.$Å^{-3}$	

Table S2: Atomic coordinates (x 10^4) and equivalent isotropic displacement parameters (Å^{2}x 10^3) for **4b**. U(eq) is defined as one third of the trace of the orthogonalized U^{ij} tensor.

	x	y	z	U(eq)
S(1)	3290(1)	8164(1)	10155(1)	15(1)
O(1)	1042(2)	6606(2)	5377(1)	20(1)
O(2)	1405(2)	5160(2)	3237(1)	22(1)
O(3)	2316(2)	9977(2)	10358(1)	23(1)
O(4)	4728(2)	7921(3)	10967(1)	22(1)
C(1)	3605(2)	8153(4)	8574(1)	17(1)
C(2)	2077(2)	7255(3)	7921(2)	15(1)
C(3)	1080(2)	6140(3)	8856(2)	17(1)
C(4)	2122(2)	5825(3)	10069(2)	19(1)
C(5)	889(2)	4174(3)	7993(2)	20(1)
C(6)	2190(2)	5095(3)	7257(2)	16(1)
C(7)	1819(2)	5128(3)	5874(2)	15(1)
C(8)	2394(2)	3397(3)	5138(2)	15(1)
C(9)	2172(2)	3523(3)	3837(2)	16(1)
C(10)	2764(2)	1917(3)	3137(2)	20(1)
C(11)	3528(2)	191(3)	3704(2)	21(1)
C(12)	3739(2)	20(3)	4989(2)	19(1)
C(13)	3179(2)	1621(3)	5688(2)	17(1)

Table S3: Bond lengths [Å] and angles [°] for **4b**.

S(1)-O(3)	1.4478(15)
S(1)-O(4)	1.4500(13)
S(1)-C(1)	1.7830(16)
S(1)-C(4)	1.778(2)
O(1)-C(7)	1.234(2)
O(2)-H(2)	0.89(2)
O(2)-C(9)	1.351(2)
C(1)-H(1A)	0.9900
C(1)-H(1B)	0.9900
C(1)-C(2)	1.534(2)
C(2)-H(2A)	1.0000
C(2)-C(3)	1.577(3)
C(2)-C(6)	1.547(3)
C(3)-H(3)	1.0000
C(3)-C(4)	1.529(3)
C(3)-C(5)	1.552(3)
C(4)-H(4A)	0.9900
C(4)-H(4B)	0.9900
C(5)-H(5A)	0.9900
C(5)-H(5B)	0.9900
C(5)-C(6)	1.567(3)
C(6)-H(6)	1.0000
C(6)-C(7)	1.511(2)
C(7)-C(8)	1.473(2)
C(8)-C(9)	1.417(2)
C(8)-C(13)	1.404(3)
C(9)-C(10)	1.398(3)
C(10)-H(10)	0.9500
C(10)-C(11)	1.379(3)
C(11)-H(11)	0.9500
C(11)-C(12)	1.401(3)

C(12)-H(12)	0.9500
C(12)-C(13)	1.384(3)
C(13)-H(13)	0.9500
O(3)-S(1)-O(4)	117.45(9)
O(3)-S(1)-C(1)	107.98(10)
O(3)-S(1)-C(4)	108.70(9)
O(4)-S(1)-C(1)	112.25(8)
O(4)-S(1)-C(4)	112.74(10)
C(4)-S(1)-C(1)	95.46(9)
C(9)-O(2)-H(2)	109(3)
S(1)-C(1)-H(1A)	111.1
S(1)-C(1)-H(1B)	111.1
H(1A)-C(1)-H(1B)	109.0
C(2)-C(1)-S(1)	103.55(12)
C(2)-C(1)-H(1A)	111.1
C(2)-C(1)-H(1B)	111.1
C(1)-C(2)-H(2A)	112.6
C(1)-C(2)-C(3)	111.47(14)
C(1)-C(2)-C(6)	116.36(16)
C(3)-C(2)-H(2A)	112.6
C(6)-C(2)-H(2A)	112.6
C(6)-C(2)-C(3)	89.10(14)
C(2)-C(3)-H(3)	113.7
C(4)-C(3)-C(2)	108.08(15)
C(4)-C(3)-H(3)	113.7
C(4)-C(3)-C(5)	115.90(17)
C(5)-C(3)-C(2)	89.14(14)
C(5)-C(3)-H(3)	113.7
S(1)-C(4)-H(4A)	111.3
S(1)-C(4)-H(4B)	111.3
C(3)-C(4)-S(1)	102.30(13)
C(3)-C(4)-H(4A)	111.3

C(3)-C(4)-H(4B)	111.3
H(4A)-C(4)-H(4B)	109.2
C(3)-C(5)-H(5A)	113.8
C(3)-C(5)-H(5B)	113.8
C(3)-C(5)-C(6)	89.29(14)
H(5A)-C(5)-H(5B)	111.0
C(6)-C(5)-H(5A)	113.8
C(6)-C(5)-H(5B)	113.8
C(2)-C(6)-C(5)	89.69(14)
C(2)-C(6)-H(6)	111.1
C(5)-C(6)-H(6)	111.1
C(7)-C(6)-C(2)	116.17(16)
C(7)-C(6)-C(5)	116.02(15)
C(7)-C(6)-H(6)	111.1
O(1)-C(7)-C(6)	119.54(17)
O(1)-C(7)-C(8)	120.98(16)
C(8)-C(7)-C(6)	119.48(16)
C(9)-C(8)-C(7)	119.57(17)
C(13)-C(8)-C(7)	121.89(16)
C(13)-C(8)-C(9)	118.53(16)
O(2)-C(9)-C(8)	122.18(17)
O(2)-C(9)-C(10)	118.17(16)
C(10)-C(9)-C(8)	119.65(17)
C(9)-C(10)-H(10)	119.8
C(11)-C(10)-C(9)	120.42(18)
C(11)-C(10)-H(10)	119.8
C(10)-C(11)-H(11)	119.6
C(10)-C(11)-C(12)	120.81(18)
C(12)-C(11)-H(11)	119.6
C(11)-C(12)-H(12)	120.5
C(13)-C(12)-C(11)	119.07(18)
C(13)-C(12)-H(12)	120.5

C(8)-C(13)-H(13)	119.3
C(12)-C(13)-C(8)	121.49(17)
C(12)-C(13)-H(13)	119.3

Table S4: Anisotropic displacement parameters (Å^{2}x 10^3) for **4b**. The anisotropic displacement factor exponent takes the form: $-2\pi^2[h^2 a^{*2}U^{11} + ... + 2 h k a^* b^* U^{12}]$.

	U^{11}	U^{22}	U^{33}	U^{23}	U^{13}	U^{12}
S(1)	17(1)	16(1)	13(1)	-3(1)	2(1)	0(1)
O(1)	22(1)	20(1)	18(1)	-2(1)	-1(1)	5(1)
O(2)	26(1)	24(1)	16(1)	1(1)	-2(1)	6(1)
O(3)	25(1)	22(1)	23(1)	-8(1)	3(1)	3(1)
O(4)	22(1)	23(1)	18(1)	-2(1)	-3(1)	0(1)
C(1)	19(1)	20(1)	13(1)	-2(1)	3(1)	-5(1)
C(2)	16(1)	15(1)	14(1)	-2(1)	2(1)	0(1)
C(3)	15(1)	19(1)	18(1)	-3(1)	4(1)	-2(1)
C(4)	21(1)	20(1)	16(1)	-1(1)	5(1)	-4(1)
C(5)	22(1)	19(1)	17(1)	-3(1)	2(1)	-5(1)
C(6)	16(1)	16(1)	14(1)	-2(1)	1(1)	1(1)
C(7)	14(1)	17(1)	15(1)	-2(1)	1(1)	-1(1)
C(8)	14(1)	16(1)	14(1)	0(1)	2(1)	-2(1)
C(9)	15(1)	19(1)	15(1)	-1(1)	0(1)	-2(1)
C(10)	19(1)	26(1)	15(1)	-4(1)	2(1)	-2(1)
C(11)	18(1)	22(1)	23(1)	-8(1)	4(1)	0(1)
C(12)	19(1)	17(1)	22(1)	0(1)	3(1)	2(1)
C(13)	18(1)	18(1)	15(1)	1(1)	2(1)	-1(1)
